# Digoxin for atrial fibrillation and atrial flutter: A systematic review with meta-analysis and trial sequential analysis of randomised clinical trials

**DOI:** 10.1371/journal.pone.0193924

**Published:** 2018-03-08

**Authors:** Naqash J. Sethi, Emil E. Nielsen, Sanam Safi, Joshua Feinberg, Christian Gluud, Janus C. Jakobsen

**Affiliations:** 1 Copenhagen Trial Unit, Centre for Clinical Intervention Research, Department, Rigshospitalet, Copenhagen University Hospital, Copenhagen, Denmark; 2 The Cochrane Hepato-Biliary Group, Copenhagen Trial Unit, Centre for Clinical Intervention Research, Department, Rigshospitalet, Copenhagen University Hospital, Copenhagen, Denmark; 3 Department of Cardiology, Holbæk Hospital, Holbæk, Denmark; University of Miami School of Medicine, UNITED STATES

## Abstract

**Background:**

During recent years, systematic reviews of observational studies have compared digoxin to no digoxin in patients with atrial fibrillation or atrial flutter, and the results of these reviews suggested that digoxin seems to increase the risk of all-cause mortality regardless of concomitant heart failure. Our objective was to assess the benefits and harms of digoxin for atrial fibrillation and atrial flutter based on randomized clinical trials.

**Methods:**

We searched CENTRAL, MEDLINE, Embase, LILACS, SCI-Expanded, BIOSIS for eligible trials comparing digoxin versus placebo, no intervention, or other medical interventions in patients with atrial fibrillation or atrial flutter in October 2016. Our primary outcomes were all-cause mortality, serious adverse events, and quality of life. Our secondary outcomes were heart failure, stroke, heart rate control, and conversion to sinus rhythm. We performed both random-effects and fixed-effect meta-analyses and chose the more conservative result as our primary result. We used Trial Sequential Analysis (TSA) to control for random errors. We used GRADE to assess the quality of the body of evidence.

**Results:**

28 trials (n = 2223 participants) were included. All were at high risk of bias and reported only short-term follow-up. When digoxin was compared with all control interventions in one analysis, we found no evidence of a difference on all-cause mortality (risk ratio (RR), 0.82; TSA-adjusted confidence interval (CI), 0.02 to 31.2; I^2^ = 0%); serious adverse events (RR, 1.65; TSA-adjusted CI, 0.24 to 11.5; I^2^ = 0%); quality of life; heart failure (RR, 1.05; TSA-adjusted CI, 0.00 to 1141.8; I^2^ = 51%); and stroke (RR, 2.27; TSA-adjusted CI, 0.00 to 7887.3; I^2^ = 17%). Our analyses on acute heart rate control (within 6 hours of treatment onset) showed firm evidence of digoxin being superior compared with placebo (mean difference (MD), -12.0 beats per minute (bpm); TSA-adjusted CI, -17.2 to -6.76; I^2^ = 0%) and inferior compared with beta blockers (MD, 20.7 bpm; TSA-adjusted CI, 14.2 to 27.2; I^2^ = 0%). Meta-analyses on acute heart rate control showed that digoxin was inferior compared with both calcium antagonists (MD, 21.0 bpm; TSA-adjusted CI, -30.3 to 72.3) and with amiodarone (MD, 14.7 bpm; TSA-adjusted CI, -0.58 to 30.0; I^2^ = 42%), but in both comparisons TSAs showed that we lacked information. Meta-analysis on acute conversion to sinus rhythm showed that digoxin compared with amiodarone reduced the probability of converting atrial fibrillation to sinus rhythm, but TSA showed that we lacked information (RR, 0.54; TSA-adjusted CI, 0.13 to 2.21; I^2^ = 0%).

**Conclusions:**

The clinical effects of digoxin on all-cause mortality, serious adverse events, quality of life, heart failure, and stroke are unclear based on current evidence. Digoxin seems to be superior compared with placebo in reducing the heart rate, but inferior compared with beta blockers. The long-term effect of digoxin is unclear, as no trials reported long-term follow-up. More trials at low risk of bias and low risk of random errors assessing the clinical effects of digoxin are needed.

**Systematic review registration:**

PROSPERO CRD42016052935

## Introduction

Atrial fibrillation is the most common arrhythmia of the heart with a prevalence of approximately 2% in the western world [1, 2]. Atrial fibrillation and atrial flutter are both associated with an increased risk of morbidity and death [3–9]. The risks of both cerebral stroke and heart failure are increased nearly fivefold in patients with atrial fibrillation and atrial flutter, and about 20% of every stroke may be due to atrial fibrillation [3–8]. Atrial fibrillation and atrial flutter also have a significant impact on healthcare costs and account for approximately 1% of the National Health Service budget in the United Kingdom and approximately 26 billion dollars of annual expenses in the United States [10, 11]. In the management of atrial fibrillation and atrial flutter, it is often necessary to use medical interventions to lower the heart rate and consequently prevent excessive tachycardia and limit symptoms [12, 13]. Lowering the heart rate might also, theoretically, prevent the development of heart failure and tachycardia-mediated cardiomyopathy [14–16].

During recent years, systematic reviews of observational studies have compared digoxin to no digoxin (the latter participants usually receiving some other treatment for heart rate control) in patients with atrial fibrillation or atrial flutter, and these reviews suggested that digoxin seems to increase the risk of all-cause mortality regardless of concomitant heart failure [17–20]. The observed association in observational studies might be a result of confounding that cannot be mitigated by statistical adjustment (e.g., selection biases and prescription biases) [21, 22].

Therefore, the aim of this systematic review was to evaluate the beneficial and harmful effects of digoxin for atrial fibrillation and atrial flutter in randomized clinical trials [21, 22]. Furthermore, we have not identified any previous systematic review of randomized clinical trials comparing digoxin versus placebo, no intervention, or other medical interventions in patients with atrial fibrillation and atrial flutter.

## Methods

We conducted this systematic review based on the Preferred Reporting Items for Systematic Reviews and Meta-Analysis guidelines (PRISMA) (S1 Text) [23], and the updated Cochrane methodology used in this systematic review is described in detail in our protocol (S2 Text) [24–26], which was registered prior to the systematic literature search.

### Search strategy and selection criteria

We searched for randomized clinical trials comparing digoxin versus placebo, no intervention, or other medical interventions in patients with atrial fibrillation or atrial flutter. We searched for eligible trials in the Cochrane Central Register of Controlled Trials (CENTRAL), MEDLINE, Embase, LILACS, Science Citation Index Expanded on Web of Science, BIOSIS, Google Scholar, clinicaltrials.gov, Trip Medical Database (TRIP), WHO International Clinical Trials Registry Platform (ICTRP), International Standard Randomised Controlled Trial Number (ISRCTN), EU Clinical Trial Register (EUCTR), and Chinese Clinical Trial Registry (ChiCTR) in October 2016. The search strategy can be found in the supplementary material (S3 Text). Additionally, we checked the reference lists of relevant publications for any unidentified trials. Trials were included irrespective of trial design, setting, publication status, publication year, language, and reporting of one of our outcomes [25].

### Data extraction and risk of bias assessment

Three authors (NJS, JF, EEN) independently selected relevant trials, and four authors (NJS, SS, JF, EEN) independently extracted data in pairs using a standardized data extraction sheet and assessed the risk of bias according to the *Cochrane Handbook for Systematic Reviews of Interventions* and Lundh et al. [26, 27]. The data extraction sheet consisted of, e.g., trial characteristics, participants’ characteristics and diagnosis, intervention characteristics, control characteristics, co-intervention characteristics, and outcomes. We assessed the following risk of bias domains:

- random sequence generation;
- allocation concealment;
- blinding of participants and personnel;
- blinding of outcome assessment;
- incomplete outcome data;
- selective outcome reporting;
- funding bias; and
- other risks of bias.

These domains enable classification of randomized trials at low risk of bias and at high risk of bias. The latter trials tend to overestimate intervention effects of benefits and underestimate intervention effects of harms [27–33].

Any discrepancies in the data extraction and risk of bias assessment by the four authors (NJS, SS, JF, EEN) were discussed with a fifth review author (JCJ). We attempted to contact trial authors if relevant data were unclear or missing.

### Outcomes and subgroup analysis

Our primary outcomes were all-cause mortality, serious adverse events (as defined by the ICH guidelines) [34], and quality of life. Our secondary outcomes were heart failure, stroke, heart rate control, and conversion to sinus rhythm. We performed separate analyses for digoxin versus placebo and separate analyses for digoxin versus each individual medical intervention when assessing heart rate control and conversion to sinus rhythm. We used the trial results reported at maximal follow-up for all outcomes, except for heart rate control and conversion to sinus rhythm. For the latter outcomes, we used the trial results reported at two different time ranges (within 6 hours of treatment onset, and 6 to 24 hours after treatment onset). For heart rate control, we primarily assessed the participants who remained in atrial fibrillation and did not convert to sinus rhythm after randomisation. In a secondary analysis, we assessed all participants regardless of type of rhythm.

We planned the following subgroup analyses on our primary outcomes:

- trials at high risk of bias compared to trials at low risk of bias;
- comparison of different types of control interventions;
- comparison of participants with heart failure to participants without heart failure;
- comparison of trials administrating different medical co-interventions;
- comparison of different mean ages of participants;
- comparison of different durations of atrial fibrillation;
- comparison of participants with atrial fibrillation to participants with atrial flutter; and
- comparison of trials only randomizing men to trials only randomizing women.

### Assessment of statistical and clinical significance

We performed our meta-analyses according to the recommendations stated in the *Cochrane Handbook for Systematic Reviews of Interventions* [26], Keus et al. [35], and the eight-step assessment suggested by Jakobsen et al. [36] for better validation of meta-analytic results in systematic reviews. Review Manager 5 and Stata 15 were used for all meta-analyses [37, 38]. We used risk ratios (RR) for dichotomous outcomes and mean differences (MD) for continuous outcomes. We performed both random-effects (DerSimonian-Laird model) and fixed-effect meta-analyses with the Mantel-Haenszel method and chose the most conservative result as our primary result [36]. The more conservative result was the result with the highest P value and the widest 95% confidence interval (CI). If there was substantial discrepancy between the results of the two methods, we reported and discussed the results [36]. We used Trial Sequential Analysis (TSA) to control for random errors by estimating the diversity-adjusted required information size (DARIS) (that is the number of participants needed in a meta-analysis to detect or reject a certain intervention effect) [24, 25, 36, 39–48]. The DARIS is based on our predefined anticipated intervention effect. When analysing dichotomous outcomes, we pragmatically anticipated an intervention effect of 15% risk ratio reduction (RRR). When analysing continuous outcomes, we pragmatically anticipated an intervention effect equal to the MD of the observed standard difference (SD)/2 [25, 49]. Based on the DARIS, trial sequential monitoring boundaries were constructed. This enables one to determine the statistical inference concerning cumulative meta-analysis that has not yet reached the DARIS [39, 44]. Firm evidence for benefit or harm may be established if a trial sequential monitoring boundary (i.e. upper boundary of benefit or lower boundary of harm) is crossed before reaching the DARIS. In contrast, if a boundary is not surpassed, one may conclude that it is necessary to continue with further trials before a certain intervention effect can be detected or rejected. Firm evidence for lack of the postulated intervention effect can also be assessed with TSA. This occurs if the boundaries of futility are crossed [24, 25, 36, 39–48]. The TSA program is also able to calculate TSA-adjusted CIs, which we used instead of 95% CIs if the cumulative Z-curves did not reach the futility area or the DARIS. This gives a more correct estimation of the true CI, as the TSA-adjusted CI compared to the unadjusted naïve 95% CI adjusts for lack of information [46]. If the TSA could not be conducted because of too little information, we conducted a more lenient analysis by increasing the anticipated intervention effect (in these cases, the TSA-adjusted CI is overly optimistic). Statistical heterogeneity was assessed by visual inspection of forest plots and by calculating inconsistency (I^2^) for traditional meta-analyses and diversity (D^2^) for TSA [26, 44]. Sensitivity analyses and subgroup analyses were conducted to explore the reasons for substantial statistical heterogeneity [36]. We assessed the risk of publication bias in meta-analyses consisting of 10 trials or more with tests for funnel plot asymmetry. We assessed three primary outcomes and, hence, considered a P value of 0.025 or less as the threshold for statistical significance for the primary outcomes [36, 50]. We assessed four secondary outcomes and, hence, considered a P value of 0.020 or less as the threshold for statistical significance for the secondary outcomes [36, 50]. We used ‘best-worst case’ analyses and ‘worst-best case’ analyses to assess the potential impact of missing data (incomplete outcome data bias) [36]. We calculated Bayes factor to show if the meta-analysis results supported the null hypothesis or the anticipated intervention effect [36]. We used GRADE to assess the quality of the body of evidence [36, 51–53]. GRADE consist of five domains which are: bias risk of the trials; consistency of effect; imprecision; indirectness; and publication bias [36, 51–53].

## Results

### Study characteristics

**Fig 1:**
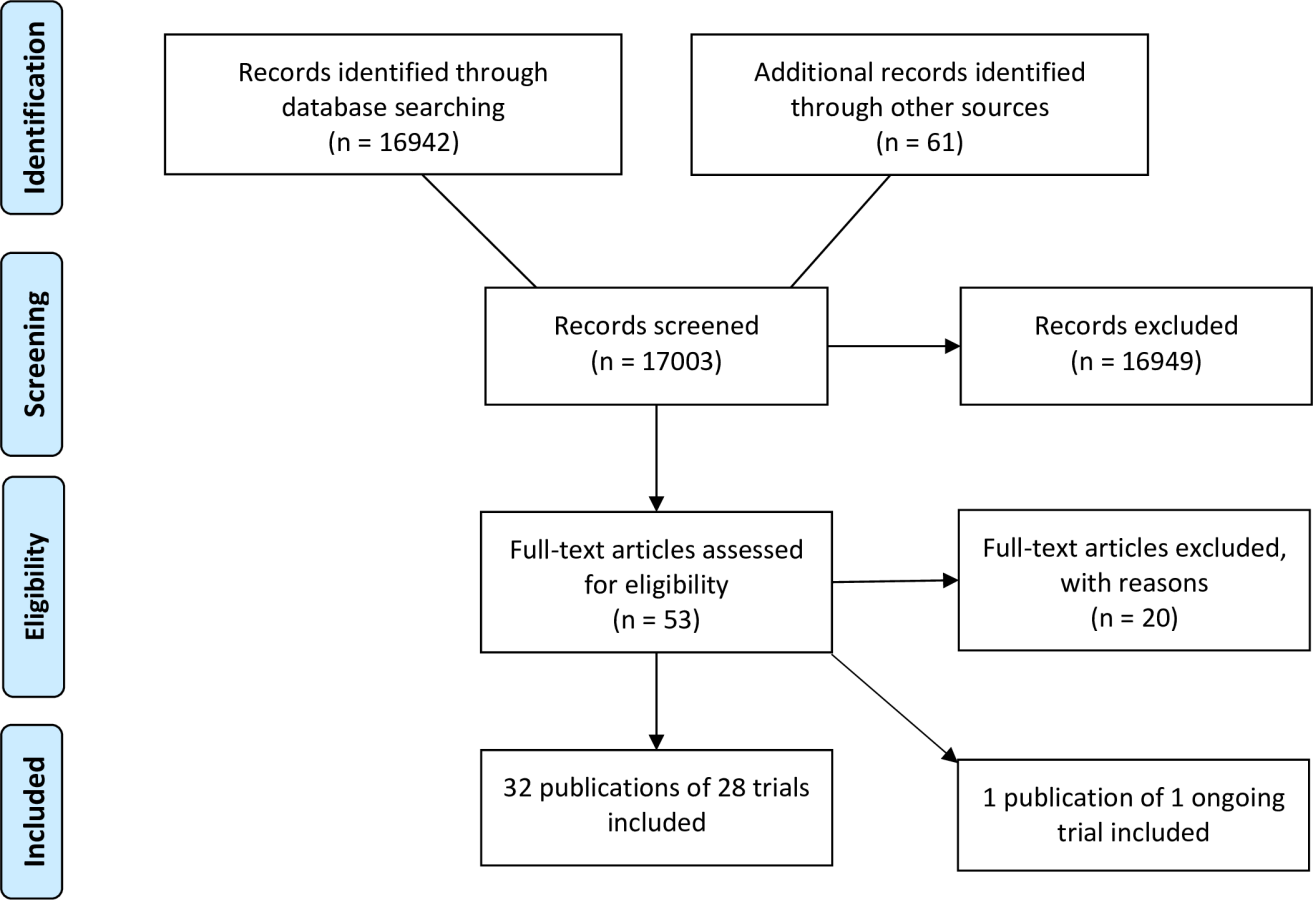
PRISMA flow diagram. We screened 17 003 records and included 32 publications of 28 trials in this systematic review.

Our literature search identified a total of 17 003 references. We were able to include 28 randomized clinical trials reported in 32 publications with 37 comparisons including a total of 2223 participants (Fig 1) [54–85].

All 28 trials were at high risk of bias (S1 Fig). All trials included participants with atrial fibrillation and two trials included both participants with atrial fibrillation and atrial flutter [72, 85]. The individual trials used various types of control interventions (see S2 Table): placebo (9/37 trial comparisons), amiodarone (9/37 trial comparisons), calcium antagonists (6/37 trial comparisons), beta blockers (5/37 trial comparisons), class Ic drugs (flecainide or propafenone) (5/37 trial comparisons), clonidine (1/37 trial comparisons), procainamide (1/37 trial comparisons), and no intervention (1/37 trial comparisons). 16 trials administered the interventions intravenously, 9 trials administered the interventions orally, while 3 trials administered the interventions both intravenously and orally (see S2 Table). 23 trials reported length of follow-up. 18 trials had a mean maximum follow-up of 18.2 hours (1 hour to 48 hours), while the remaining 5 trials ranged from 8 days to 24 weeks follow-up (see S2 Table). Hence, we had no trial with long-term follow-up. We have summarized the inclusion- and exclusion criteria for each included trial in S1 Table and other trial characteristics in S2 Table. Additionally, we have summarized the characteristics of excluded studies [86–105] and characteristics of ongoing studies [106] in S2 Table.

We have reported the baseline characteristics of the participants in Table 1. Importantly, several of the trials did not report all or any baseline characteristics of their included participants.

**Table 1:** Baseline characteristics of the participants.

	Trials providing information	Digoxin	No. of participant (digoxin)	Control	No. of participants (control)	P value
**Age–years (SD)**	19/28	63.7 (12.3)	717	64.8 (12)	913	0.89
**Male sex–no. (%)**	20/28	433 (59.2)	731	536 (57.9)	925	0.44
**Female sex–no. (%)**	20/28	298 (40.8)	731	389 (42.1)	925	0.75
**Weight–kg (SD)**	6/28	69.2 (12.2)	314	68.8 (12.4)	353	0.93
**Diabetes–no. (%)**	5/28	40 (15.2)	263	51 (16)	318	0.94
**Heart failure–no. (%)**	6/28	104 (30.1)	346	101 (30.2)	334	0.74
**Hypertension–no. (%)**	14/28	247 (40.1)	616	284 (35.7)	795	0.57
**Ischemic or valvular disease–no. (%)**	14/28	111 (19.1)	582	162 (22.7)	713	0.25
**Chronic obstructive pulmonary disorder–no. (%)**	5/28	27 (9.3)	291	29 (8.4)	345	0.70
**Thyroid dysfunction–no. (%)**	3/28	7 (3.4)	203	13 (4.3)	301	0.83
**Ejection fraction–% (SD)**	7/28	60 (11.9)	275	59.5 (14.4)	314	0.81
**Mean ventricular rate–bpm (SD)**	16/28	141.4 (22.1)	574	141.8 (21.4)	726	0.68

Table 1 legend: Several or more of the trials did not report all or any baseline characteristics of their included participants. Hence, the baseline characteristics does not include data from all included participants, but a smaller sample.

### Clinical outcomes

#### Safety outcomes

**Fig 2:**
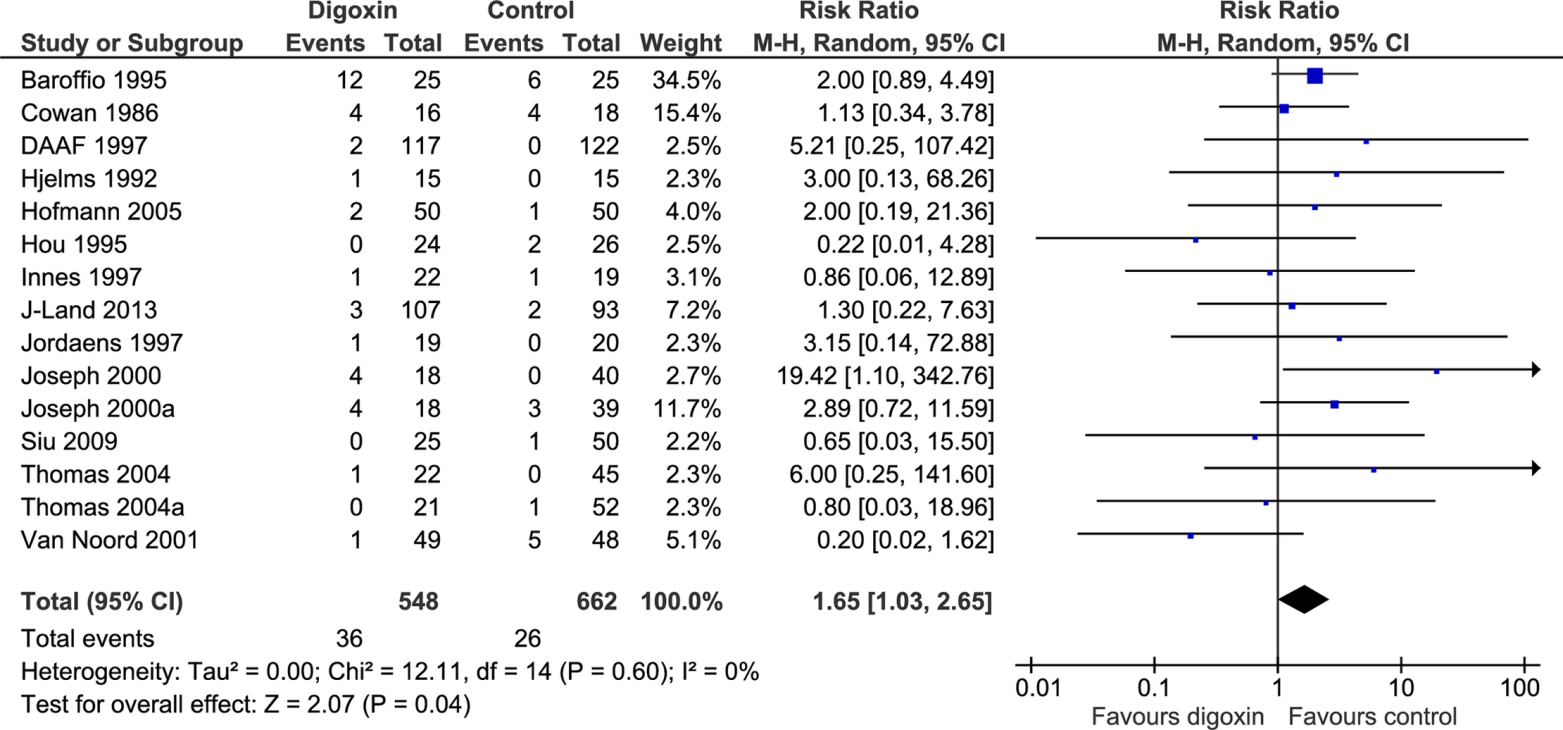
Forest plot of the meta-analysis on serious adverse events. Meta-analysis on serious adverse events suggested that digoxin might have a harmful effect.

When digoxin was compared with all control interventions in one analysis, our analyses on all-cause mortality (RR, 0.82; TSA-adjusted CI, 0.02 to 31.2; P = 0.67; I^2^ = 0%; 522 participants; 6 trials; very low quality of evidence; S2 and S3 Figs), heart failure (RR, 1.05; TSA-adjusted CI, 0.00 to 1141.8; P = 0.96; I^2^ = 51%; 462 participants; 4 trials; very low quality of evidence; S4 and S5 Figs), and stroke (RR, 2.27; TSA-adjusted CI, 0.00 to 7887.3; P = 0.42; I^2^ = 17%; 325 participants; 3 trials; very low quality of evidence; S6 and S7 Figs) showed no evidence of a difference between the digoxin versus the control group. However, meta-analysis on serious adverse events suggested that digoxin might have a harmful effect, but TSA showed that there was not enough information to confirm or reject a RRR of 22.5% (RR, 1.65; TSA-adjusted CI, 0.24 to 11.5; P = 0.04; I^2^ = 0%; 1210 participants; 13 trials; very low quality of evidence; Fig 2 and S8 Fig). The increased risk of a serious adverse event did not seem to be driven by a particular component of the composite outcome. Bayes factor was larger than 0.1 for all comparisons, showing no support for the alternative hypothesis that digoxin is better (Table 2). Incomplete outcome data bias alone had the potential to influence the results when assessing serious adverse events (S9 and S10 Figs) and stroke (S11 and S12 Figs) (Table 2), but not when assessing all-cause mortality (S13 and S14 Figs) and heart failure (S15 and S16 Figs) (Table 2). The planned subgroup analyses of all-cause mortality (S17–S20 Figs) and serious adverse events (S21–S24 Figs) showed no significant differences.

**Table 2:** Safety outcomes.

	Trials providing information	Participants	Risk ratio (RR)	Trial Sequential Analysis (TSA)-adjusted confidence intervals (CI)	P value	I^2^	Bayes factor	Best-worst case scenario (RR [95% CI])	Worst-best case scenario (RR [95% CI])
**All-cause mortality**	6	522	0.82	*0.02 to 31.2	0.67	0%	0.91	0.59 [0.19 to 1.84]	1.45 [0.58 to 3.64]
**Serious adverse events**	13	1210	1.65	*0.24 to 11.5	0.04	0%	5.15	1.27 [0.74 to 2.18]	2.10 [1.26 to 3.49]
**Heart failure**	4	462	1.05	*0.00 to 1141.8	0.96	51%	1.03	0.64 [0.10 to 4.21]	2.39 [0.45 to 12.7]
**Stroke**	3	325	2.27	*0.00 to 7887.3	0.42	17%	1.15	1.19 [0.05 to 31.4]	7.98 [1.81 to 35.3]

Table 2 legend: (*) means that we conducted a more lenient TSA than originally planned due to lack of information. Accordingly, our TSA-adjusted CIs are too narrow.

We have summarized the results of the safety outcomes in Table 2. Furthermore, we have summarized the specific types of serious adverse events in each trial in S3 Table.

#### Quality of life

Quality of life was assessed by different scales, including atrial fibrillation symptom frequency score (AF-SFS), atrial fibrillation symptom severity score (AF-SSS), SF-36 physical component score (SF-36 PCS), and SF-36 mental component score (SF-36 MCS). Our analyses showed no evidence of a difference between the digoxin versus the control group on AF-SFS (MD, 0.98 points; TSA-adjusted CI, -1.45 to 3.41; P = 0.43; I^2^ = 0%; 166 participants; 2 trials; very low quality of evidence; S25 and S26 Figs), AF-SSS (MD, -0.24 points; TSA-adjusted CI, -7.95 to 7.47; P = 0.88, I^2^ = 60%; 166 participants; 2 trials; very low quality of evidence; S27 and S28 Figs), SF-36 PCS (MD, 0.00 points; TSA-adjusted CI, -84.7 to 84.7; P = 1.00; 16 participants; 1 trial; very low quality of evidence; S29 and S30 Figs), and SF-36 MCS (MD, -5.00 points; TSA-adjusted CI, -94.8 to 84.8; P = 0.66; 16 participants; 1 trial; very low quality of evidence; S31 and S32 Figs). TSA of AF-SFS showed that the Z-curve crossed the boundary of futility indicating that we had sufficient information to reject a MD of 3.99 points (S26 Fig). Bayes factor was larger than 0.1 for all comparisons, showing no support for the alternative hypothesis that digoxin is better (Table 3). There was no incomplete outcome data. Subgroup analysis could not be conducted because of too few trials reporting quality of life.

**Table 3:** Quality of life.

	Trials providing information	Participants	Mean difference (points)	Trial Sequential Analysis (TSA)-adjusted confidence intervals (CI)	P value	I^2^	Bayes factor
**AF symptom frequency score**	2	166	0.98	-1.45 to 3.41	0.43	0%	13.93
**AF symptom severity score**	2	166	-0.24	-7.95 to 7.47	0.88	60%	6.47
**SF-36 PCS**	1	16	0.00	-84.7 to 84.7	1.00	-	1.65
**SF-36 MCS**	1	16	-5.00	-94.8 to 84.8	0.66	-	2.57

We have summarized the results of each quality of life scale in Table 3.

### Heart rate control and conversion to sinus rhythm

We will in the following paragraphs, separately, present the results on digoxin versus placebo, digoxin versus beta blockers, digoxin versus calcium antagonists, and digoxin versus amiodarone on heart rate control (Table 4 and S4 Table) and conversion to sinus rhythm (Table 5). Table 4 presents heart rate control in patients remaining in atrial fibrillation throughout the follow-up; S4 Table presents heart rate control in all patients regardless of the heart rhythm; and Table 5 presents conversion to sinus rhythm.

**Table 4:** Heart rate control in patients remaining in atrial fibrillation.

Outcome	Comparison	Trials providing information	Participants	Mean difference (bpm)	Trial Sequential Analysis (TSA)-adjusted confidence intervals (CI)	P value	I^2^	Bayes factor	Best-worst case scenario (MD [95% CI])	Worst-best case scenario (MD [95% CI])
**Heart rate control within 6 hours of treatment onset**	Digoxin vs. placebo	4	306	-12.0	-17.2 to -6.8	<0.00001	0%	4.14e^-5^	-	-
	Digoxin vs. beta blockers	2	90	20.7	14.2 to 27.2	<0.00001	0%	3.62e^13^	20.6 [17.2 to 23.9]	20.9 [17.5 to 24.3]
	Digoxin vs. calcium antagonists	1	35	21.0	-30.3 to 72.3	0.01	-	0.07	-	-
	Digoxin vs. amiodarone	4	196	14.7	-0.58 to 30.0	<0.00001	42%	31689	13.9 [7.0 to 20.8]	15.3 [9.9 to 20.7]
	Digoxin vs. class Ic drugs	2	56	9.77	-21.8 to 41.4	0.14	0%	93.9	-	-
**Heart rate control 6 to 24 hours after treatment onset**	Digoxin vs. placebo	1	123	-25.0	-37.9 to -12.1	<0.00001	-	1.88e^-6^	-	-
	Digoxin vs. beta blockers	2	52	11.7	-9.9 to 33.3	0.006	0%	0.036	11.4 [3.0 to 19.8]	13.1 [2.1 to 24.1]
	Digoxin vs. calcium antagonists	1	23	17.0	-63.6 to 97.6	0.09	-	0.63	-	-
	Digoxin vs. amiodarone	3	64	-2.03	-20.6 to 16.5	0.62	0%	2.76	-3.26 [-11.2 to 4.67]	-0.94 [-8.88 to 6.99]

**Table 5:** Conversion to sinus rhythm.

Outcome	Comparison	Trials providing information	Participants	Risk ratio (RR)	Trial Sequential Analysis (TSA)-adjusted confidence intervals (CI)	P value	I^2^	Bayes factor	Best-worst case scenario (MD [95% CI])	Worst-best case scenario (MD [95% CI])
**Conversion to sinus rhythm within 6 hours of treatment onset**	Digoxin vs. placebo	4	453	1.39	0.33 to 5.91	0.07	0%	7.58	-	-
	Digoxin vs. beta blockers	3	323	0.77	*0.07 to 8.53	0.39	0%	0.72	0.54 [0.25 to 1.18]	1.20 [0.53 to 2.73]
	Digoxin vs. calcium antagonists	4	122	0.82	*0.01 to 59.3	0.72	46%	0.94	0.80 [0.26 to 2.42]	0.89 [0.36 to 2.18]
	Digoxin vs. amiodarone	6	344	0.54	0.13 to 2.21	0.0004	0%	0.061	0.53 [0.37 to 0.74]	0.58 [0.40 to 0.84]
	Digoxin vs. class Ic drugs	5	307	0.48	0.12 to 1.93	<0.0001	0%	0.03	-	-
	Digoxin vs. clonidine	1	19	0.86	*0.00 to 280.3	0.83	-	0.98	-	-
**Conversion to sinus rhythm 6 to 24 hours after treatment onset**	Digoxin vs. placebo	6	484	1.15	0.59 to 2.27	0.09	3%	178	-	-
	Digoxin vs. beta blockers	2	125	0.83	*0.08 to 8.71	0.53	61%	0.82	0.79 [0.40 to 1.56]	0.86 [0.53 to 1.39]
	Digoxin vs. calcium antagonists	3	156	0.82	*0.16 to 4.07	0.32	40%	0.63	0.78 [0.48 to 1.26]	0.84 [0.60 to 1.17]
	Digoxin vs. amiodarone	6	319	0.85	0.47 to 1.54	0.03	0%	0.096	0.84 [0.73 to 0.97]	0.86 [0.74 to 0.99]
	Digoxin vs. class Ic drugs	1	53	0.85	*0.21 to 3.39	0.34	-	0.65	-	-

Table 5 legend: (*) means that we conducted a more lenient TSA than originally planned due to lack of information. Accordingly, our TSA-adjusted CIs are too narrow.

#### Digoxin versus placebo

**Fig 3:**
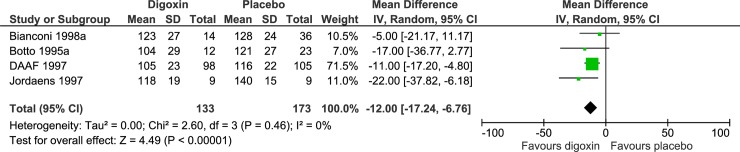
Forest plot of the meta-analysis of digoxin versus placebo on heart rate control within 6 hours of treatment onset. Meta-analysis showed firm evidence of digoxin being superior compared with placebo.

**Fig 4:**
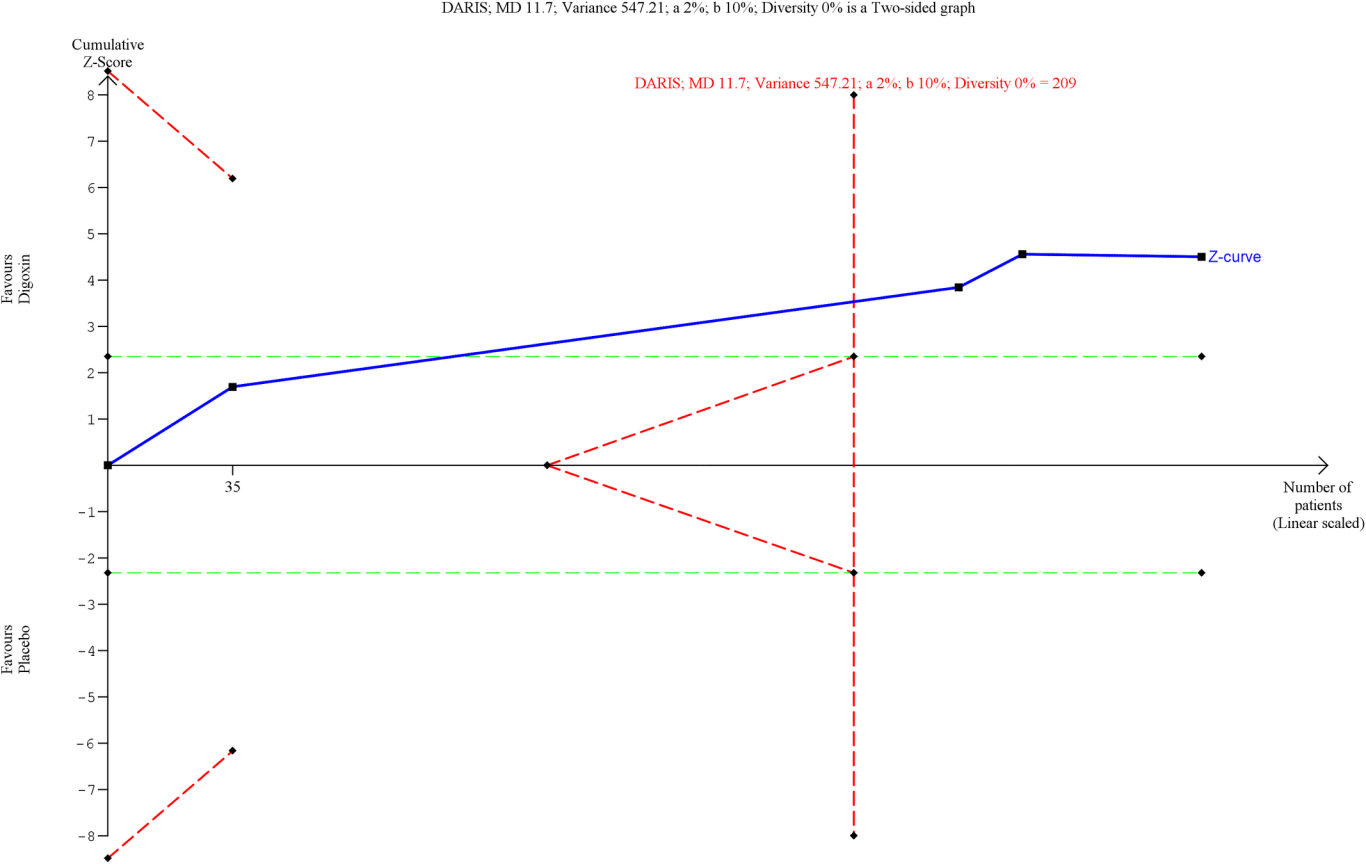
Trial sequential analysis of digoxin versus placebo on heart rate control within 6 hours of treatment onset. Trial Sequential Analysis (TSA) of digoxin versus placebo on heart rate control within 6 hours of treatment onset showed that the Z-curve (the blue line) crossed the upper trial sequential monitoring boundary for benefit (the upper red line). Hence, we have enough information to confirm that digoxin is superior compared with placebo in controlling the heart rate within 6 hours of treatment onset.

Our analyses showed firm evidence of digoxin being superior compared with placebo in controlling the heart rate both within 6 hours of treatment onset (MD, -12.0 beats per minute (bpm); TSA-adjusted CI, -17.2 to -6.76; P < 0.00001; I^2^ = 0%; 306 participants; 4 trials; low quality of evidence; Figs 3 and 4) and 6 to 24 hours after treatment onset (MD, -25.0 bpm; TSA-adjusted CI, -37.9 to -12.1; P < 0.00001; 123 participants; 1 trial; low quality of evidence; S33 and S34 Figs). Our analyses showed that digoxin was more effective in controlling the heart rate 6 to 24 hours after treatment onset than within 6 hours of treatment onset.

Our analyses showed no evidence of a difference on the probability of converting the atrial fibrillation to sinus rhythm both within 6 hours of treatment onset (RR, 1.39; TSA-adjusted CI, 0.33 to 5.91; P = 0.07; I^2^ = 0%; 453 participants; 4 trials; very low quality of evidence; S35 and S36 Figs) and 6 to 24 hours after treatment onset (RR, 1.15; TSA-adjusted CI, 0.59 to 2.27; P = 0.09; I^2^ = 3%; 484 participants; 6 trials; very low quality of evidence; S37 and S38 Figs).

There was no incomplete outcome data for both heart rate control and conversion to sinus rhythm. Meta-analysis on heart rate control in all patients regardless of type of rhythm supported these findings (S4 Table).

#### Digoxin versus beta blockers

**Fig 5:**
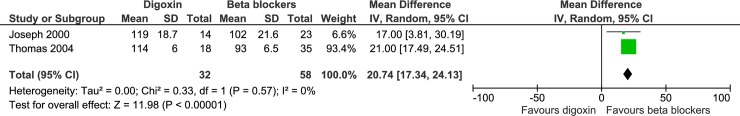
Forest plot of the meta-analysis of digoxin versus beta blockers on heart rate control within 6 hours of treatment onset. Meta-analysis showed firm evidence of digoxin being inferior compared with beta blockers.

**Fig 6:**
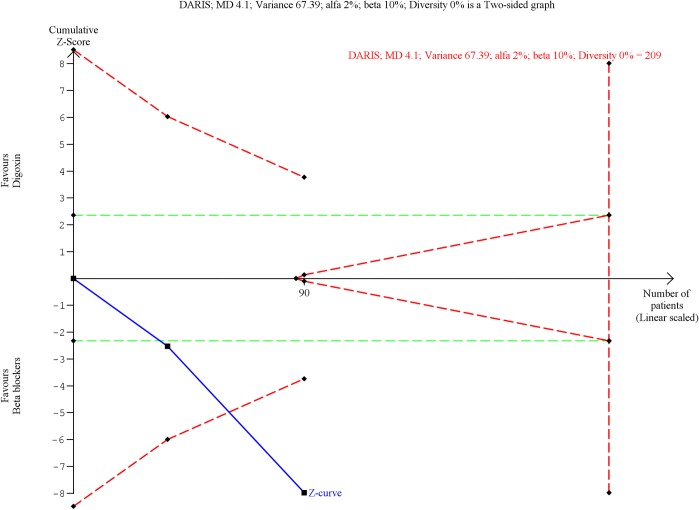
Trial sequential analysis of digoxin versus beta blockers on heart rate control within 6 hours of treatment onset. Trial Sequential Analysis (TSA) of digoxin versus beta blockers on heart rate control within 6 hours of treatment onset showed that the Z-curve (the blue line) crossed the lower trial sequential monitoring boundary for harm (the lower red line). Hence, we have enough information to confirm that digoxin is inferior compared with beta blockers in controlling the heart rate within 6 hours of treatment onset.

Our analyses showed firm evidence of digoxin being inferior compared with beta blockers in controlling the heart rate within 6 hours of treatment onset (MD, 20.7 bpm; TSA-adjusted CI, 14.2 to 27.2; P < 0.00001; I^2^ = 0%; 90 participants; 2 trials; low quality of evidence; Figs 5 and 6). Meta-analysis 6 to 24 hours after treatment onset showed evidence of a difference, but TSA showed that there was not enough information to confirm or reject a MD of 7.73 bpm (MD, 11.7 bpm; TSA-adjusted CI, -9.86 to 33.3; P = 0.006; I^2^ = 0%; 52 participants; 2 trials; very low quality of evidence; S39 and S40 Figs). Our analyses showed that digoxin was more effective in controlling the heart rate 6 to 24 hours after treatment onset than within 6 hours of treatment onset.

Our analyses showed no evidence of a difference on the probability of converting the atrial fibrillation to sinus rhythm both within 6 hours of treatment onset (RR, 0.77; TSA-adjusted CI, 0.07 to 8.53; P = 0.39; I^2^ = 0%; 323 participants; 3 trials; very low quality of evidence; S41 and S42 Figs) and 6 to 24 hours after treatment onset (RR, 0.83; TSA-adjusted CI, 0.08 to 8.71; P = 0.53; I^2^ = 61%; 125 participants; 2 trials; very low quality of evidence; S43 and S44 Figs).

Incomplete outcome data bias alone did not have the potential to influence the results for both heart rate control (S45–S48 Figs) and conversion to sinus rhythm (S49–S52 Figs) (Tables 4 and 5). Meta-analysis on heart rate control in all patients regardless of type of rhythm supported these findings (S4 Table).

#### Digoxin versus calcium antagonists

Meta-analysis showed that digoxin was inferior compared with calcium antagonists in controlling the heart rate within 6 hours of treatment onset, but TSA showed that there was not enough information to confirm or reject a MD of 12.3 bpm (MD, 21.0 bpm; TSA-adjusted CI, -30.3 to 72.3; P = 0.01; 35 participants; 1 trial; very low quality of evidence; S53 and S54 Figs). Our analyses showed no evidence of a difference on heart rate control 6 to 24 hours after treatment onset (MD, 17.0 bpm; TSA-adjusted CI, -63.6 to 97.6; P = 0.09; 23 participants; 1 trial; very low quality of evidence; S55 and S56 Figs), conversion to sinus rhythm within 6 hours of treatment onset (RR, 0.82; TSA-adjusted CI, 0.01 to 59.3; P = 0.72; I^2^ = 46%; 122 participants; 4 trials; very low quality of evidence; S57 and S58 Figs), and conversion to sinus rhythm 6 to 24 hours after treatment onset (RR, 0.82; TSA-adjusted CI, 0.16 to 4.07; P = 0.32; I^2^ = 40%; 156 participants; 3 trials; very low quality of evidence; S59 and S60 Figs). There was no incomplete outcome data for heart rate control. For conversion to sinus rhythm, incomplete outcome data bias alone did not have the potential to influence the results (S61–S64 Figs) (Table 5). Meta-analysis on heart rate control in all patients regardless of type of rhythm supported these findings (S4 Table). Our analyses showed that digoxin was more effective in controlling the heart rate 6 to 24 hours after treatment onset than within 6 hours of treatment onset.

#### Digoxin versus amiodarone

Meta-analysis showed that digoxin was inferior compared with amiodarone in controlling the heart rate within 6 hours of treatment onset, but TSA showed that there was not enough information to confirm or reject a MD of 5.71 bpm (MD, 14.7 bpm; TSA-adjusted CI, -0.58 to 30.0; P < 0.00001; I^2^ = 42%; 196 participants; 4 trials; very low quality of evidence; S65 and S66 Figs). Meta-analysis showed that digoxin compared with amiodarone reduced the probability of converting the atrial fibrillation to sinus rhythm within 6 hours of treatment onset, but TSA showed that there was not enough information to confirm or reject a RRR of 15% (RR, 0.54; TSA-adjusted CI, 0.13 to 2.21; P = 0.0004; I^2^ = 0%; 344 participants; 6 trials; very low quality of evidence; S67 and S68 Figs). Our analyses showed no evidence of a difference on heart rate control 6 to 24 hours after treatment onset (MD, -2.03 bpm; TSA-adjusted CI, -20.6 to 16.5; P = 0.62; I^2^ = 0%; 64 participants; 3 trials; very low quality of evidence; S69 and S70 Figs) and conversion to sinus rhythm 6 to 24 hours after treatment onset (RR, 0.85; TSA-adjusted CI, 0.47 to 1.54; P = 0.03; I^2^ = 0%; 319 participants; 6 trials; very low quality of evidence; S71 and S72 Figs). When assessing heart rate control, incomplete outcome data bias alone did not have the potential to influence the results (S73–S76 Figs) (Table 4). When assessing conversion to sinus rhythm, incomplete outcome data bias alone had the potential to influence the results when assessing the findings 6 to 24 hours after treatment onset (S77 and S78 Figs) (Table 5), but not when assessing the findings within 6 hours of treatment onset (S79 and S80 Figs) (Table 5). Meta-analysis on heart rate control in all patients regardless of type of rhythm supported these findings (S4 Table). Our analyses showed that digoxin was more effective in controlling the heart rate 6 to 24 hours after treatment onset than within 6 hours of treatment onset.

### Summary of findings

We have assessed and summarized the quality of the evidence of our main results in the summary of findings table (Table 6).

**Table 6:** Summary of findings table.

Outcomes	Anticipated absolute effects		Relative effect (Trial Sequential Analysis-adjusted confidence interval)	№ of participants (trials)	Quality of the evidence (GRADE)	Comments
	Risk with digoxin	Risk with placebo, no intervention, or other medical intervention				
All-cause mortality	26 per 1000	39 per 1000	0.82 (0.02 to 31.2)	522 (6 trials)	⊕⊝⊝⊝—Very low quality of evidence caused by imprecision (-2)^1^, and risk of bias (-1)^3^.	Trial Sequential Analysis showed that there was not enough information to confirm or reject a RRR of 15%. Moreover, the meta-analysis showed wide CI. All trials had high risk of bias, mostly because of ‘for-profit bias’ and ‘incomplete outcome data bias’.
Serious adverse events	66 per 1000	39 per 1000	1.65 (0.24 to 11.5)	1210 (13 trials)	⊕⊝⊝⊝—Very low quality of evidence caused by imprecision (-1)^2^ and risk of bias (-2)^6^.	Trial Sequential Analysis showed that there was not enough information to confirm or reject a RRR of 15%. Moreover, the meta-analysis showed wide CI. All trials had high risk of bias, mostly because of ‘for-profit bias’, ‘incomplete outcome data bias’, and ‘incomplete blinding of participants and personnel’.
Quality of life	Quality of life showed no evidence of a difference on AF symptom frequency score (MD 0.98, TSA-adjusted CI -1.45 to 3.41).			166 (2 trials)	⊕⊝⊝⊝—Very low quality of evidence caused by imprecision (-2)^1^ and risk of bias (-1)^3^.	Trial Sequential Analysis for AF symptom frequency score showed that the z-curve crossed the boundary of futility, and Trial Sequential Analysis for AF symptom severity score showed that there was not enough information to confirm or reject out anticipated intervention effect. Moreover, both meta-analyses showed very wide CI. Both trials had high risk of bias, mostly because of ‘incomplete blinding of participants and personnel’. Meta-analysis of AF-symptom severity score had moderate levels of heterogeneity.
	Quality of life showed no evidence of a difference on AF symptom severity score (MD -0.24, TSA-adjusted CI -7.95 to 7.47).			166 (2 trials)	⊕⊝⊝⊝—Very low quality of evidence caused by imprecision (-2)^1^, risk of bias (-1)^3^, and inconsistency (-1)^4^.	
Heart failure	28 per 1000	33 per 1000	1.05 (0.00 to 1141.8)	462 (4 trials)	⊕⊝⊝⊝—Very low quality of evidence caused by imprecision (-2)^1^, risk of bias (-1)^3^, and inconsistency (-1)^4^.	Trial Sequential Analysis showed that there was not enough information to confirm or reject a RRR of 15%. Moreover, the meta-analysis showed very wide CI. All trials had high risk of bias, mostly because of ‘incomplete blinding of participants and personnel’ and ‘incomplete outcome data bias’. Meta-analysis had moderate levels of heterogeneity.
Stroke	14 per 1000	6 per 1000	2.27 (0.00 to 7887.3)	325 (3 trials)	⊕⊝⊝⊝—Very low quality of evidence caused by imprecision (-2)^1^, risk of bias (-1)^3^.	Trial Sequential Analysis showed that there was not enough information to confirm or reject a RRR of 15%. Moreover, the meta-analysis showed very wide CI. All trials had high risk of bias, mostly because ‘incomplete outcome data bias’.
Heart rate control (within 6 hours of treatment onset)	Meta-analyses showed that digoxin was superior compared with placebo (MD -12.0, TSA-adjusted CI -17.2 to -6.76) in controlling the heart rate within 6 hours of treatment onset.			306 (4 trials)	⊕⊕⊝⊝—Low quality of evidence caused by risk of bias (-1)^3^ and indirectness (-1)^5^.	Trial Sequential Analysis showed that we had enough information for digoxin versus placebo, and digoxin versus beta blockers. However, we did not have enough information for digoxin versus calcium antagonists, and digoxin versus amiodarone. All trials had high risk of bias, mostly because of ‘incomplete blinding of participants and personnel’ and ‘for-profit bias’. Heart rate control is a surrogate outcome. Hence, it has serious risk of indirectness. Meta-analysis for digoxin versus amiodarone showed moderate levels of heterogeneity.
	Meta-analyses showed that digoxin was inferior compared with beta blockers (MD 20.7, TSA-adjusted CI 14.2 to 27.1) in controlling the heart rate within 6 hours of treatment onset.			90 (2 trials)	⊕⊕⊝⊝—Low quality of evidence caused by risk of bias (-1)^3^ and indirectness (-1)^5^.	
	Meta-analyses showed that digoxin was inferior compared with calcium antagonists (MD 21.0, TSA-adjusted CI -30.3 to 72.3) in controlling the heart rate within 6 hours of treatment onset.			35 (1 trial)	⊕⊝⊝⊝—Very low quality of evidence caused by imprecision (-1)^1^, risk of bias (-1)^3^, and indirectness (-1)^5^.	
	Meta-analyses showed that digoxin was inferior compared with amiodarone (MD 14.7, TSA-adjusted CI -0.58 to 30.0) in controlling the heart rate within 6 hours of treatment onset.			196 (4 trials)	⊕⊝⊝⊝—Very low quality of evidence caused by imprecision (-1)^2^, risk of bias (-1)^3^, inconsistency (-1)^4^, and indirectness (-1)^5^.	
Heart rate control (6 to 24 hours after treatment onset)	Meta-analyses showed that digoxin was superior compared with placebo (MD -25.0, TSA-adjusted CI -37.9 to -12.1) in controlling the heart rate 6 to 24 hours after treatment onset.			123 (1 trial)	⊕⊕⊝⊝—Low quality of evidence for digoxin versus placebo caused by risk of bias (-1)^3^ and indirectness (-1)^5^.	Trial Sequential Analysis showed that we had enough information for digoxin versus placebo. However, we did not have enough information for digoxin versus beta blockers, digoxin versus amiodarone, and digoxin versus calcium antagonists. Moreover, the meta-analysis of digoxin versus amiodarone, and digoxin versus calcium antagonists showed wide CI. All trials had high risk of bias, mostly because of ‘incomplete blinding of participants and personnel’ and ‘for-profit bias’. Heart rate control is a surrogate outcome. Hence, it has serious risk of indirectness.
	Meta-analyses showed that digoxin was inferior compared with beta blockers (MD 11.7, TSA-adjusted CI -9.86 to 33.3) in controlling the heart rate 6 to 24 hours after treatment onset.			52 (2 trials)	⊕⊝⊝⊝—Very low quality of evidence for digoxin versus beta blockers caused by imprecision (-1)^2^, risk of bias (-1)^3^, and indirectness (-1)^5^.	
	Meta-analyses showed that digoxin was similar compared with calcium antagonists (MD 17.0, TSA-adjusted CI -63.6 to 97.6) in controlling the heart rate 6 to 24 hours after treatment onset.			23 (1 trials)	⊕⊝⊝⊝—Very low quality of evidence for digoxin versus calcium antagonists caused by imprecision (-1)^2^, risk of bias (-1)^3^, and indirectness (-1)^5^.	
	Meta-analyses showed that digoxin was similar compared with amiodarone (MD -2.03, TSA-adjusted CI -20.6 to 16.5) in controlling the heart rate 6 to 24 hours after treatment onset.			64 (3 trials)	⊕⊝⊝⊝—Very low quality of evidence for digoxin versus amiodarone caused by imprecision (-2)^1^, risk of bias (-1)^3^, and indirectness (-1)^5^.	
Conversion to sinus rhythm (within 6 hours of treatment onset)	290 per 1000 (digoxin versus placebo)	210 per 1000 (digoxin versus placebo)	1.39 (0.33 to 5.91) (digoxin versus placebo)	453 (4 trials) for digoxin versus placebo	⊕⊝⊝⊝—Very low quality of evidence for digoxin versus placebo caused by imprecision (-1)^2^, risk of bias (-2)^6^, and indirectness (-1)^5^.	Trial Sequential Analysis showed that there was not enough information to confirm or reject a RRR of 15% for each comparison. Moreover, the meta-analyses of digoxin versus calcium antagonists showed wide CI. All trials had high risk of bias, mostly because of ‘incomplete blinding of participants and personnel’, ‘incomplete outcome data bias’, and ‘for-profit bias’. Conversion to sinus rhythm is a surrogate outcome. Hence, it has serious risk of indirectness. Meta-analysis for digoxin versus calcium antagonists showed moderate levels of heterogeneity.
	82 per 1000 (digoxin versus beta blockers)	170 per 1000 (digoxin versus beta blockers)	0.77 (0.07 to 8.53) (digoxin versus beta blockers)	323 (3 trials) for digoxin versus beta blockers	⊕⊝⊝⊝—Very low quality of evidence for digoxin versus beta blockers caused by imprecision (-1)^2^, risk of bias (-2)^6^, and indirectness (-1)^5^.	
	203 per 1000 (digoxin versus calcium antagonists)	286 per 1000 (digoxin versus calcium antagonists)	0.82 (0.01 to 59.3) (digoxin versus calcium antagonists)	122 (4 trials) for digoxin versus calcium antagonists	⊕⊝⊝⊝—Very low quality of evidence for digoxin versus calcium antagonists caused by imprecision (-2)^1^, risk of bias (-2)^6^, inconsistency (-1)^4^, and indirectness (-1)^5^.	
	222 per 1000 (digoxin versus amiodarone)	400 per 1000 (digoxin versus amiodarone)	0.54 (0.13 to 2.21) (digoxin versus amiodarone)	344 (6 trials) for digoxin versus amiodarone	⊕⊝⊝⊝—Very low quality of evidence for digoxin versus amiodarone caused by imprecision (-1)^1^, risk of bias (-1)^3^, and indirectness (-1)^5^.	
Conversion to sinus rhythm (6 to 24 hours after treatment onset)	561 per 1000 (digoxin versus placebo)	500 per 1000 (digoxin versus placebo)	1.15 (0.59 to 2.27) (digoxin versus placebo)	484 (6 trials) for digoxin versus placebo	⊕⊝⊝⊝—Very low quality of evidence for digoxin versus placebo caused by imprecision (-1)^2^, risk of bias (-1)^3^, and indirectness (-1)^5^.	Trial Sequential Analysis showed that there was not enough information to confirm or reject a RRR of 15% for each comparison. All trials had high risk of bias, mostly because of ‘incomplete blinding of participants and personnel’, ‘incomplete outcome data bias’, and ‘for-profit bias’. Conversion to sinus rhythm is a surrogate outcome. Hence, it has serious risk of indirectness. Meta-analysis for digoxin versus beta blockers and digoxin versus calcium antagonists showed moderate levels of heterogeneity.
	500 per 1000 (digoxin versus beta blockers)	612 per 1000 (digoxin versus beta blockers)	0.83 (0.08 to 8.71) (digoxin versus beta blockers)	125 (2 trials) for digoxin versus beta blockers	⊕⊝⊝⊝—Very low quality of evidence for digoxin versus beta blockers caused by imprecision (-1)^2^, risk of bias (-2)^6^, inconsistency (-1)^4^, and indirectness (-1)^5^.	
	478 per 1000 (digoxin versus calcium antagonists)	506 per 1000 (digoxin versus calcium antagonists)	0.82 (0.16 to 4.07) (digoxin versus calcium antagonists)	156 (3 trials) for digoxin versus calcium antagonists	⊕⊝⊝⊝—Very low quality of evidence for digoxin versus calcium antagonists caused by imprecision (-1)^2^, risk of bias (-2)^6^, inconsistency (-1)^4^, and indirectness (-1)^5^.	
	563 per 1000 (digoxin versus amiodarone)	625 per 1000 (digoxin versus amiodarone)	0.85 (0.21 to 3.39) (digoxin versus amiodarone)	319 (6 trials) for digoxin versus amiodarone	⊕⊝⊝⊝—Very low quality of evidence for digoxin versus amiodarone caused by imprecision (-1)^1^, risk of bias (-2)^6^, and indirectness (-1)^5^.	

## Discussion

We were able to include 28 randomized clinical trials reported in 32 publications with 37 comparisons including a total of 2223 participants. All trials and outcome results were at high risk of bias and the quality of the evidence according to GRADE was ‘low’ to ‘very low’ (see Summary of Findings table (Table 6)). None of the included trials had long-term follow-up.

### Statement of principal findings

When digoxin was compared with all control interventions in one analysis, our analyses showed no evidence of a difference on all-cause mortality, quality of life, stroke, and heart failure. Meta-analysis on serious adverse events suggested that digoxin might have a harmful effect, but TSA showed that there was not enough information to confirm or reject a RRR of 22.5%. When we deal with harms, it should be considered if we need the same statistical backing as if we were dealing with benefits [107]. Our analyses on the effects of heart rate control showed firm evidence of digoxin being superior compared with placebo both within 6 hours of treatment onset and 6 to 24 hours after treatment onset, and inferior compared with beta blockers within 6 hours of treatment onset. Meta-analyses on heart rate control within 6 hours of treatment onset showed that digoxin was inferior compared with calcium antagonists and with amiodarone, and meta-analysis on conversion to sinus rhythm within 6 hours of treatment onset showed that digoxin compared with amiodarone reduced the probability of converting atrial fibrillation to sinus rhythm, but in all three comparisons TSAs showed that there was not enough information to confirm or reject the anticipated intervention effects. Overall, our analyses showed that digoxin was more effective in controlling the heart rate 6 to 24 hours after treatment onset than within 6 hours of treatment onset.

### Strengths and limitations of this systematic review

Our review has several strengths. We are the first to conduct a systematic review with meta-analysis of randomized clinical trials comparing digoxin versus placebo, no intervention, or other medical interventions for atrial fibrillation and atrial flutter [24, 25]. We followed our protocol which was registered prior to the systematic literature search [24, 25]. Data were double-extracted by independent authors minimizing the risk of inaccurate data-extraction, and we assessed the risk of bias in all trials according to Cochrane [26] and Lundh et al. [27]. We used GRADE to assess the quality of the evidence [51–53], TSA to control the risks of random errors [24, 25, 36, 39–48], the eight-step assessment suggested by Jakobsen et al. to assess if the thresholds for significance were crossed [36], and sensitivity analyses (best-worst and worst-best) to test the potential impact of incomplete outcome data bias [36]. Hence, this systematic review considered both risks of random errors and risks of systematic errors, which adds further robustness to our results and conclusions.

Our review also has several limitations. All trials were at high risk of bias and especially the risk of incomplete blinding of participants and personnel and for-profit bias might bias our review results. Our assessment of especially publication bias was also uncertain, as a relatively low number of trials were included. Furthermore, some of the performed meta-analyses had considerable statistical heterogeneity. Hence, publication bias and heterogeneity might further bias our results. Large meta-epidemiological studies have shown that trials at high risk of bias tend to overestimate benefits and underestimate harms of experimental interventions [27–33]. Furthermore, the trials that were included in our review generally randomized a small number of participants and had short follow-up periods which limits the usefulness of the results (see S2 Table). When assessing the overall quality of the available evidence, GRADE assessment showed that the quality of the evidence was ‘low’ to ‘very low’, mostly due to imprecision and risk of bias (see Summary of Findings Table (Table 6)).

The potential relevance of heart rate control as a surrogate outcome is questionable. Heart rate reduction alone is believed to reduce the risk for cardiovascular events and death, and increase the quality of life, but its clinical relevance has not yet been fully established for patients with atrial fibrillation and atrial flutter [108, 109]. Moreover, the amount of heart rate reduction needed to sufficiently limit symptoms and reduce the risk of morbidity and mortality has also not been established. Beta blockers and calcium antagonists might reduce the heart rate more effectively than digoxin, but the clinical benefit of this is not known. Interestingly, a randomized clinical trial, RACE II, compared lenient rate control (<110 bpm at rest) with strict rate control (<80 bpm at rest) and showed that lenient rate control was as effective as strict rate control, easier to achieve, and required fewer outpatient visits and examinations [110]. Another limitation of our present review is the use of a composite outcome such as serious adverse events. A potential limitation when using composite outcomes is that each component of a composite outcome (in this case serious adverse events) will not necessarily have similar degrees of severity and will not be affected similarly by the interventions [22]. ‘True’ differences in severity between compared groups might therefore not be reflected in review results when using composite outcomes [22]. We believe that the clinical relevance of the outcome ‘serious adverse events’ and the resulting increased statistical power when using serious adverse events as an outcome, justifies the use of serious adverse events as a primary outcome, but the interpretative limitations ought to be considered.

### Strengths and limitations in relation to other systematic reviews and observational studies

We have identified one systematic review of randomized clinical trials assessing the effect of digoxin versus other medical interventions for atrial fibrillation and atrial flutter by Al-Khatib et al. [111]. They included nine trials randomizing 804 participants and showed no difference between digoxin and other medical interventions when assessing efficacy on ventricular rate control and safety. Our present review is the first systematic review of randomized clinical trials showing that digoxin is superior compared with placebo, but inferior compared with beta blockers in controlling the heart rate acutely.

We have identified four systematic reviews of observational studies assessing the effect of digoxin for atrial fibrillation and atrial flutter [17–20]. The four systematic reviews compared digoxin to no digoxin and showed an increased risk of all-cause mortality in the digoxin group [17–20]. This is contrary to our results, as we showed no evidence of a difference between digoxin and the control group based on current randomized evidence, which generally is more dependable [22].

### Comparison to current guidelines

Current guidelines recommend that beta blockers and calcium antagonists should be preferred over digoxin for acute rate control in patients with normal ejection fraction [12, 112, 113]. Our results confirm with firm evidence that beta blockers versus digoxin more effectively reduced the heart rate acutely. Calcium antagonists versus digoxin also seemed to more effectively reduce the heart rate acutely, but firm evidence was not available.

For long-term heart rate control, guidelines recommend that the choice of rate control drug should be made individually for each patient [12, 112, 113]. However, all three guidelines consider digoxin to be a second-line drug compared to beta blockers and calcium antagonists [12, 112, 113]. We could not find any long-term follow-up trials comparing the effects of digoxin with another rate control drug in patients with atrial fibrillation or atrial flutter.

### The possible contribution of ongoing trials

We identified one ongoing trial (see S2 Table) that might contribute to the current evidence on digoxin versus bisoprolol (a beta blocker) for atrial fibrillation and atrial flutter [106]. This ongoing trial will contribute to the evidence on, e.g., cardiovascular outcomes, hospitalization, quality of life, left ventricular ejection fraction, and heart rate control. All ongoing and future randomized clinical trials should be conducted at low risk of systematic error (bias) and low risk of random errors (play of chance), and ought to be designed and reported according to the SPIRIT and CONSORT guidelines [114, 115].

## Conclusions

The clinical effects of digoxin on all-cause mortality, serious adverse events, quality of life, heart failure, and stroke are unclear based on current evidence. Digoxin may increase the risk of a serious adverse event, but no firm evidence was available. Our systematic review could neither confirm nor reject the findings from recent systematic reviews of observational studies showing that digoxin compared to no digoxin increased the risk of death. The increased risk of death found in observational studies might be caused by selection, prescription biases, or due to a longer follow-up [21, 22]. Based on current randomized evidence, digoxin is superior compared with placebo, but inferior compared with beta blockers in controlling the heart rate acutely. Digoxin also seems to be inferior compared with calcium antagonists and with amiodarone in controlling the heart rate acutely, but firm evidence is not available. The long-term effect of digoxin is unclear, as no trials reported long-term follow-up. More randomized clinical trials assessing the clinical effects of digoxin are needed, especially considering that our results indicate that digoxin might increase the risk of serious adverse events.

## Differences between the protocol and the review

- We changed our assessment time-point for heart rate control and conversion to sinus rhythm. According to the protocol, we would use the trial results reported within 48 hours after treatment onset. However, in this review, we had two assessment time-points. We both performed meta-analyses within 6 hours of treatment onset and meta-analyses 6 to 24 hours after treatment onset. We made this change so we were able to investigate the effect of digoxin on heart rate control and conversion to sinus rhythm in both the acute and subacute phase.
- We performed TSAs on all meta-analysis results. If the TSA could not be conducted because of too little information, we conducted a more lenient TSA by increasing the anticipated intervention effect. In the protocol, we did not mention that we would perform these analyses.

## Supporting information

S1 Text PRISMA Checklist. (DOCX)

S2 Text Prepublished protocol for this systematic review. (PDF)

S3 Text Search strategy for MEDLINE (OVIDSP; 1946 to October 2016). (PDF)

S1 Fig Bias risk assessment of each included trial. (TIF)

S2 Fig Forest plot of all-cause mortality. (TIF)

S3 Fig Trial Sequential Analysis of all-cause mortality. (TIF)

S4 Fig Forest plot of heart failure. (TIF)

S5 Fig Trial sequential analysis of heart failure. (TIF)

S6 Fig Forest plot of stroke. (TIF)

S7 Fig Trial sequential analysis of stroke. (TIF)

S8 Fig Trial sequential analysis of serious adverse events. (TIF)

S9 Fig Forest plot of ‘best-worst case’ scenario for serious adverse events. (TIF)

S10 Fig Forest plot of ‘worst-best case’ scenario for serious adverse events. (TIF)

S11 Fig Forest plot of ‘best-worst case’ scenario for stroke. (TIF)

S12 Fig Forest plot of ‘worst-best case’ scenario for stroke. (TIF)

S13 Fig Forest plot of ‘best-worst case’ scenario for all-cause mortality. (TIF)

S14 Fig Forest plot of ‘worst-best case’ scenario for all-cause mortality. (TIF)

S15 Fig Forest plot of ‘best-worst case’ scenario for heart failure. (TIF)

S16 Fig Forest plot of ‘worst-best case’ scenario for heart failure. (TIF)

S17 Fig Forest plot of the comparison of different types of control interventions for all-cause mortality. (TIF)

S18 Fig Forest plot of the comparison of trials with different age of participants for all-cause mortality. (TIF)

S19 Fig Forest plot of the comparison of trials with different durations of atrial fibrillation for all-cause mortality. (TIF)

S20 Fig Forest plot of the comparison of trials with participants with heart failure to trials without participants with heart failure for all-cause mortality. (TIF)

S21 Fig Forest plot of the comparison of different types of control interventions for serious adverse events. (TIF)

S22 Fig Forest plot of the comparison of trials with different age of participants for serious adverse events. (TIF)

S23 Fig Forest plot of the comparison of trials with different durations of atrial fibrillation for serious adverse events. (TIF)

S24 Fig Forest plot of the comparison of trials with participants with heart failure to trials without participants with heart failure for serious adverse events. (TIF)

S25 Fig Forest plot of AF-SFS. (TIF)

S26 Fig Trial sequential analysis of AF-SFS. (TIF)

S27 Fig Forest plot of AF-SSS. (TIF)

S28 Fig Trial sequential analysis of AF-SSS. (TIF)

S29 Fig Forest plot of SF-36 PCS. (TIF)

S30 Fig Trial sequential analysis of SF-36 PCS. (TIF)

S31 Fig Forest plot of SF-36 MCS. (TIF)

S32 Fig Trial sequential analysis of SF-36 MCS. (TIF)

S33 Fig Forest plot of digoxin versus placebo on heart rate control 6 to 24 hours after treatment onset. (TIF)

S34 Fig Trial sequential analysis of digoxin versus placebo on heart rate control 6 to 24 hours after treatment onset. (TIF)

S35 Fig Forest plot of digoxin versus placebo on conversion to sinus rhythm within 6 hours of treatment onset. (TIF)

S36 Fig Trial sequential analysis of digoxin versus placebo on conversion to sinus rhythm within 6 hours of treatment onset. (TIF)

S37 Fig Forest plot of digoxin versus placebo on conversion to sinus rhythm 6 to 24 hours after treatment onset. (TIF)

S38 Fig Trial sequential analysis of digoxin versus placebo on conversion to sinus rhythm 6 to 24 hours after treatment onset. (TIF)

S39 Fig Forest plot of digoxin versus beta blockers on heart rate control 6 to 24 hours after treatment onset. (TIF)

S40 Fig Trial sequential analysis of digoxin versus beta blockers on heart rate control 6 to 24 hours after treatment onset. (TIF)

S41 Fig Forest plot of digoxin versus beta blockers on conversion to sinus rhythm within 6 hours of treatment onset. (TIF)

S42 Fig Trial sequential analysis of digoxin versus beta blockers on conversion to sinus rhythm within 6 hours of treatment onset. (TIF)

S43 Fig Forest plot of digoxin versus beta blockers on conversion to sinus rhythm 6 to 24 hours after treatment onset. (TIF)

S44 Fig Trial sequential analysis of digoxin versus beta blockers on conversion to sinus rhythm 6 to 24 hours after treatment onset. (TIF)

S45 Fig Forest plot of ‘best-worst case’ scenario of digoxin versus beta blockers on heart rate control within 6 hours of treatment onset. (TIF)

S46 Fig Forest plot of ‘worst-best case’ scenario of digoxin versus beta blockers on heart rate control within 6 hours of treatment onset. (TIF)

S47 Fig Forest plot of ‘best-worst case’ scenario of digoxin versus beta blockers on heart rate control 6 to 24 hours after treatment onset. (TIF)

S48 Fig Forest plot of ‘worst-best case’ scenario of digoxin versus beta blockers on heart rate control 6 to 24 hours after treatment onset. (TIF)

S49 Fig Forest plot of ‘best-worst case’ scenario of digoxin versus beta blockers on conversion to sinus rhythm within 6 hours of treatment onset. (TIF)

S50 Fig Forest plot of ‘worst-best case’ scenario of digoxin versus beta blockers on conversion to sinus rhythm within 6 hours of treatment onset. (TIF)

S51 Fig Forest plot of ‘best-worst case’ scenario of digoxin versus beta blockers on conversion to sinus rhythm 6 to 24 hours after treatment onset. (TIF)

S52 Fig Forest plot of ‘worst-best case’ scenario of digoxin versus beta blockers on conversion to sinus rhythm 6 to 24 hours after treatment onset. (TIF)

S53 Fig Forest plot of digoxin versus calcium antagonists on heart rate control within 6 hours of treatment onset. (TIF)

S54 Fig Trial sequential analysis of digoxin versus calcium antagonists on heart rate control within 6 hours of treatment onset. (TIF)

S55 Fig Forest plot of digoxin versus calcium antagonists on heart rate control 6 to 24 hours after tratment onset. (TIF)

S56 Fig Trial sequential analysis of digoxin versus calcium antagonists on heart rate control 6 to 24 hours after treatment onset. (TIF)

S57 Fig Forest plot of digoxin versus calcium antagonists on conversion to sinus rhythm within 6 hours of treatment onset. (TIF)

S58 Fig Trial sequential analysis of digoxin versus calcium antagonists on conversion to sinus rhythm within 6 hours of treatment onset. (TIF)

S59 Fig Forest plot of digoxin versus calcium antagonists on conversion to sinus rhythm 6 to 24 hours after treatment onset. (TIF)

S60 Fig Trial sequential analysis of digoxin versus calcium antagonists on conversion to sinus rhythm 6 to 24 hours after treatment onset. (TIF)

S61 Fig Forest plot of ‘best-worst case’ scenario of digoxin versus calcium antagonists on conversion to sinus rhythm within 6 hours of treatment onset. (TIF)

S62 Fig Forest plot of ‘worst-best case’ scenario of digoxin versus calcium antagonists on conversion to sinus rhythm within 6 hours of treatment onset. (TIF)

S63 Fig Forest plot of ‘best-worst case’ scenario of digoxin versus calcium antagonists on conversion to sinus rhythm 6 to 24 hours after treatment onset. (TIF)

S64 Fig Forest plot of ‘worst-best case’ scenario of digoxin versus calcium antagonists on conversion to sinus rhythm 6 to 24 hours after treatment onset. (TIF)

S65 Fig Forest plot of digoxin versus amiodarone on heart rate control within 6 hours of treatment onset. (TIF)

S66 Fig Trial sequential analysis of digoxin versus amiodarone on heart rate control within 6 hours of treatment onset. (TIF)

S67 Fig Forest plot of digoxin versus amiodarone on conversion to sinus rhythm within 6 hours of treatment onset. (TIF)

S68 Fig Trial sequential analysis of digoxin versus amiodarone on conversion to sinus rhythm within 6 hours of treatment onset. (TIF)

S69 Fig Forest plot of digoxin versus amiodarone on heart rate control 6 to 24 hours after treatment onset. (TIF)

S70 Fig Trial sequential analysis of digoxin versus amiodarone on heart rate control 6 to 24 hours after treatment onset. (TIF)

S71 Fig Forest plot of digoxin versus amiodarone on conversion to sinus rhythm 6 to 24 hours after treatment onset. (TIF)

S72 Fig Trial sequential analysis of digoxin versus amiodarone on conversion to sinus rhythm 6 to 24 hours after treatment onset. (TIF)

S73 Fig Forest plot of ‘best-worst case’ scenario of digoxin versus amiodarone on heart rate control within 6 hours of treatment onset. (TIF)

S74 Fig Forest plot of ‘worst-best case’ scenario of digoxin versus amiodarone on heart rate control within 6 hours of treatment onset. (TIF)

S75 Fig Forest plot of ‘best-worst case’ scenario of digoxin versus amiodarone on heart rate control 6 to 24 hours after treatment onset. (TIF)

S76 Fig Forest plot of ‘worst-best case’ scenario of digoxin versus amiodarone on heart rate control 6 to 24 hours after treatment onset. (TIF)

S77 Fig Forest plot of ‘best-worst case’ scenario of digoxin versus amiodarone on conversion to sinus rhythm 6 to 24 hours after treatment onset. (TIF)

S78 Fig Forest plot of ‘worst-best case’ scenario of digoxin versus amiodarone on conversion to sinus rhythm 6 to 24 hours after treatment onset. (TIF)

S79 Fig Forest plot of ‘best-worst case’ scenario of digoxin versus amiodarone on conversion to sinus rhythm within 6 hours of treatment onset. (TIF)

S80 Fig Forest plot of ‘worst-best case’ scenario of digoxin versus amiodarone on conversion to sinus rhythm within 6 hours of treatment onset. (TIF)

S1 Table Inclusion- and exclusion criteria for each trial included. (DOCX)

S2 Table Characteristics of each included, excluded, and ongoing study. (DOCX)

S3 Table Specific types of serious adverse events in each trial comparison. (DOCX)

S4 Table Heart rate control in all patients regardless of type of rhythm. (DOCX)
