# Supplementary figures and images for: Digoxin for atrial fibrillation and atrial flutter: A systematic review with meta-analysis and trial sequential analysis of randomised clinical trials
Source: PLoS One. 2018 Mar 8;13(3):e0193924. doi: 10.1371/journal.pone.0193924 (PMC5843263; doi:10.1371/journal.pone.0193924)

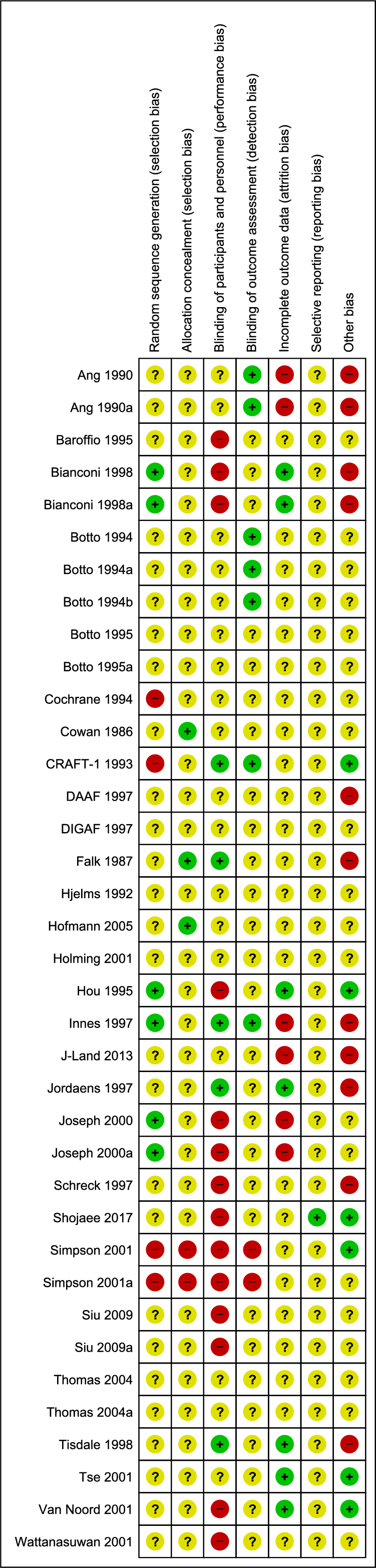

Supplement: S1 Fig — (TIF) [file pone.0193924.s004.tif]

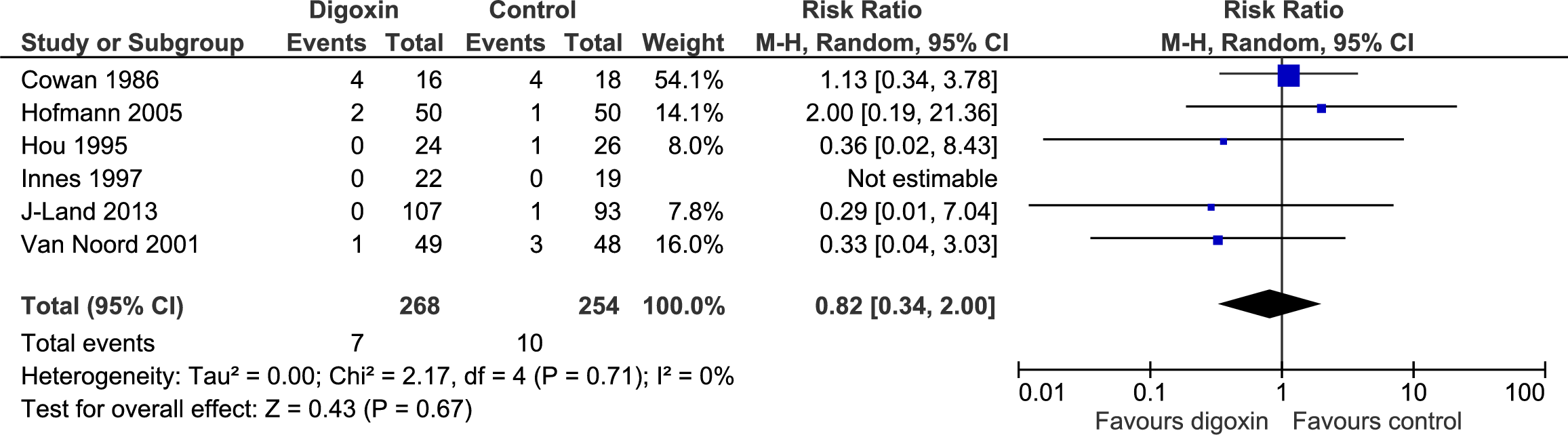

Supplement: S2 Fig — (TIF) [file pone.0193924.s005.tif]

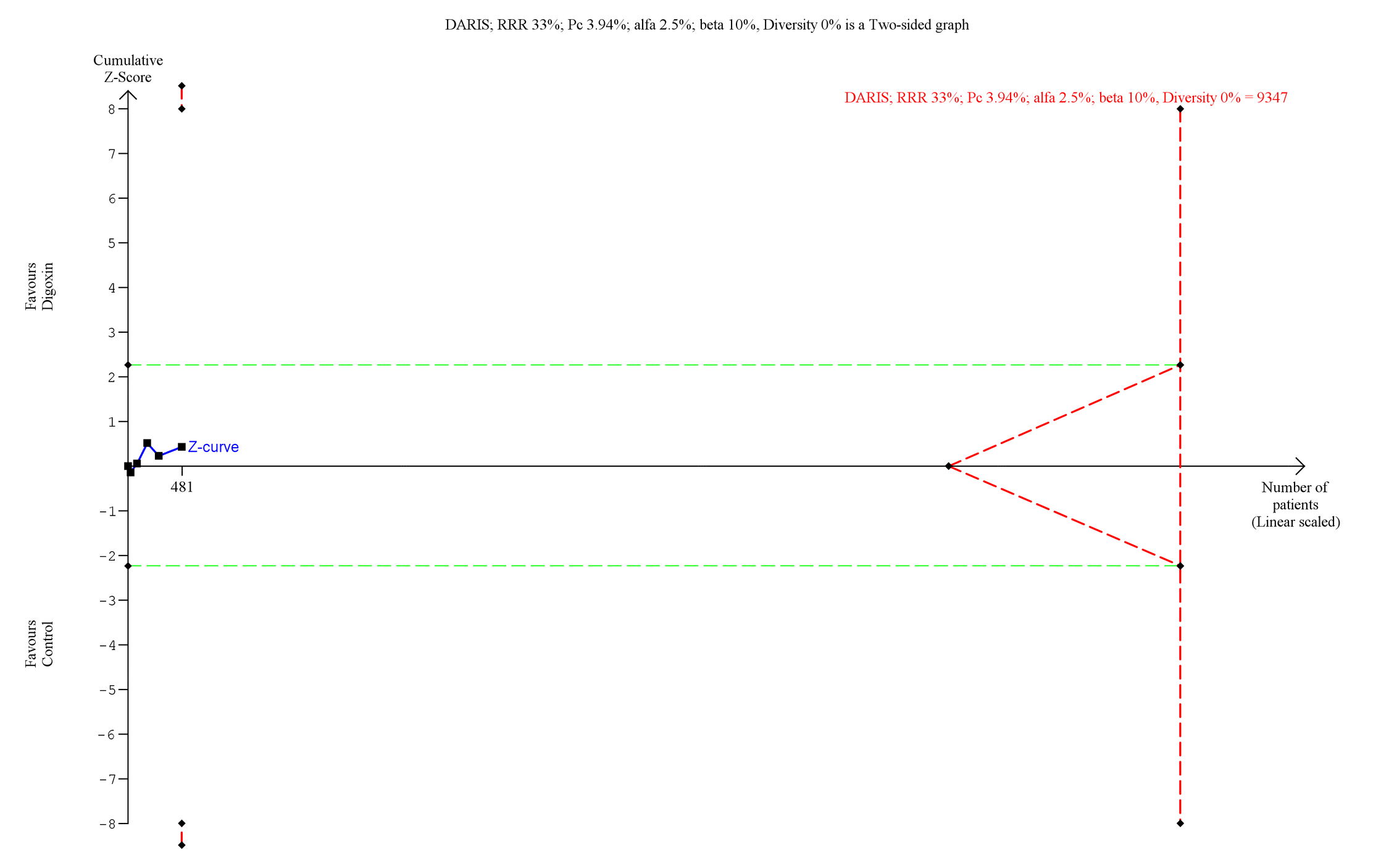

Supplement: S3 Fig — (TIF) [file pone.0193924.s006.tif]

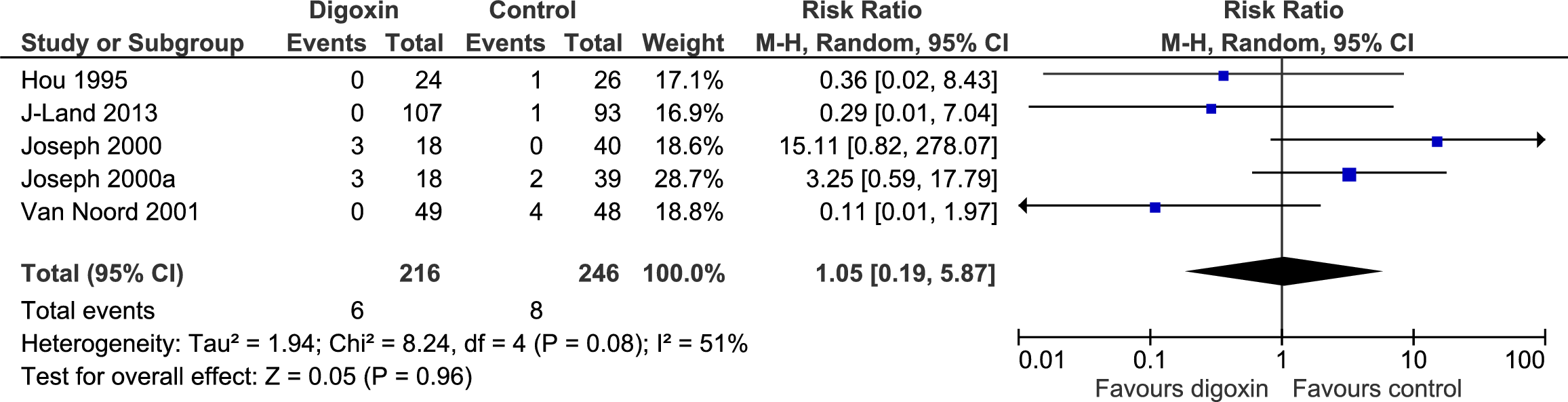

Supplement: S4 Fig — (TIF) [file pone.0193924.s007.tif]

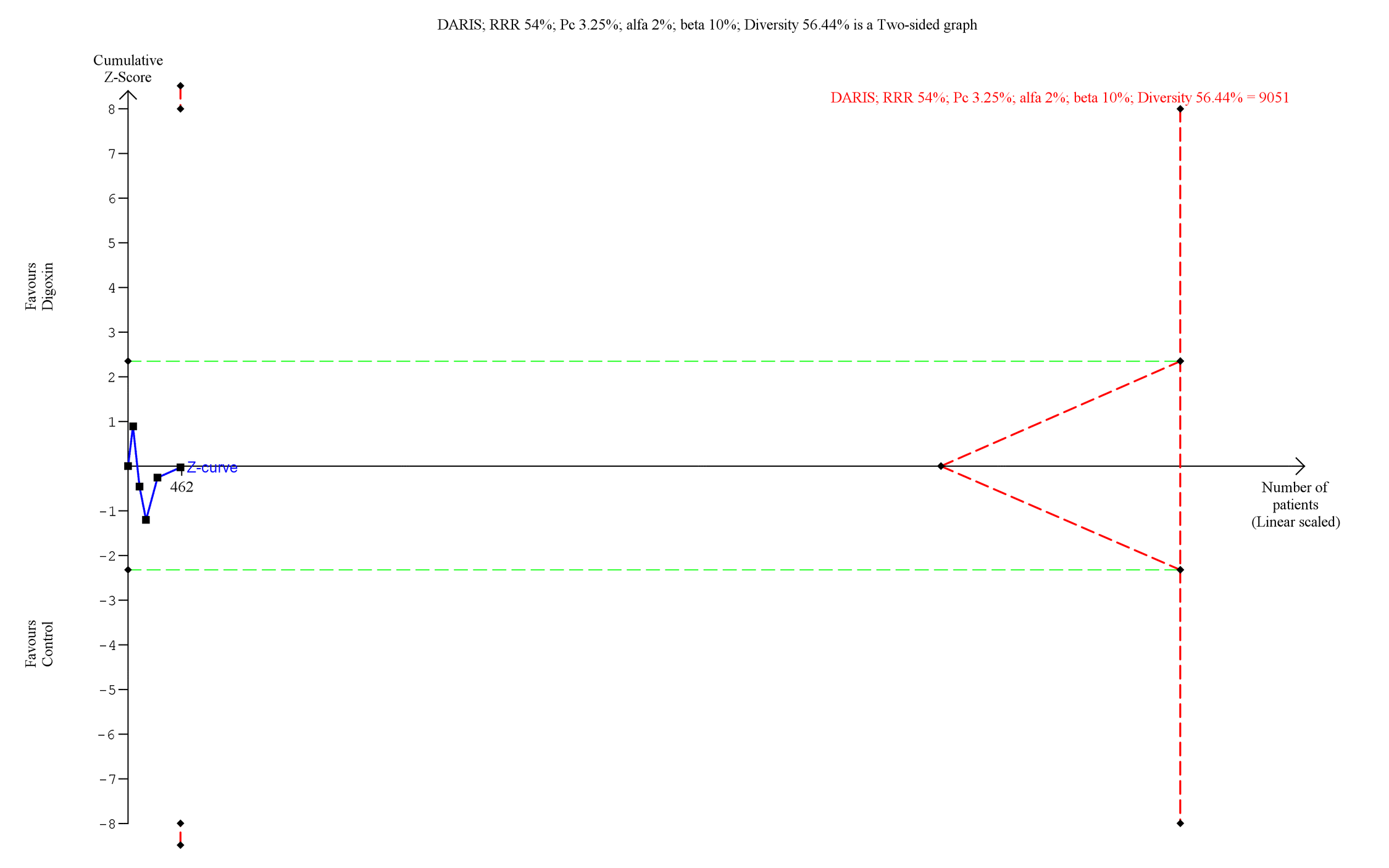

Supplement: S5 Fig — (TIF) [file pone.0193924.s008.tif]

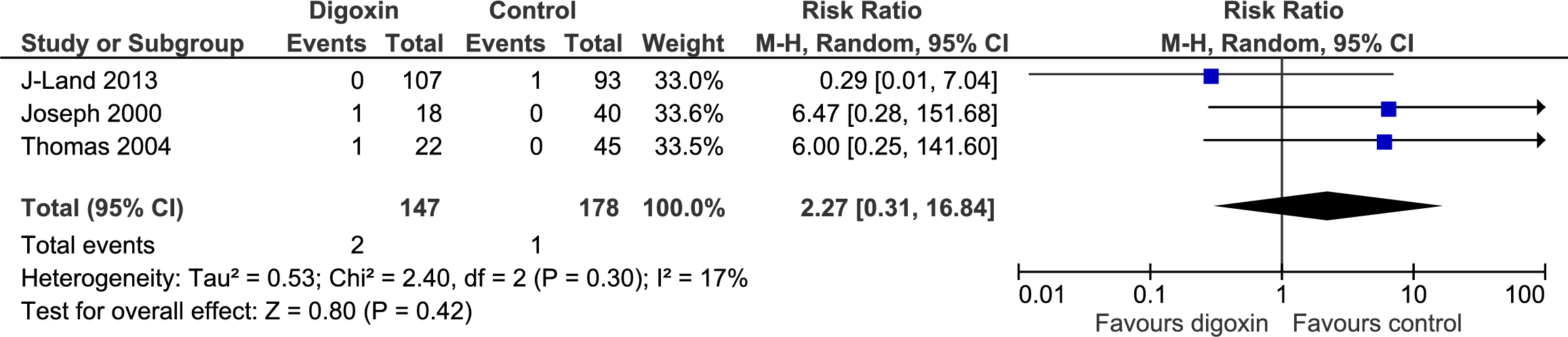

Supplement: S6 Fig — (TIF) [file pone.0193924.s009.tif]

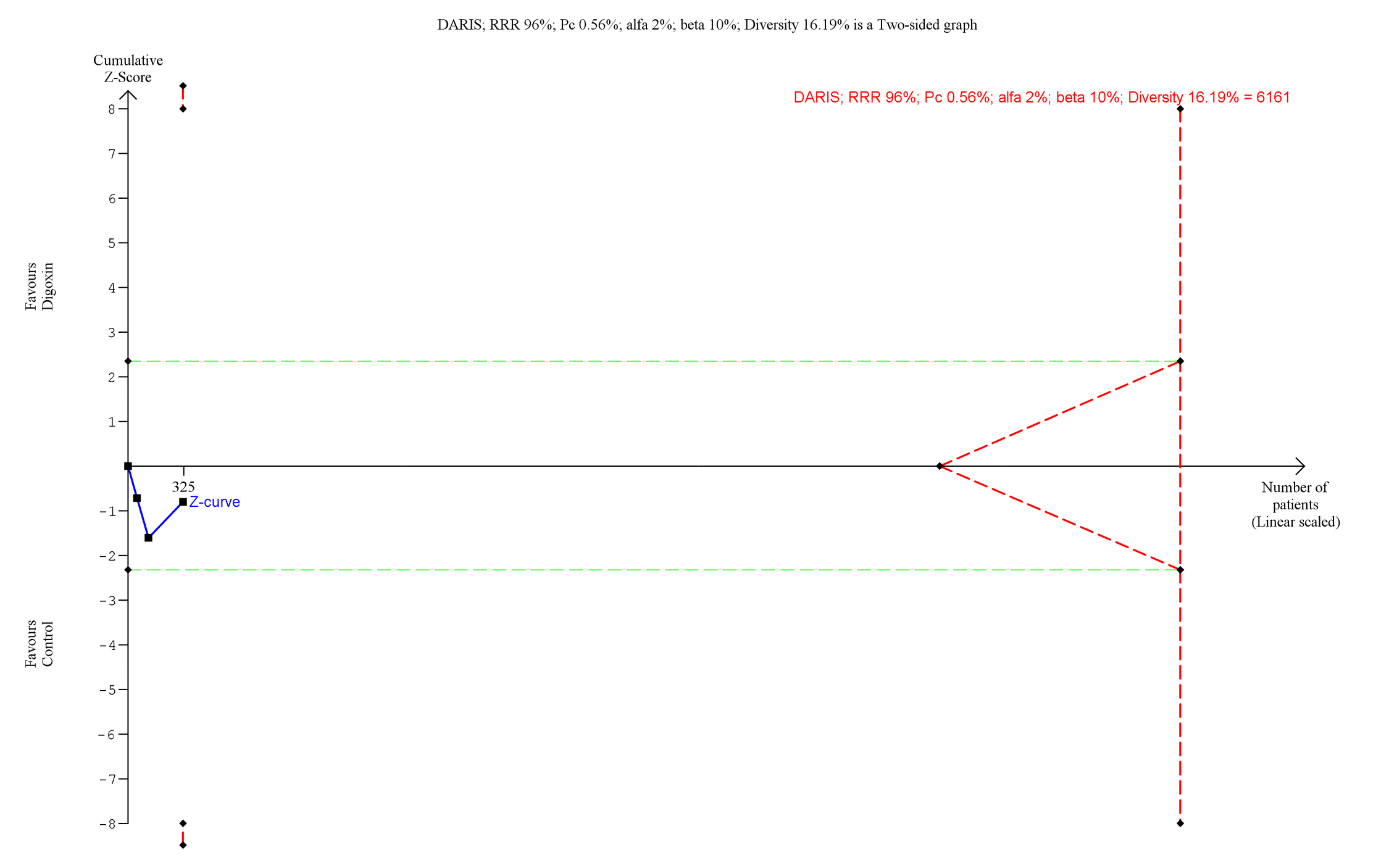

Supplement: S7 Fig — (TIF) [file pone.0193924.s010.tif]

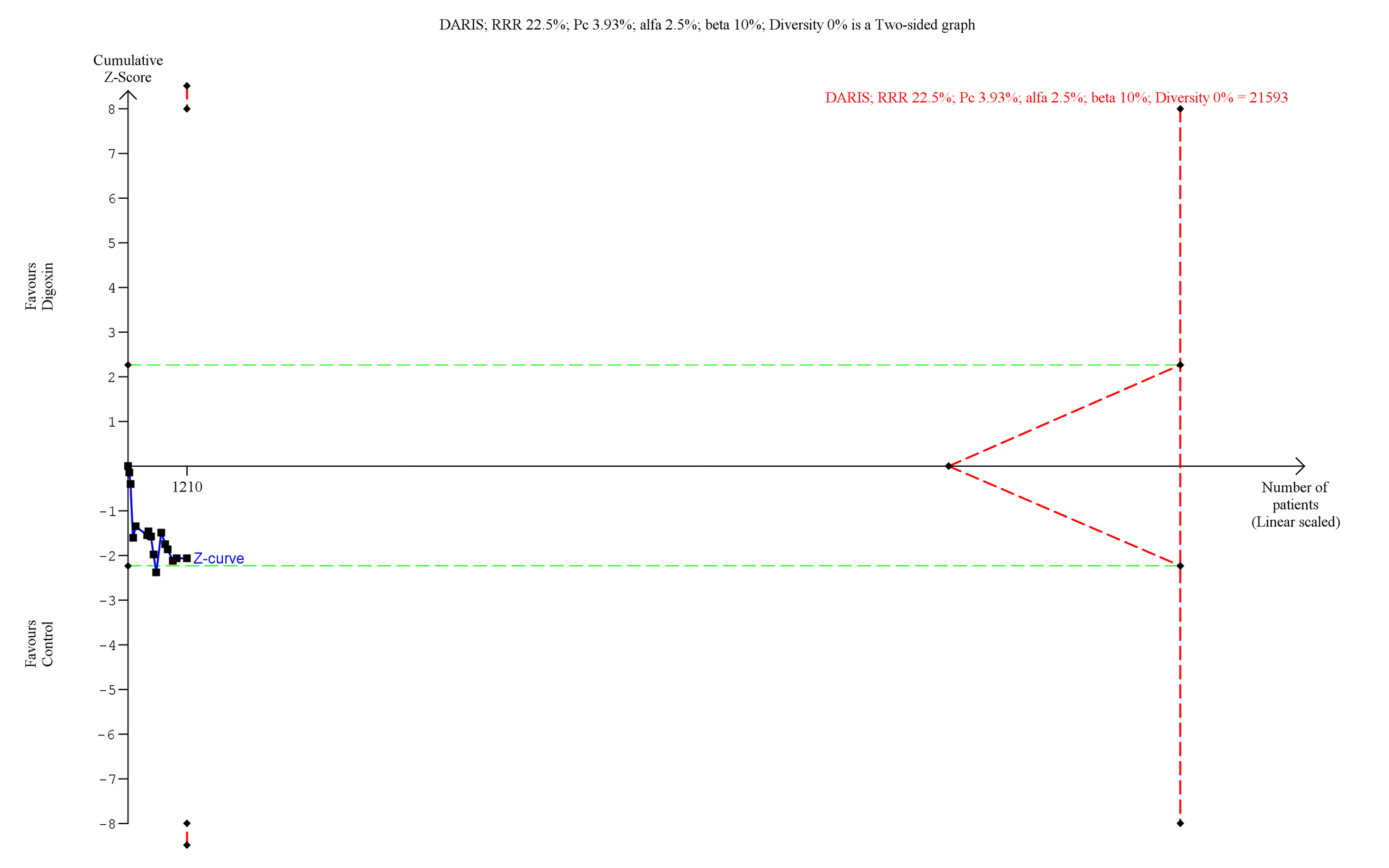

Supplement: S8 Fig — (TIF) [file pone.0193924.s011.tif]

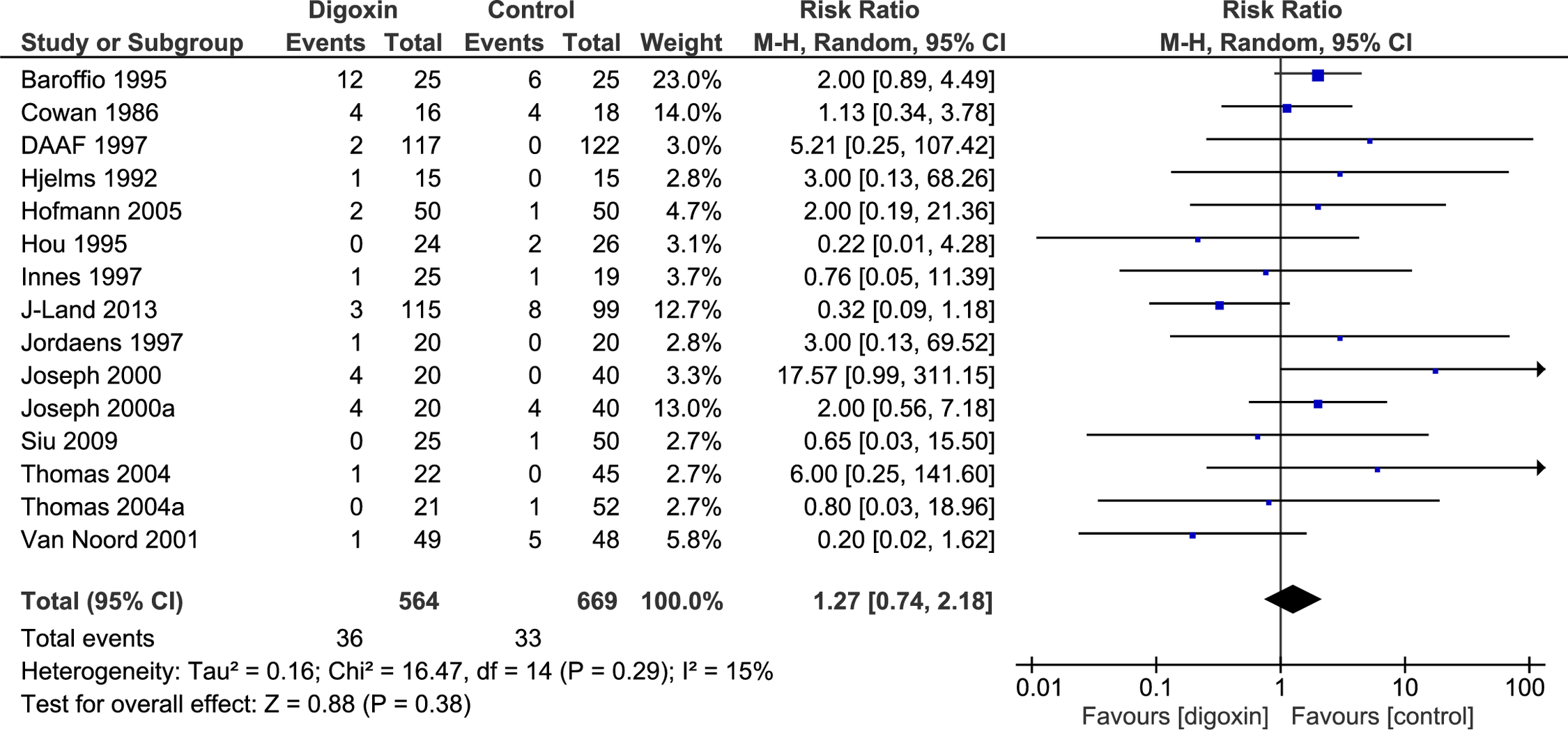

Supplement: S9 Fig — (TIF) [file pone.0193924.s012.tif]

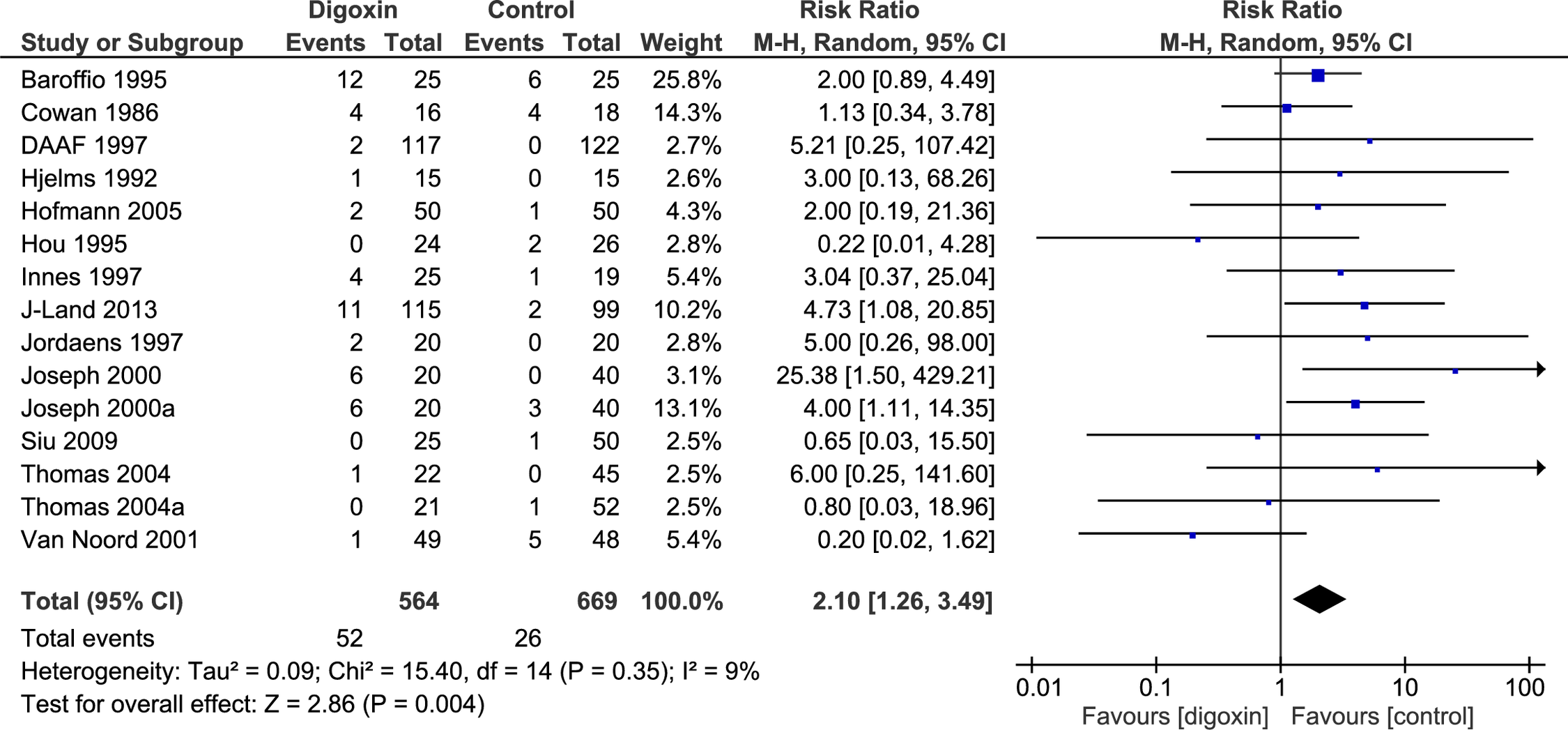

Supplement: S10 Fig — (TIF) [file pone.0193924.s013.tif]

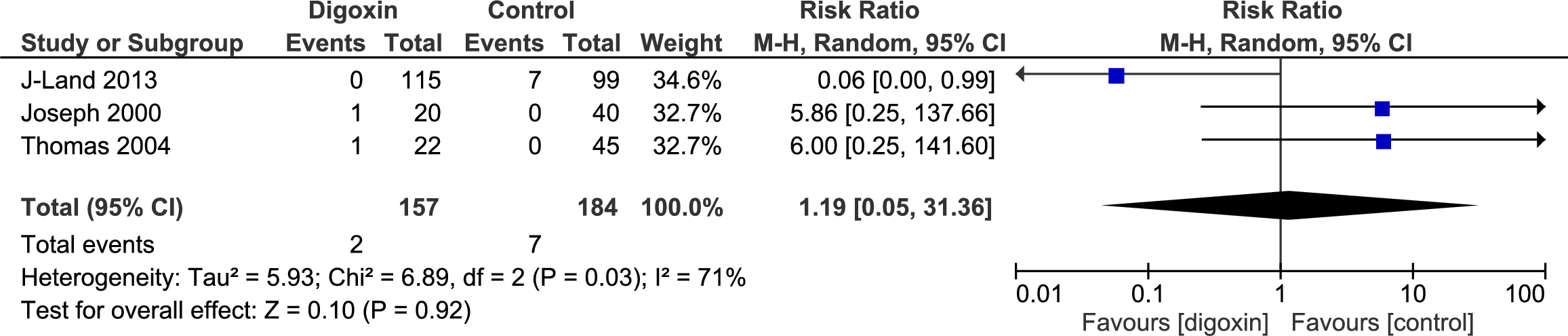

Supplement: S11 Fig — (TIF) [file pone.0193924.s014.tif]

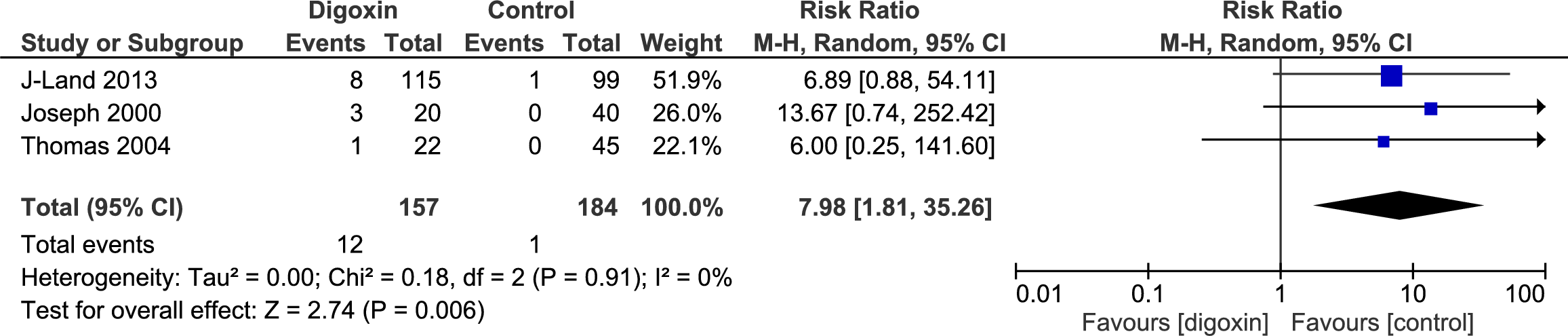

Supplement: S12 Fig — (TIF) [file pone.0193924.s015.tif]

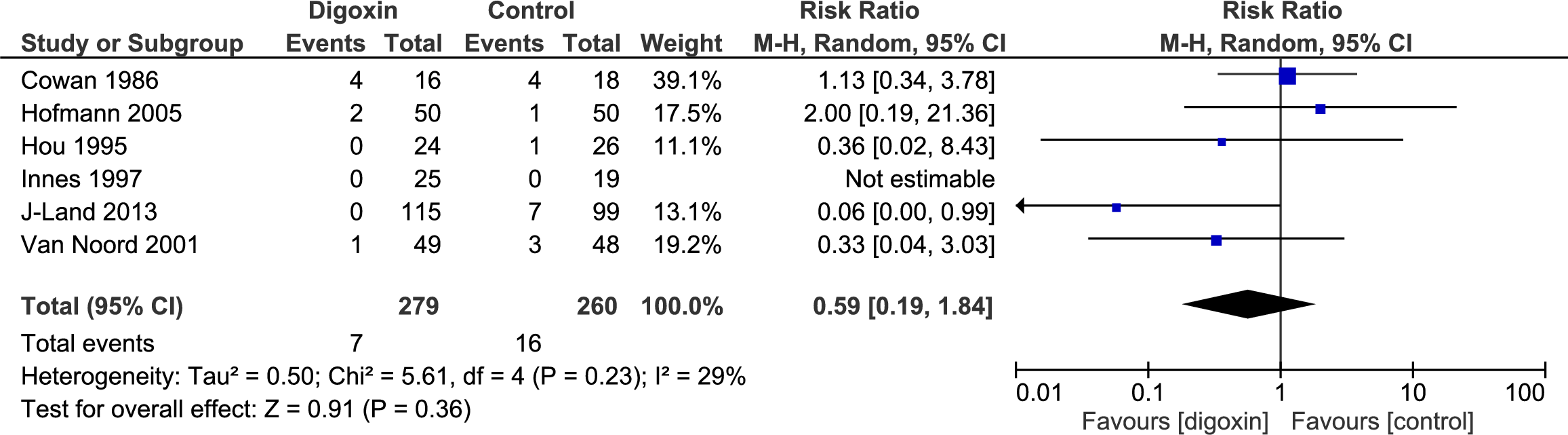

Supplement: S13 Fig — (TIF) [file pone.0193924.s016.tif]

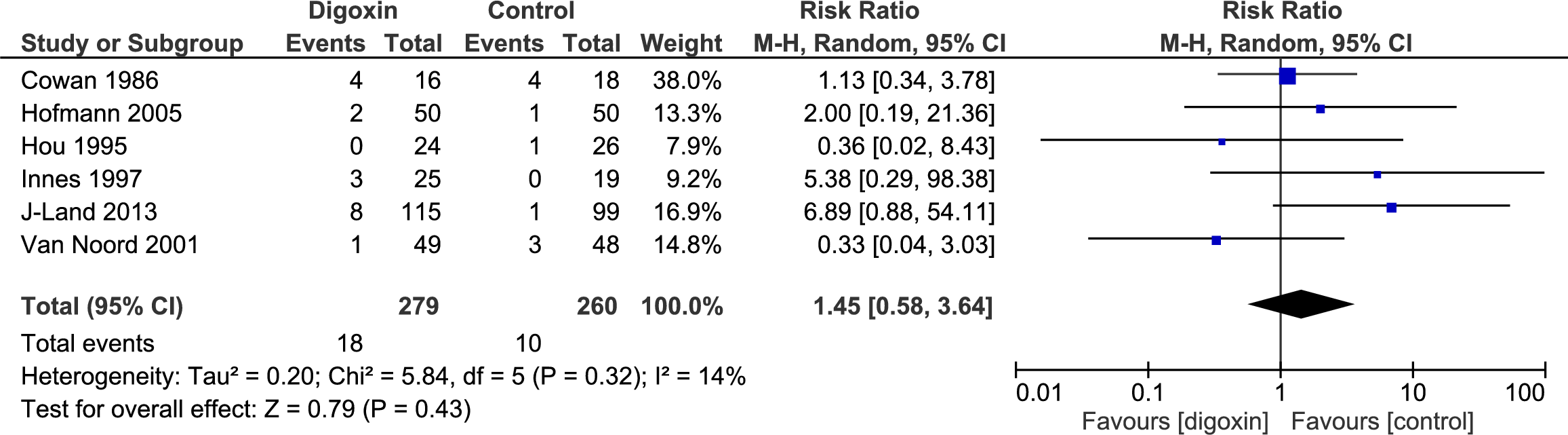

Supplement: S14 Fig — (TIF) [file pone.0193924.s017.tif]

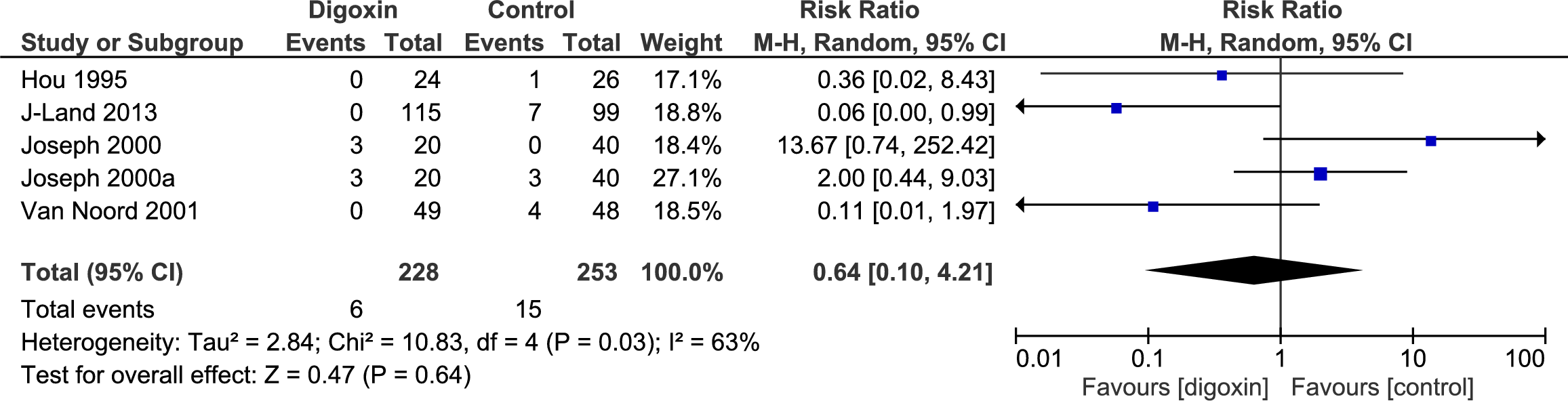

Supplement: S15 Fig — (TIF) [file pone.0193924.s018.tif]

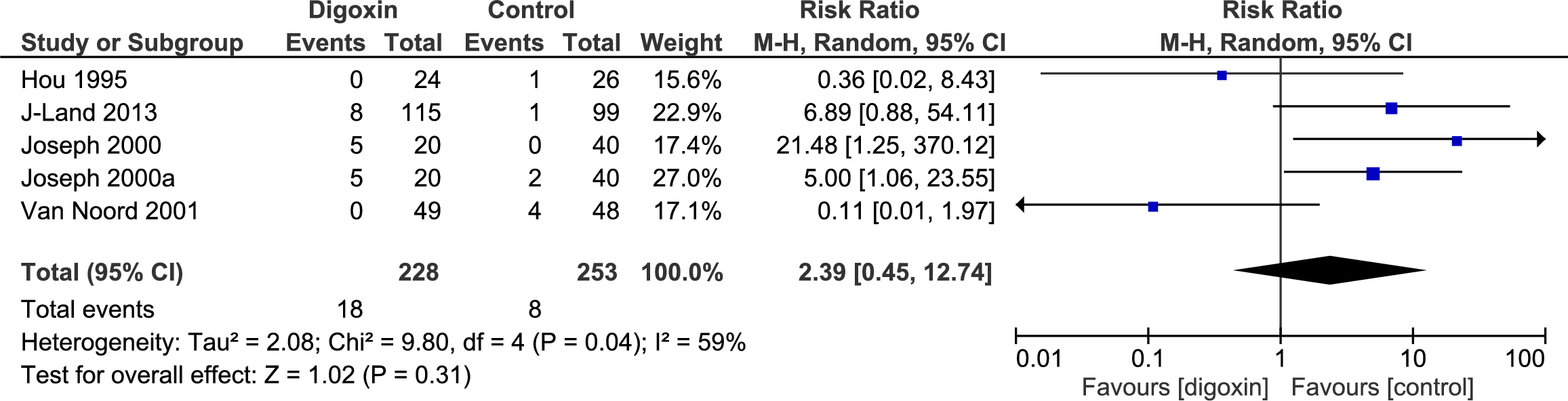

Supplement: S16 Fig — (TIF) [file pone.0193924.s019.tif]

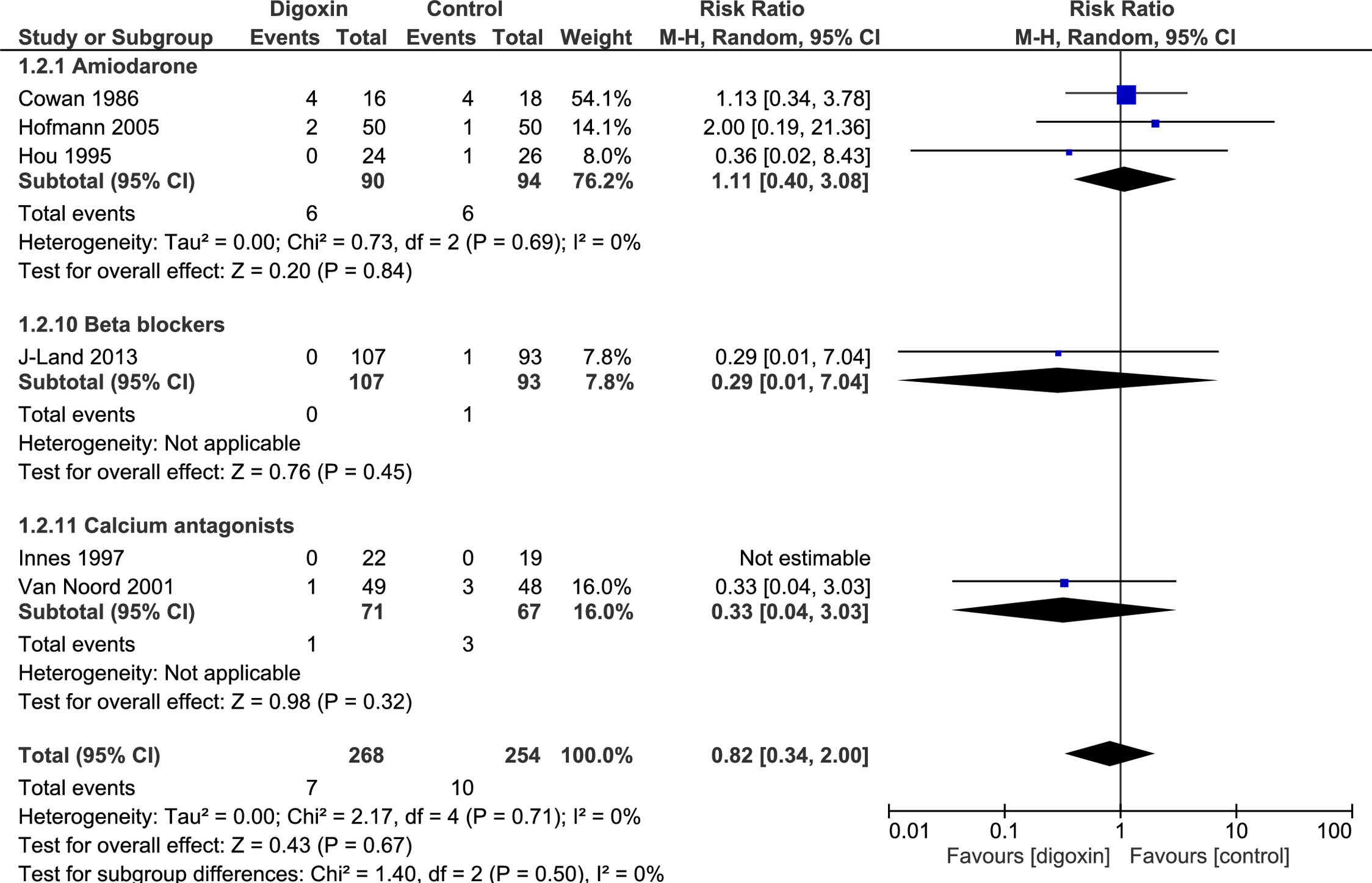

Supplement: S17 Fig — (TIF) [file pone.0193924.s020.tif]

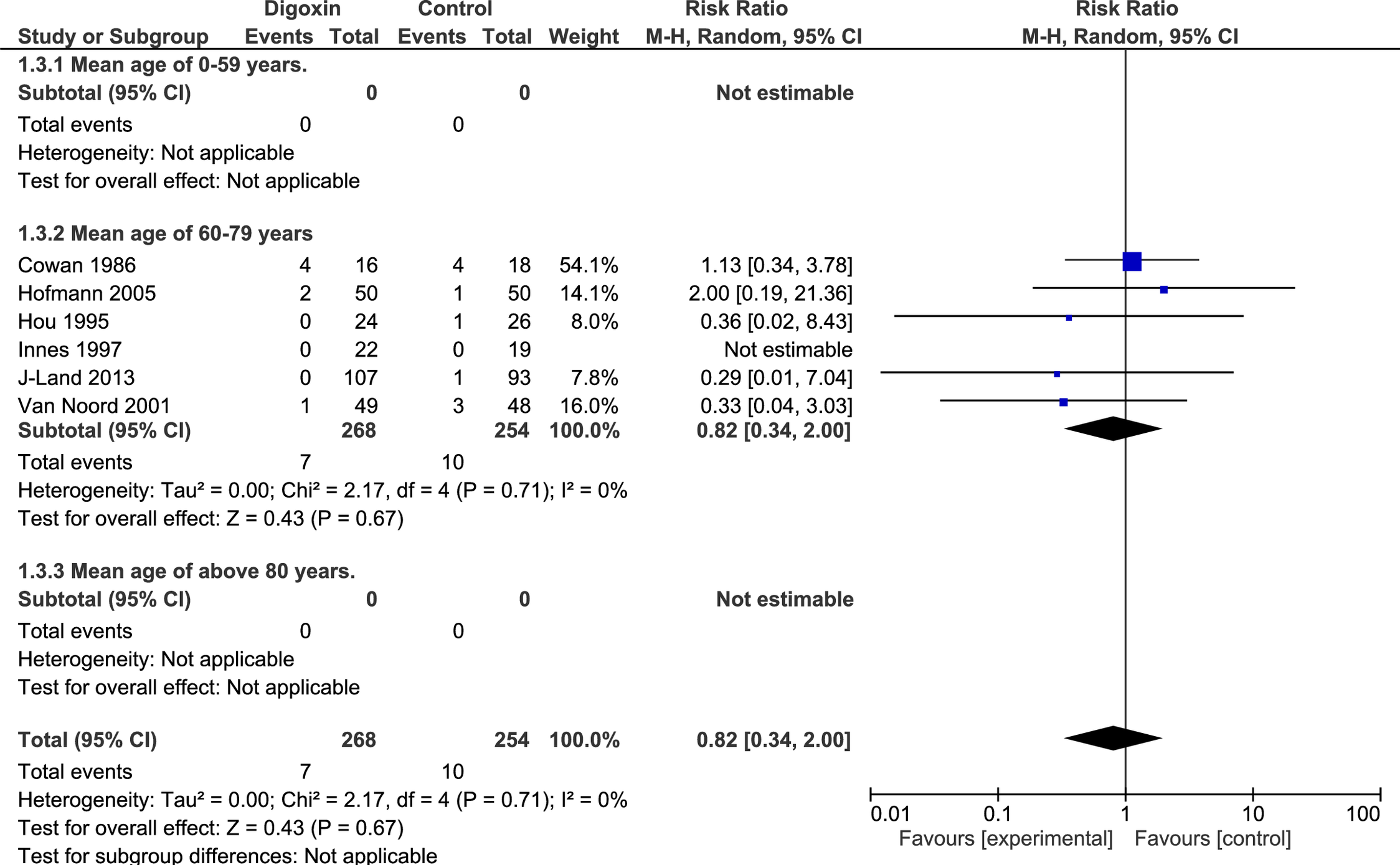

Supplement: S18 Fig — (TIF) [file pone.0193924.s021.tif]

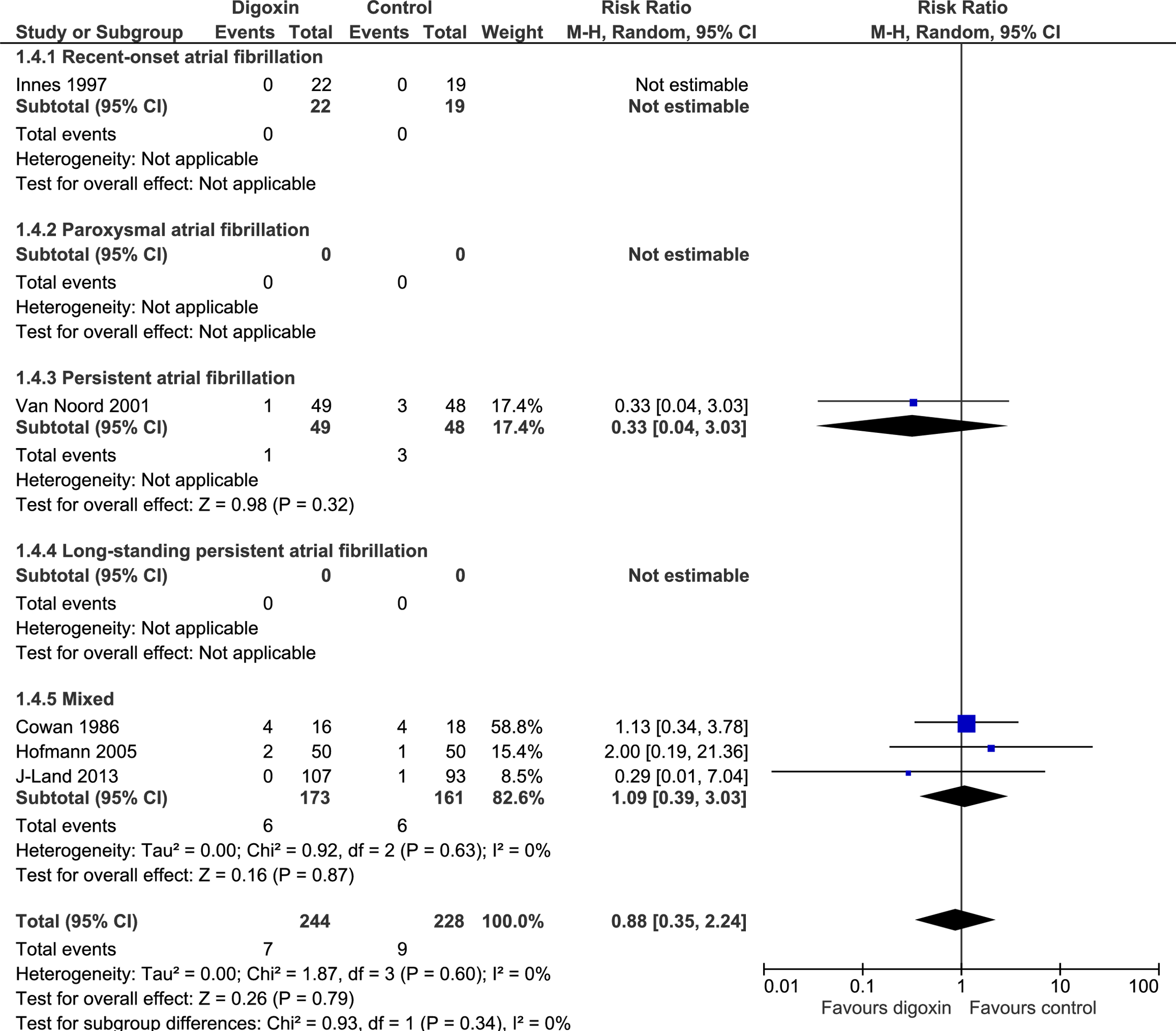

Supplement: S19 Fig — (TIF) [file pone.0193924.s022.tif]

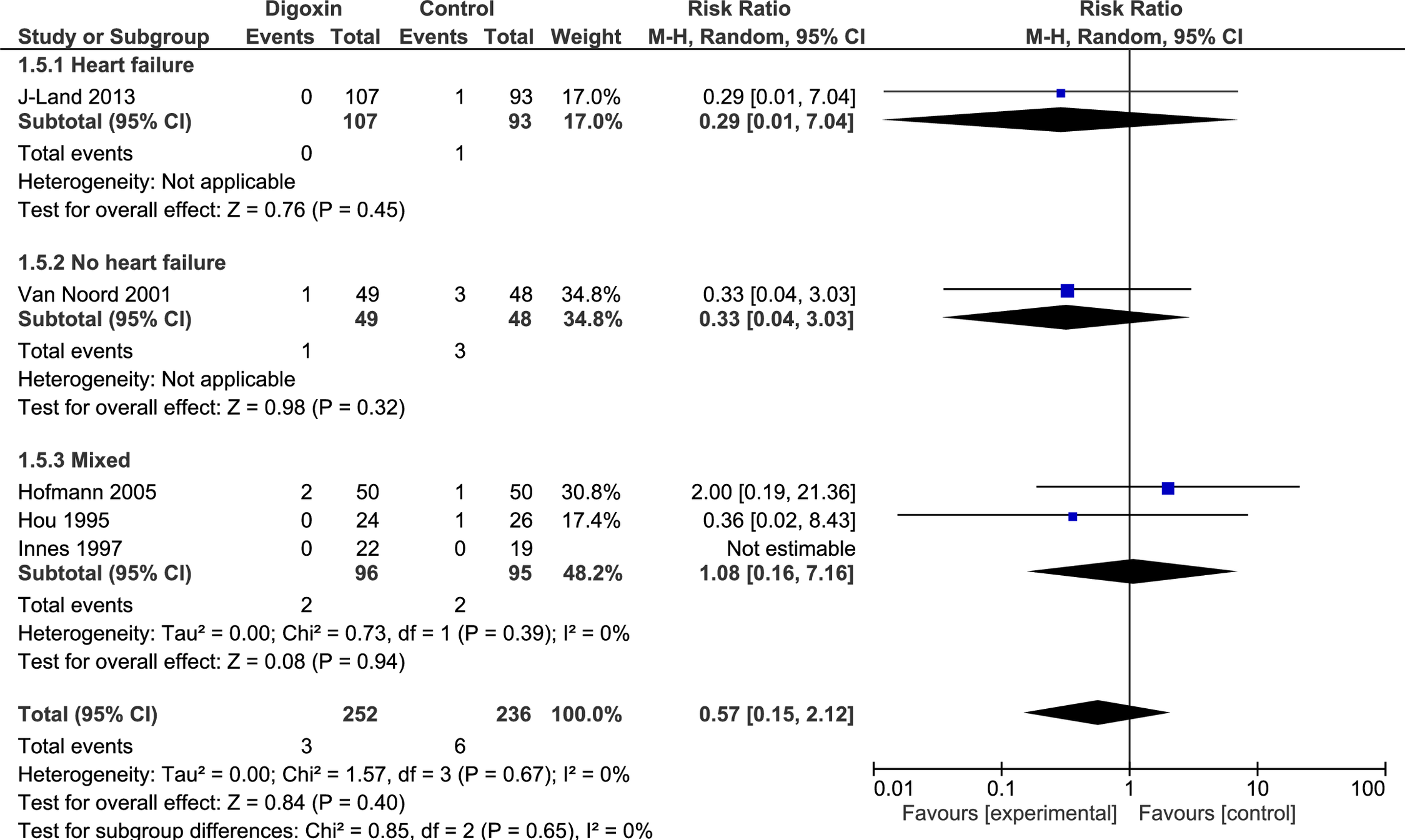

Supplement: S20 Fig — (TIF) [file pone.0193924.s023.tif]

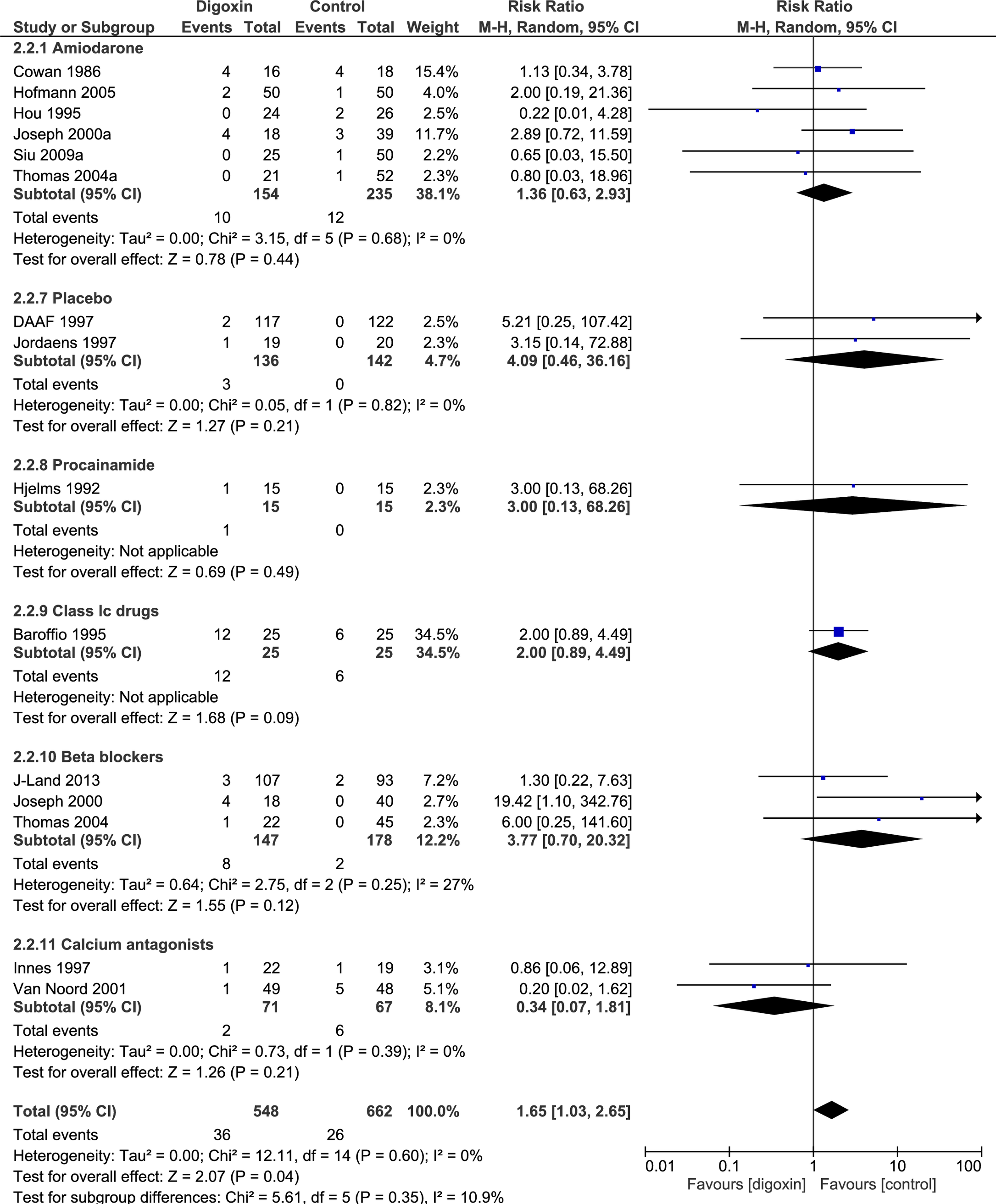

Supplement: S21 Fig — (TIF) [file pone.0193924.s024.tif]

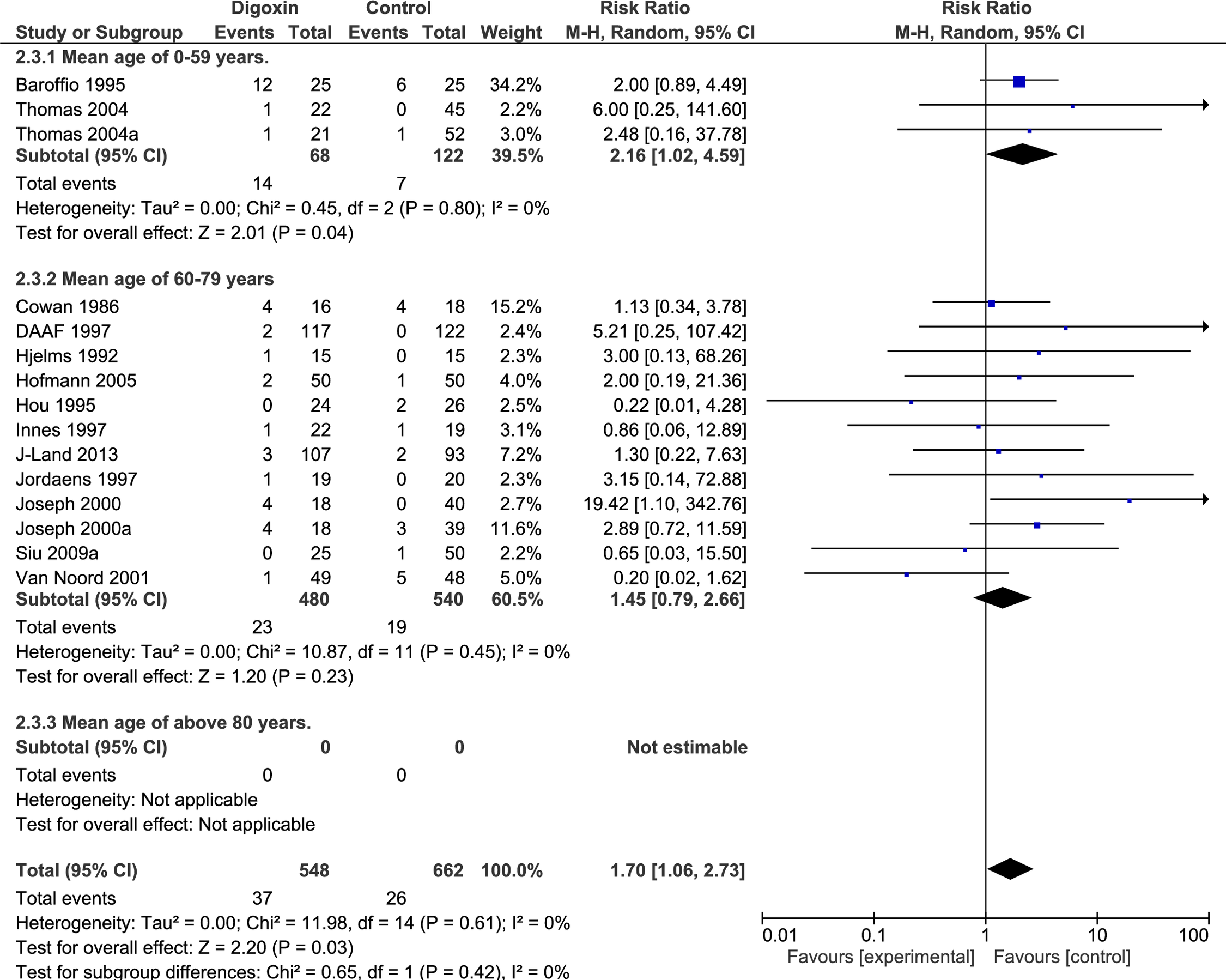

Supplement: S22 Fig — (TIF) [file pone.0193924.s025.tif]

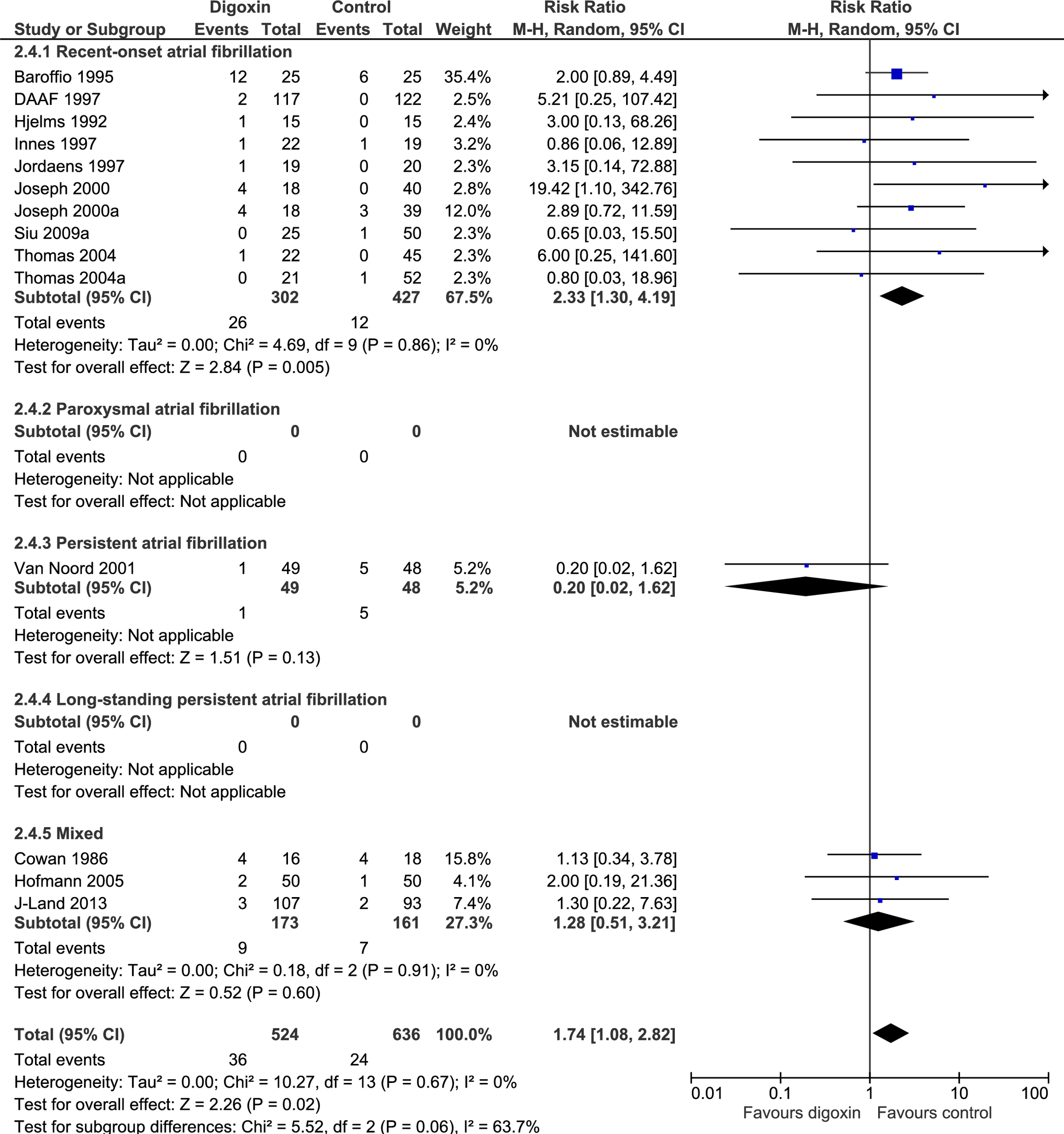

Supplement: S23 Fig — (TIF) [file pone.0193924.s026.tif]

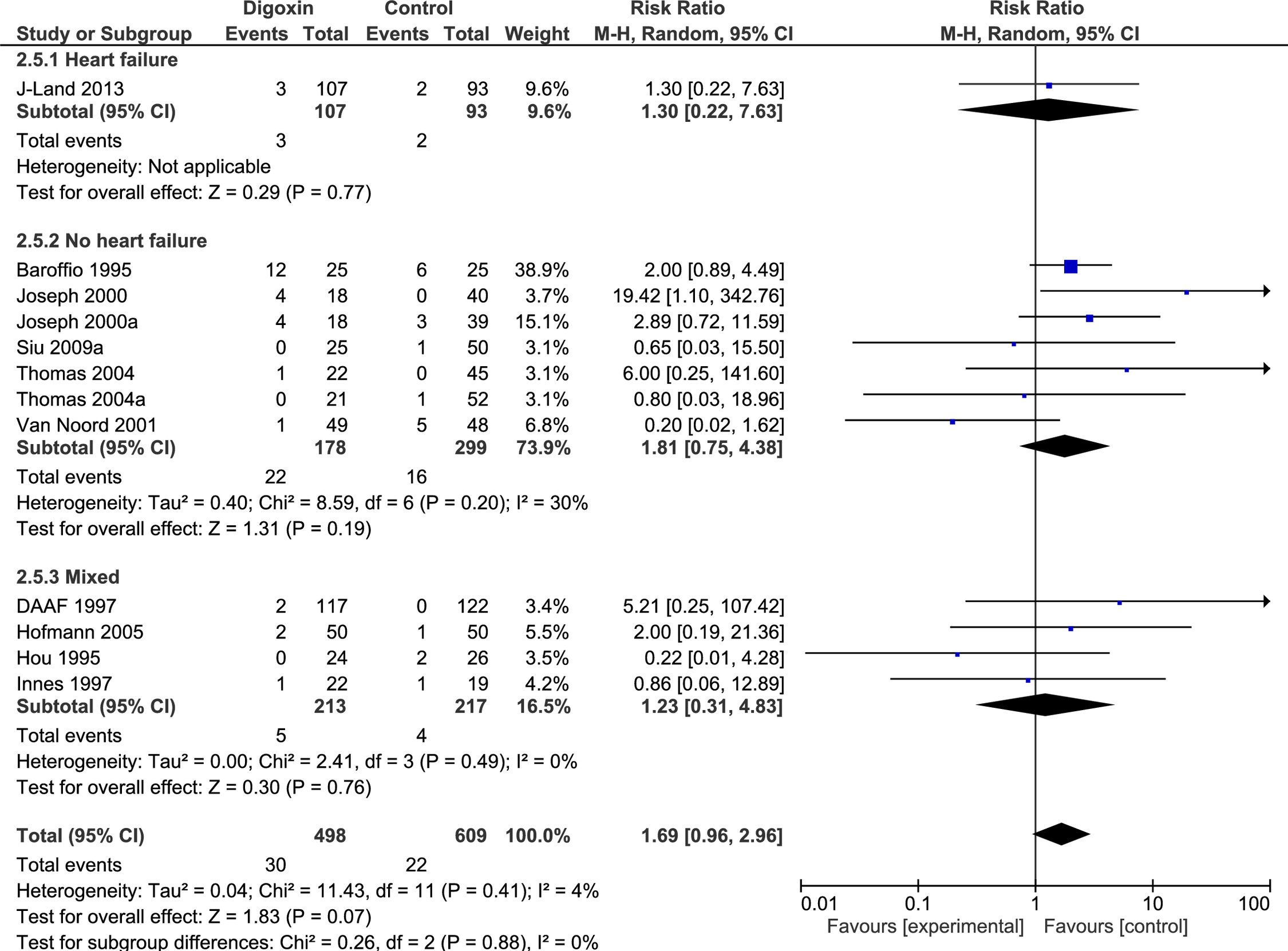

Supplement: S24 Fig — (TIF) [file pone.0193924.s027.tif]

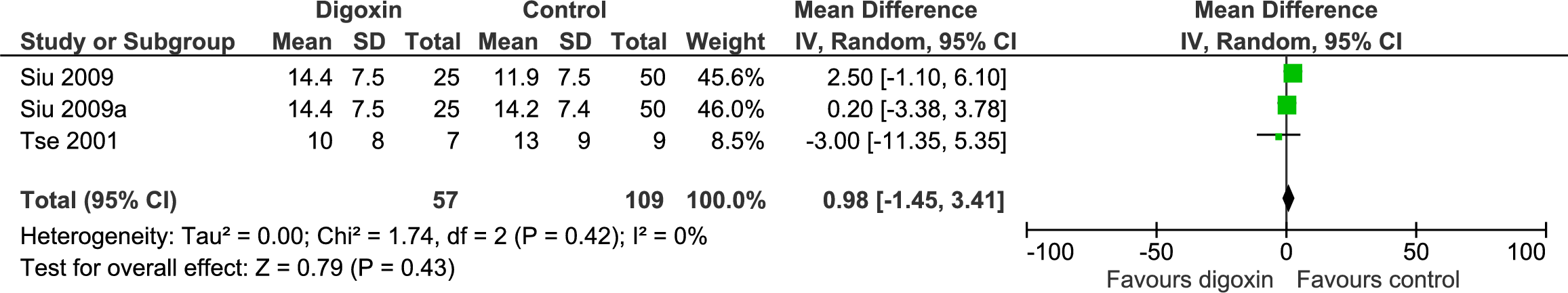

Supplement: S25 Fig — (TIF) [file pone.0193924.s028.tif]

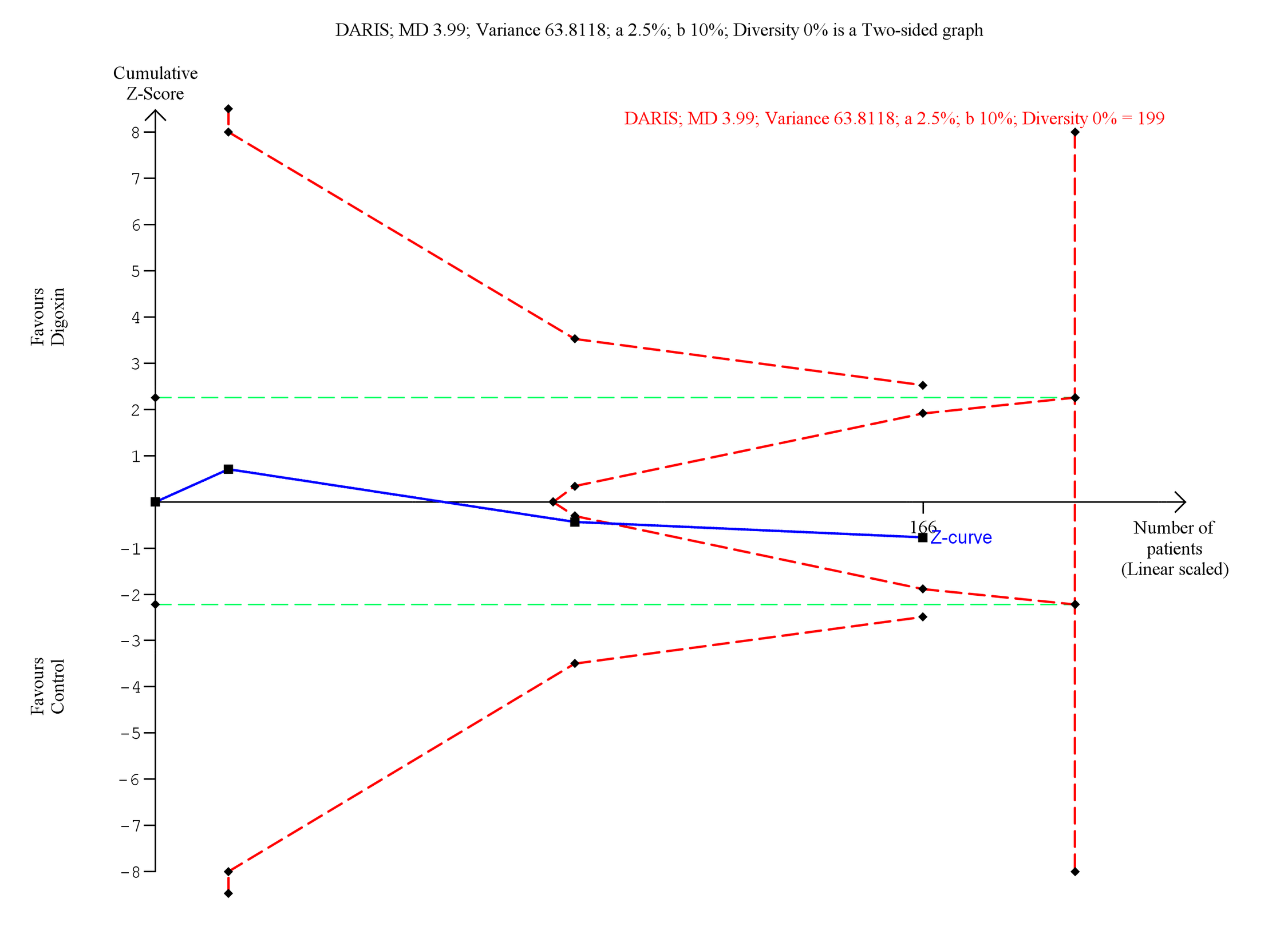

Supplement: S26 Fig — (TIF) [file pone.0193924.s029.tif]

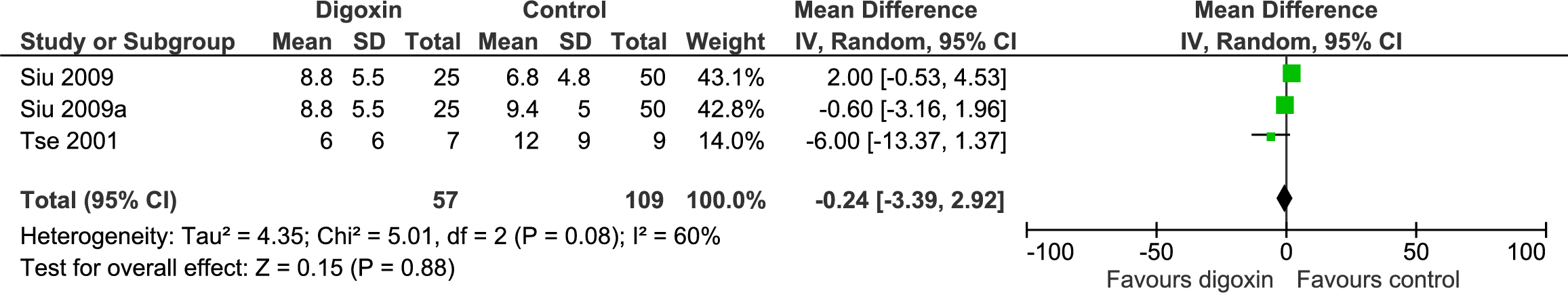

Supplement: S27 Fig — (TIF) [file pone.0193924.s030.tif]

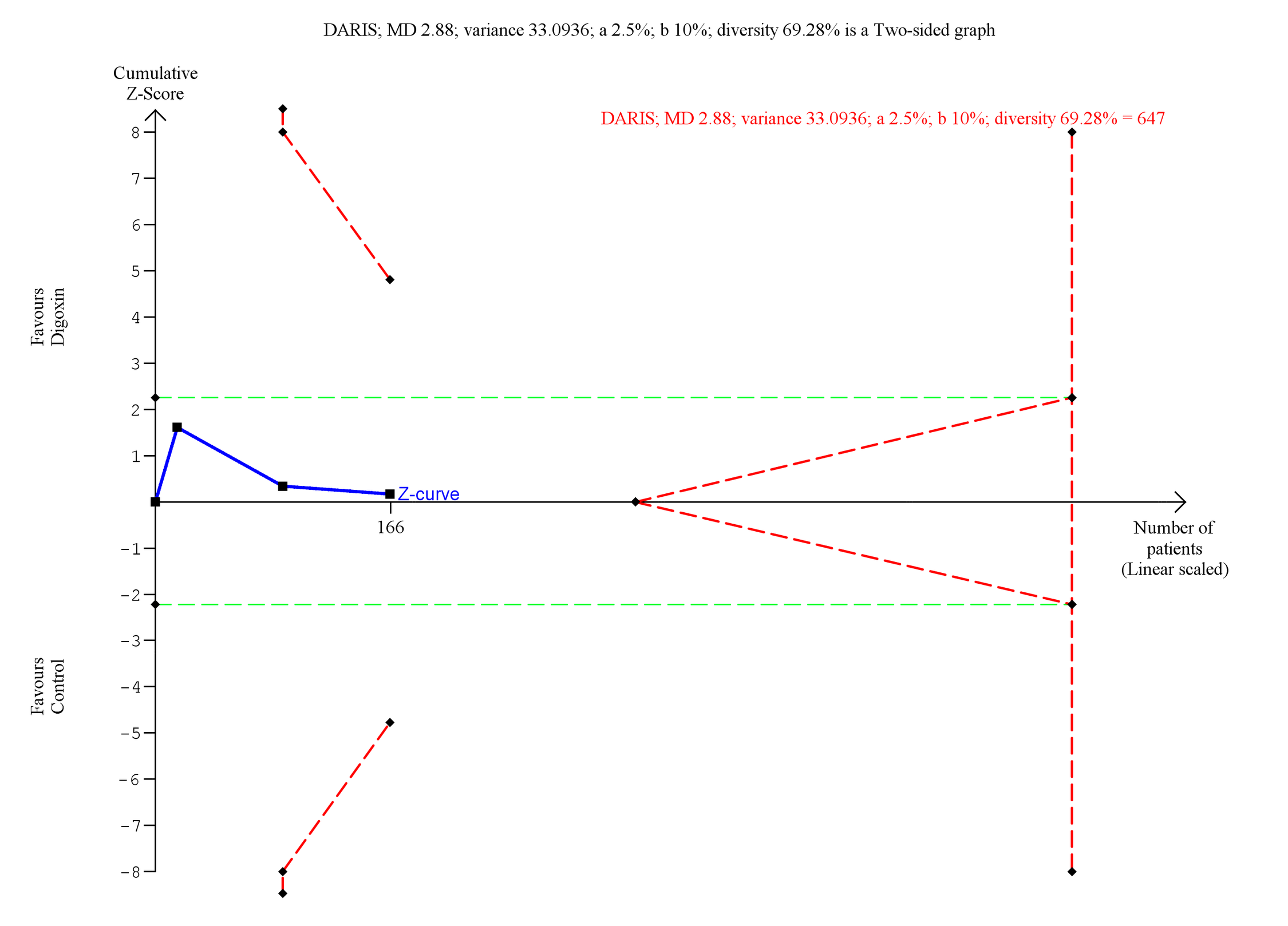

Supplement: S28 Fig — (TIF) [file pone.0193924.s031.tif]

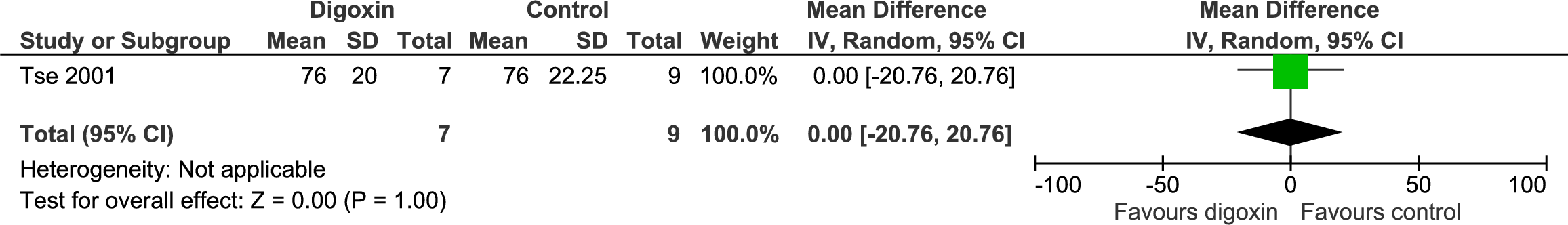

Supplement: S29 Fig — (TIF) [file pone.0193924.s032.tif]

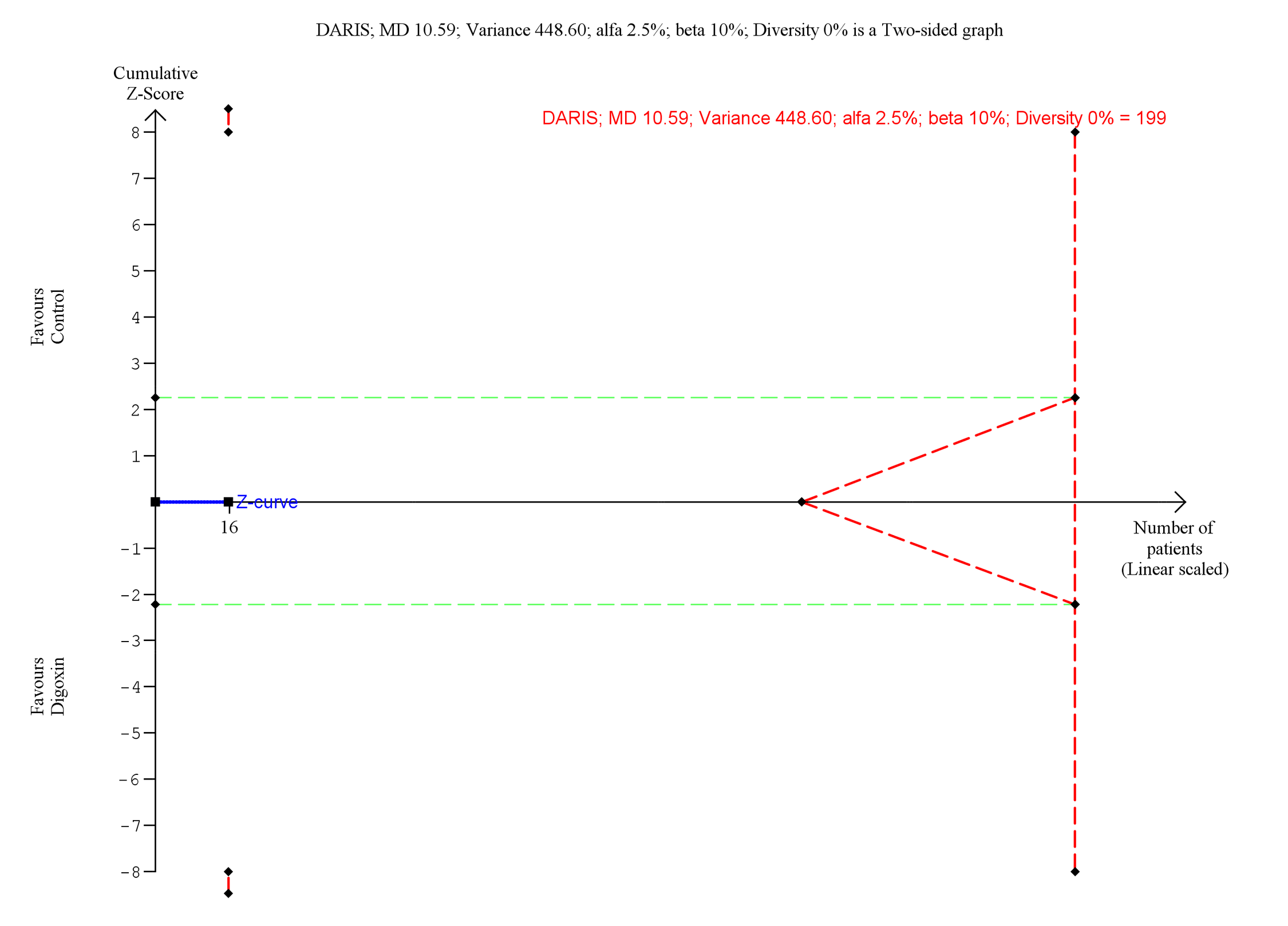

Supplement: S30 Fig — (TIF) [file pone.0193924.s033.tif]

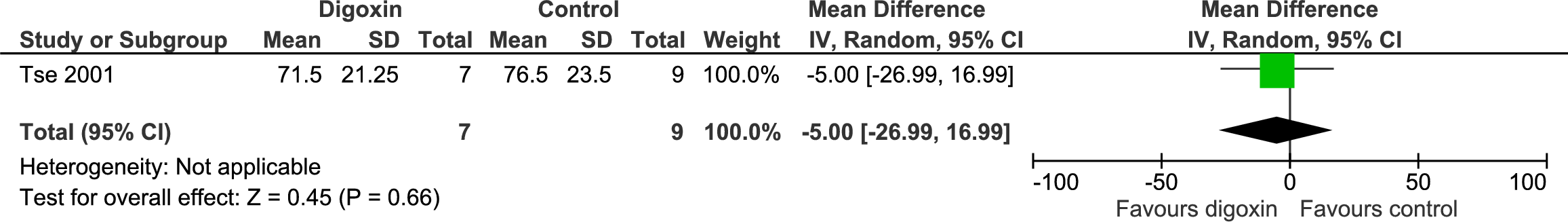

Supplement: S31 Fig — (TIF) [file pone.0193924.s034.tif]

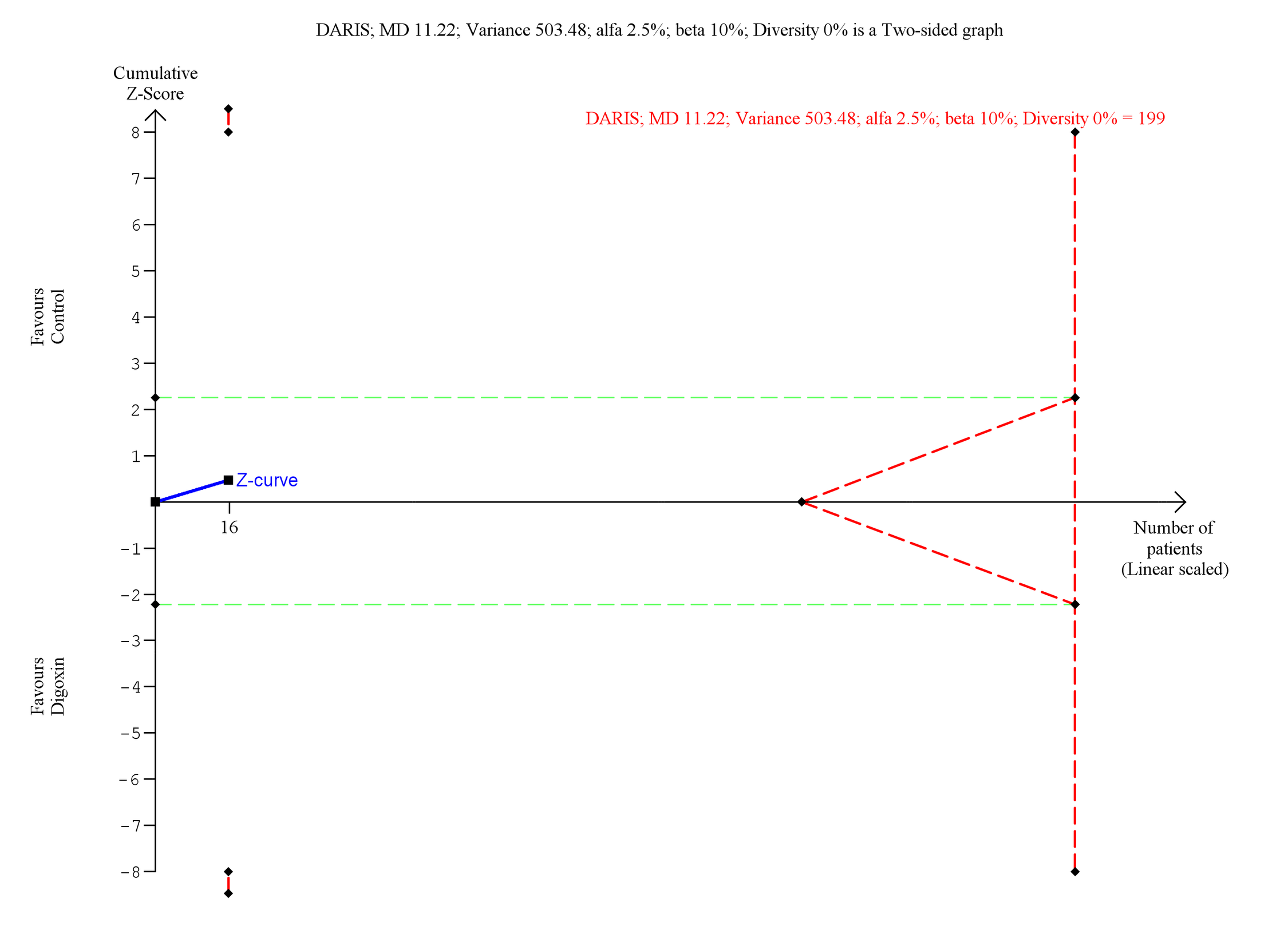

Supplement: S32 Fig — (TIF) [file pone.0193924.s035.tif]

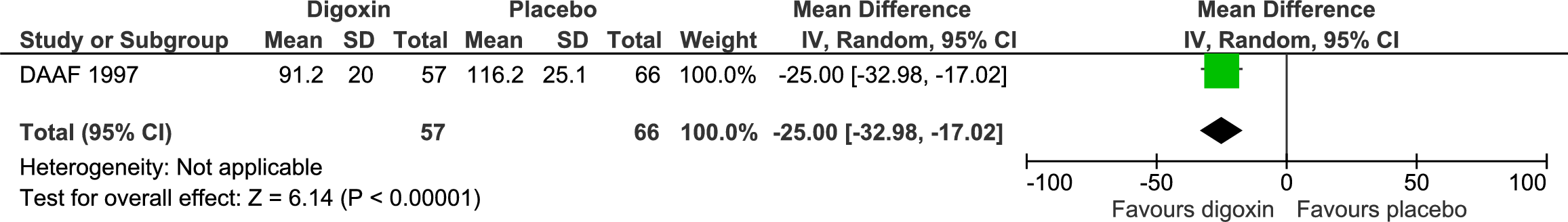

Supplement: S33 Fig — (TIF) [file pone.0193924.s036.tif]

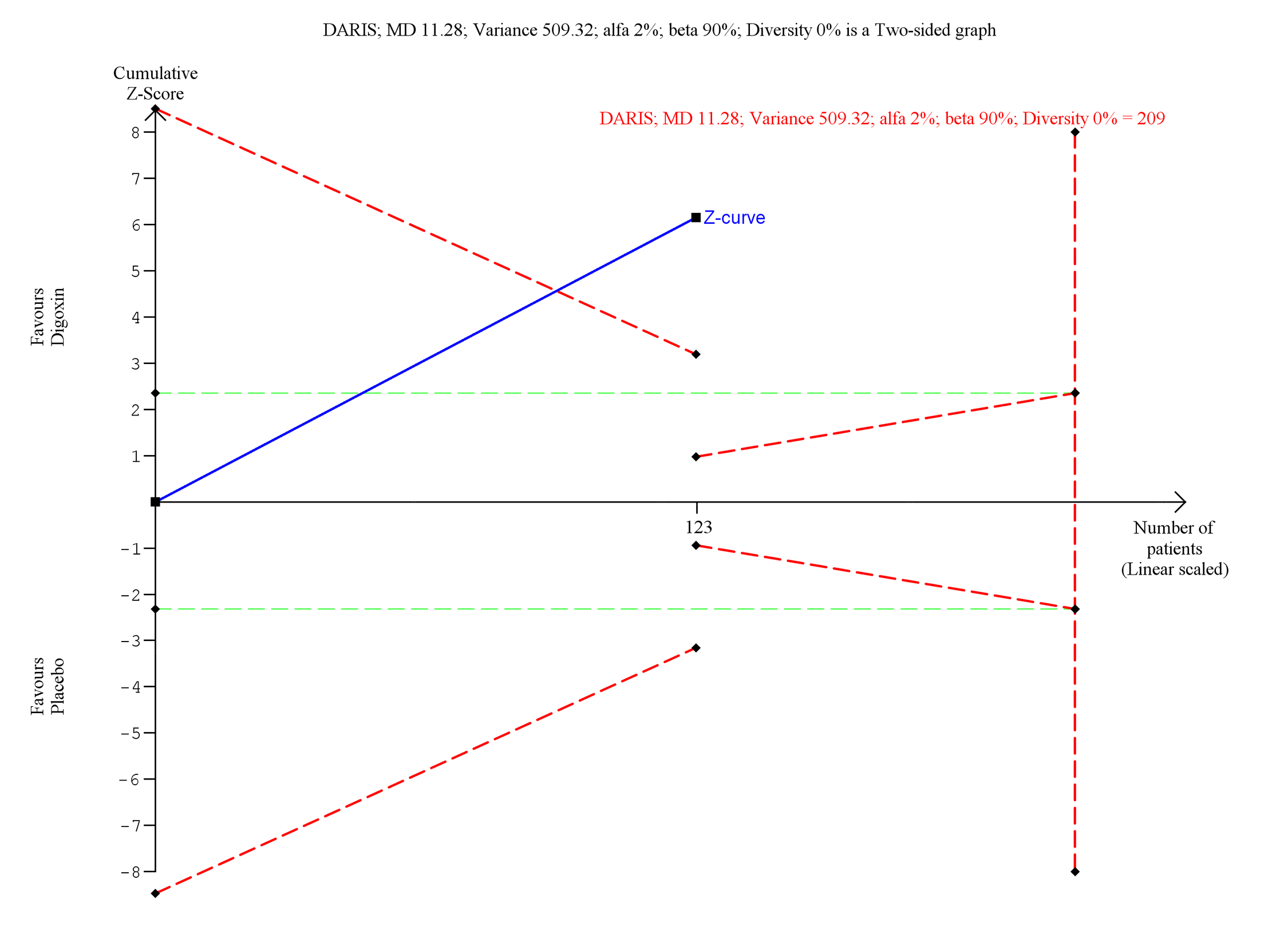

Supplement: S34 Fig — (TIF) [file pone.0193924.s037.tif]

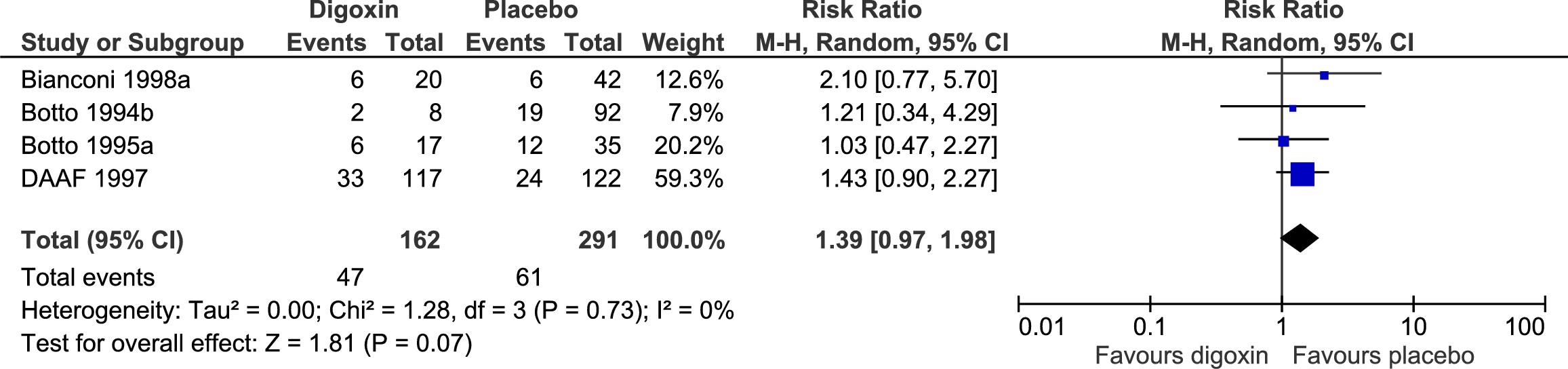

Supplement: S35 Fig — (TIF) [file pone.0193924.s038.tif]

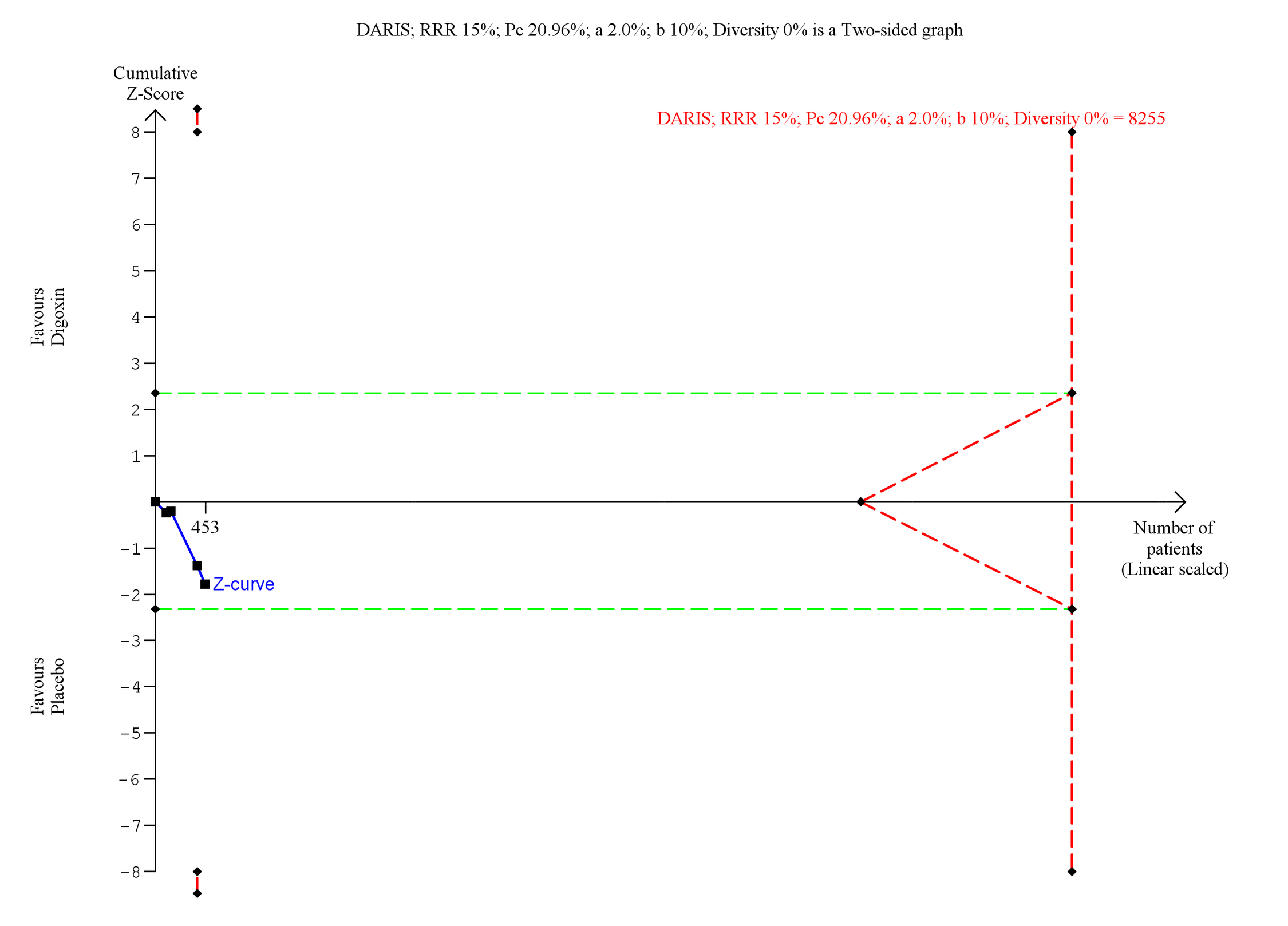

Supplement: S36 Fig — (TIF) [file pone.0193924.s039.tif]

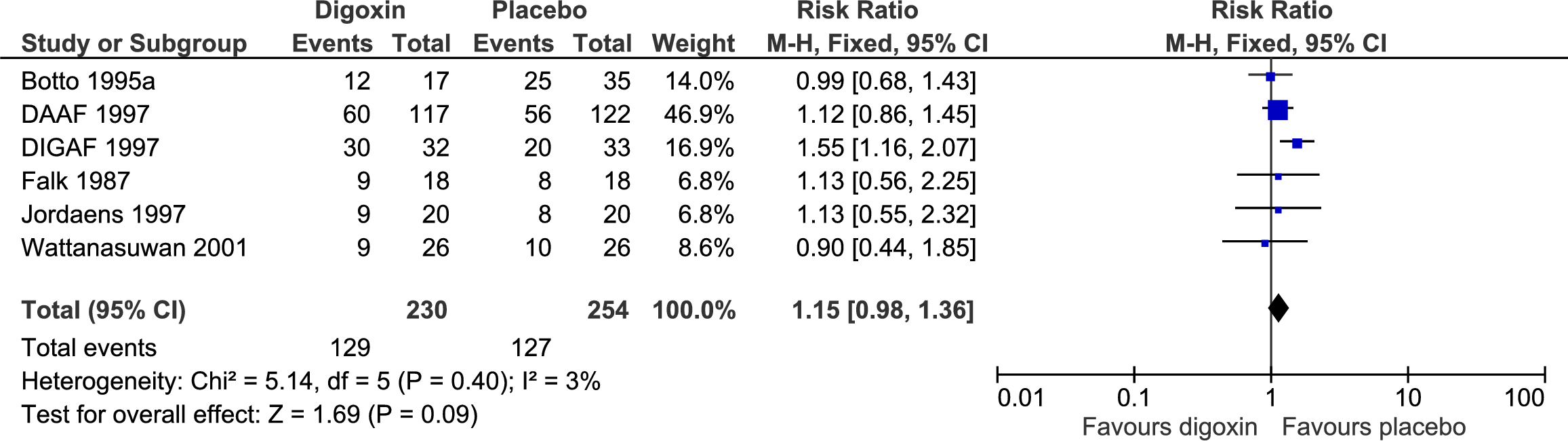

Supplement: S37 Fig — (TIF) [file pone.0193924.s040.tif]

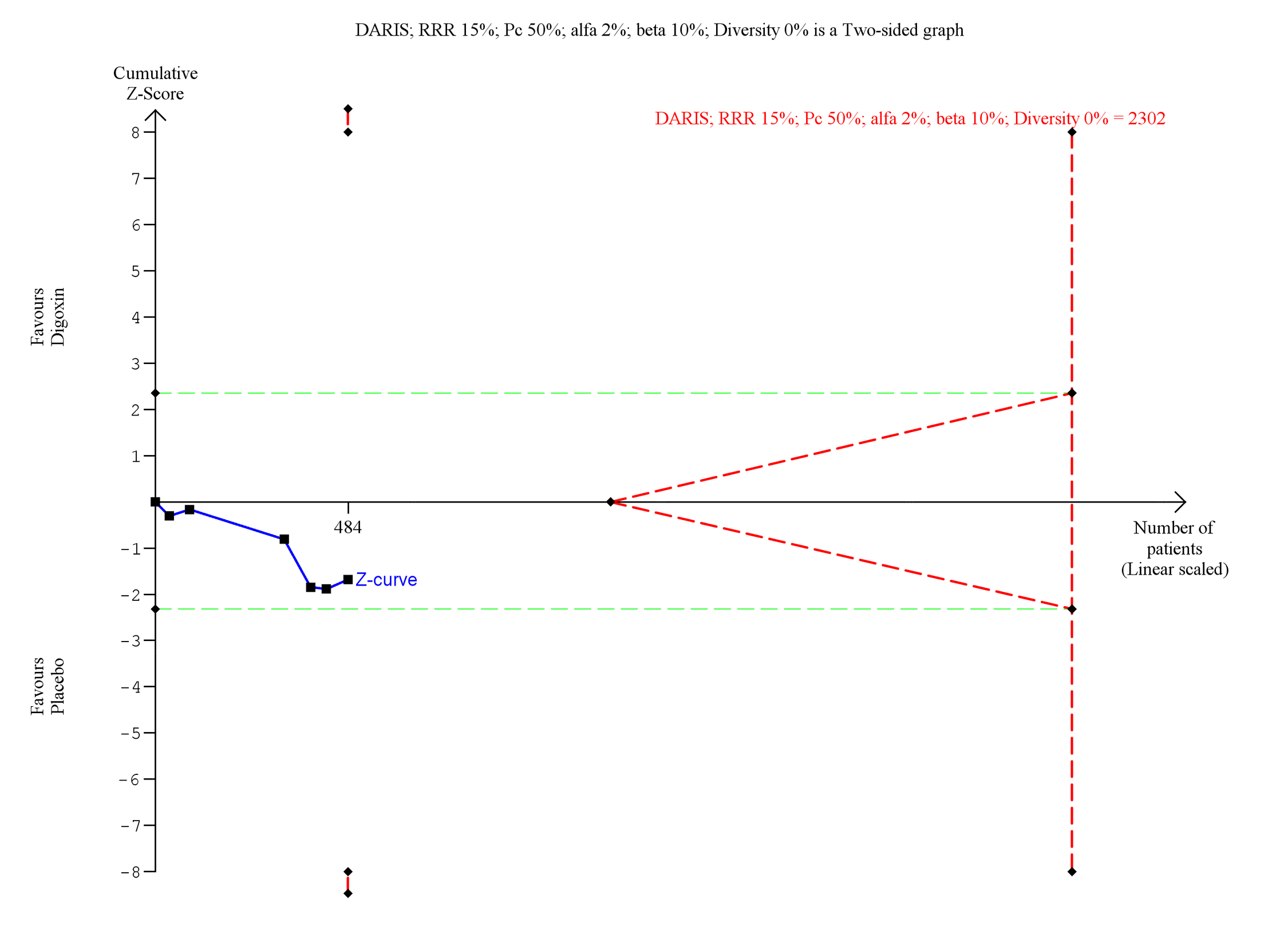

Supplement: S38 Fig — (TIF) [file pone.0193924.s041.tif]

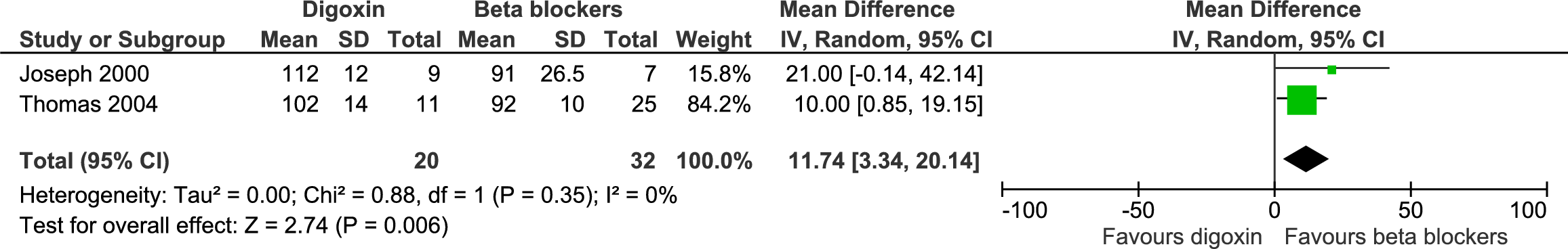

Supplement: S39 Fig — (TIF) [file pone.0193924.s042.tif]

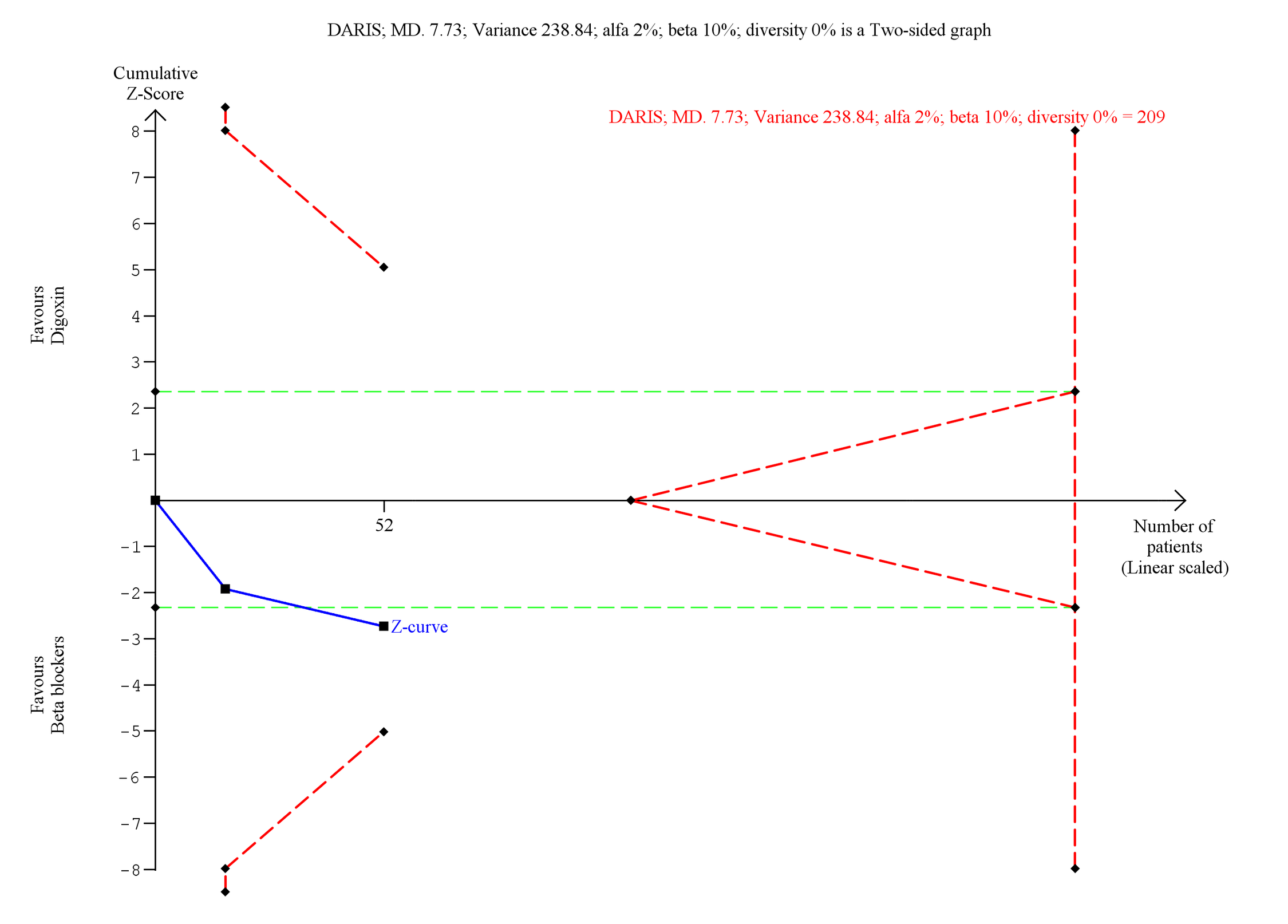

Supplement: S40 Fig — (TIF) [file pone.0193924.s043.tif]

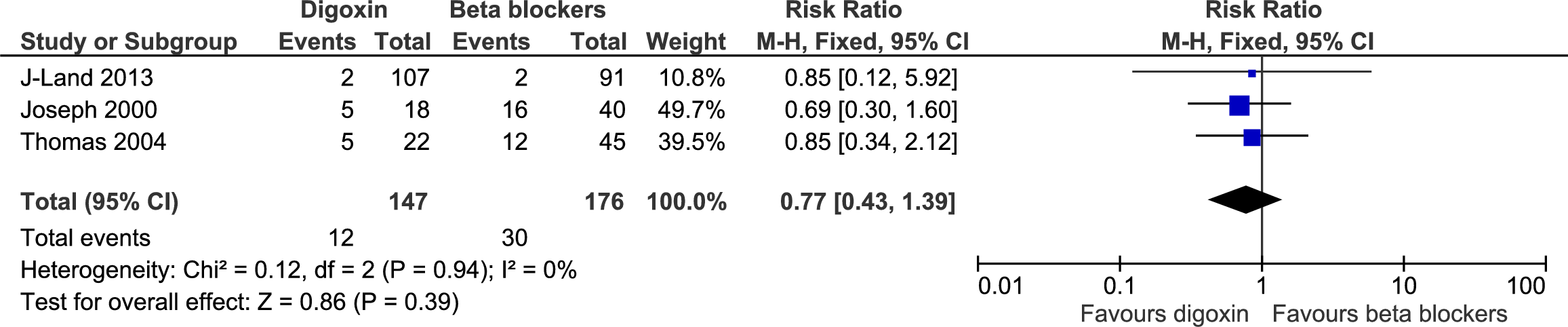

Supplement: S41 Fig — (TIF) [file pone.0193924.s044.tif]

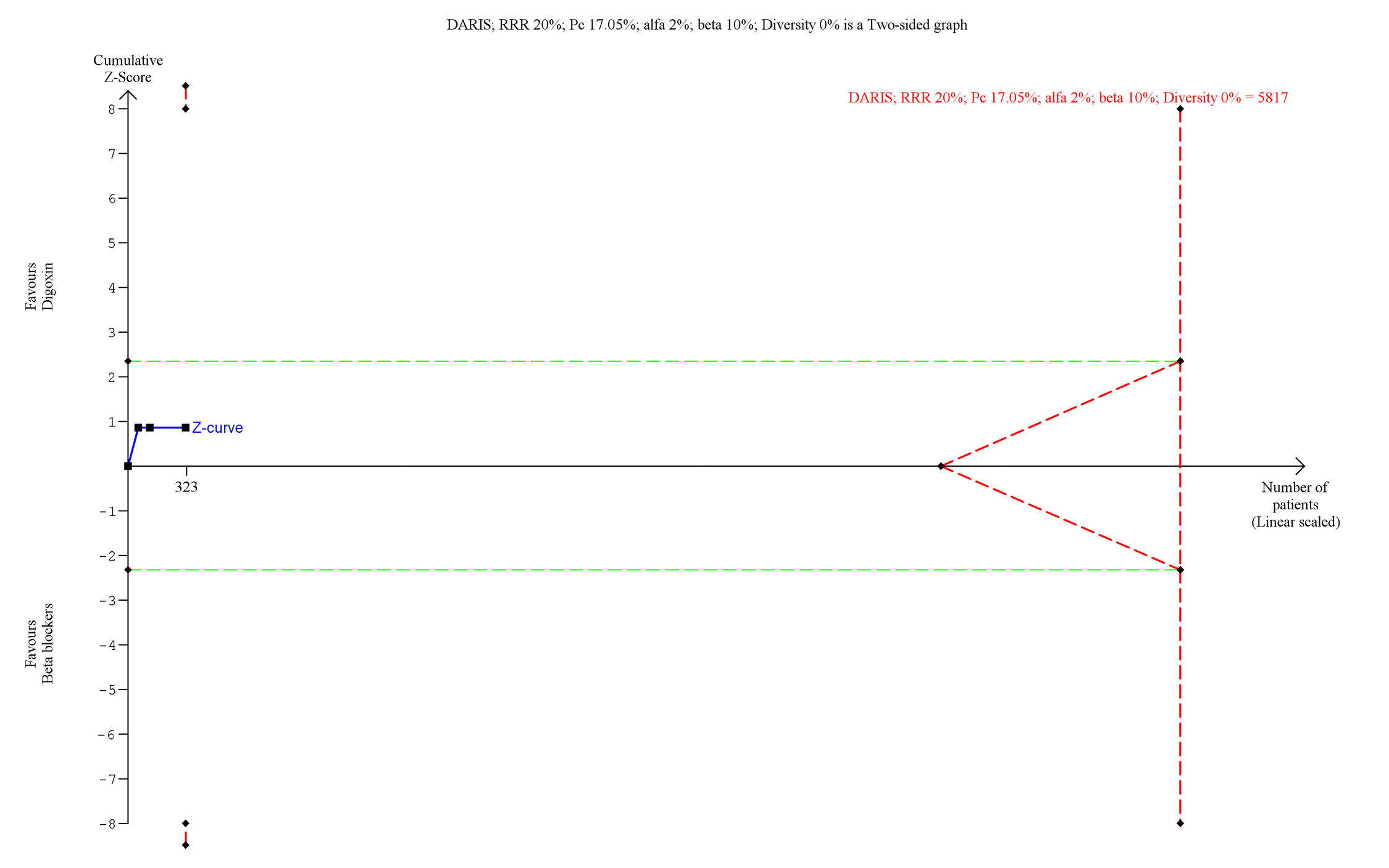

Supplement: S42 Fig — (TIF) [file pone.0193924.s045.tif]

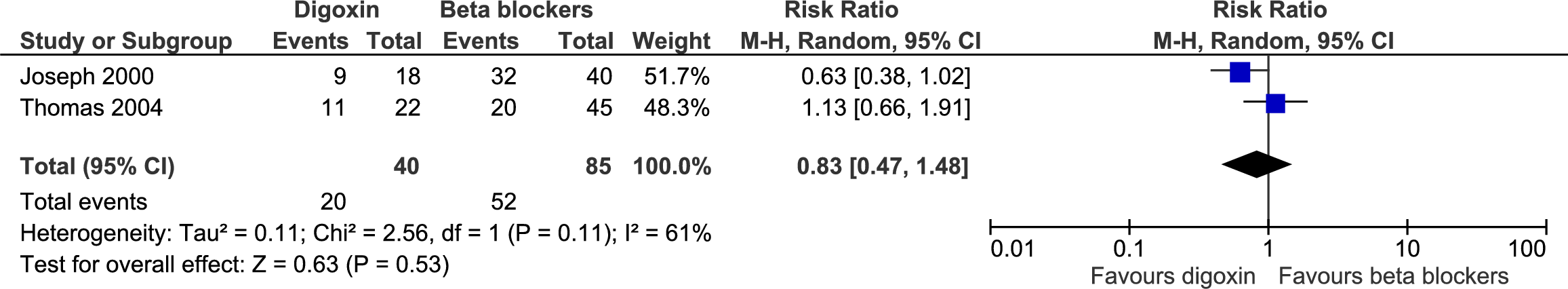

Supplement: S43 Fig — (TIF) [file pone.0193924.s046.tif]

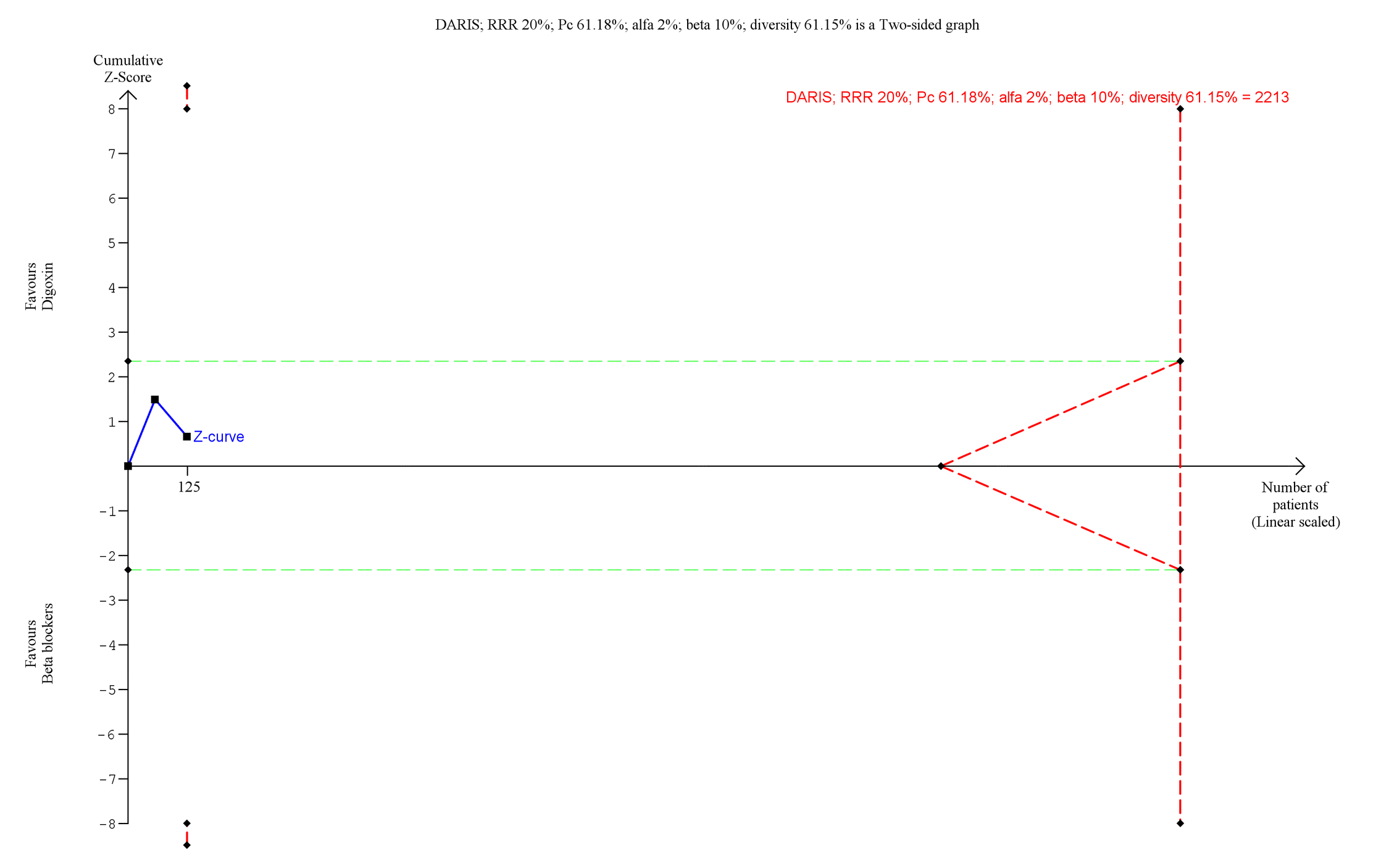

Supplement: S44 Fig — (TIF) [file pone.0193924.s047.tif]

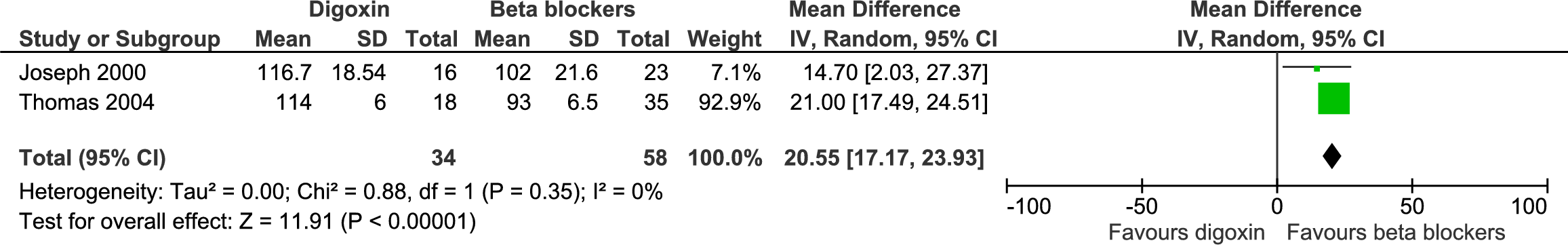

Supplement: S45 Fig — (TIF) [file pone.0193924.s048.tif]

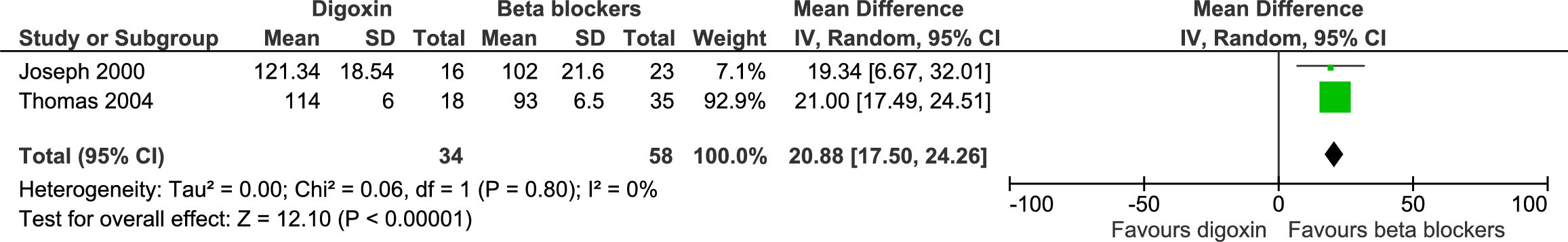

Supplement: S46 Fig — (TIF) [file pone.0193924.s049.tif]

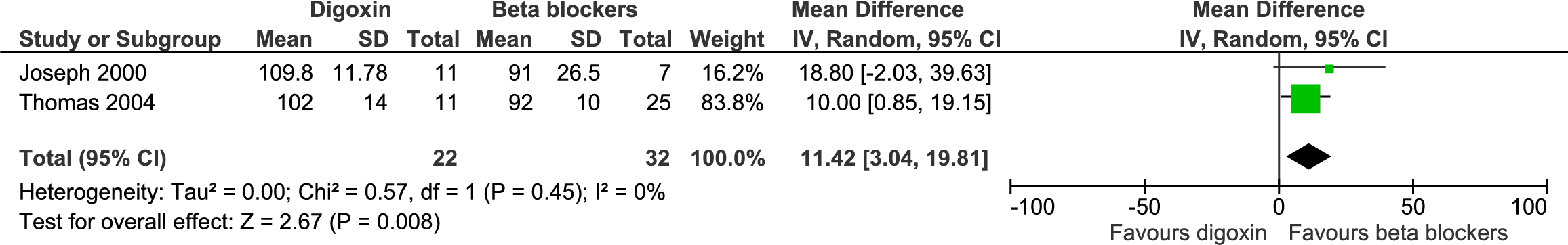

Supplement: S47 Fig — (TIF) [file pone.0193924.s050.tif]

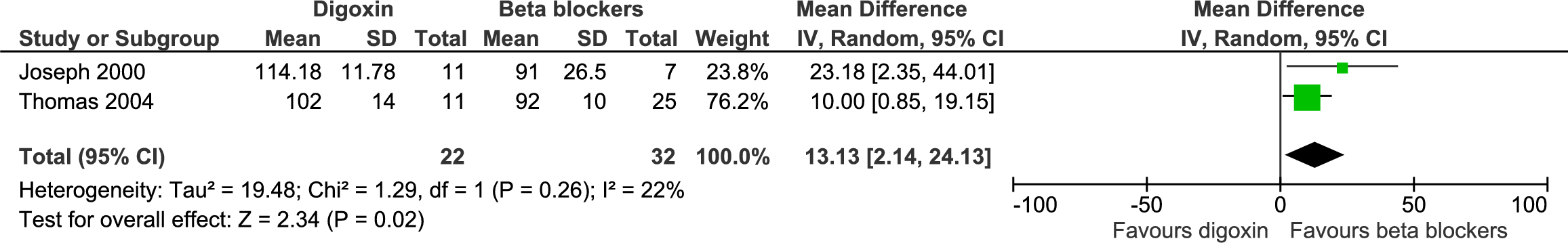

Supplement: S48 Fig — (TIF) [file pone.0193924.s051.tif]

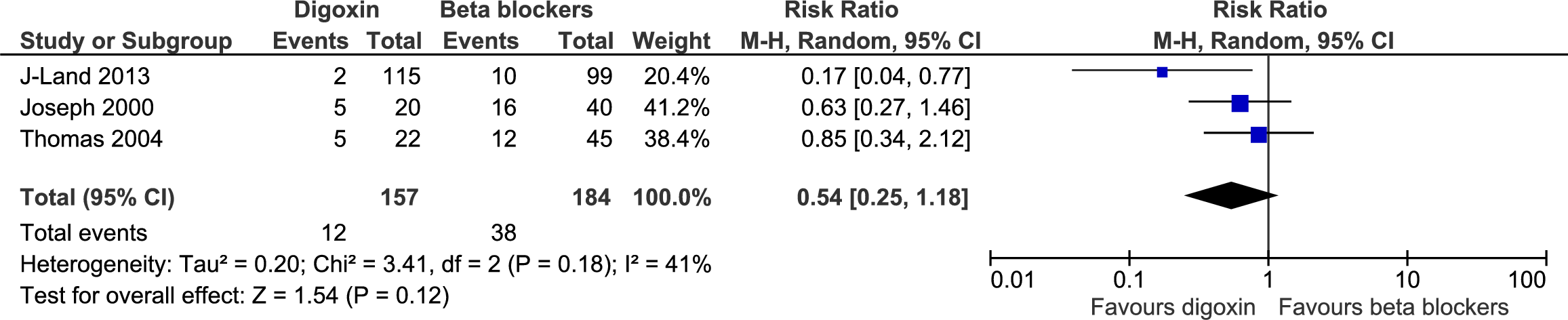

Supplement: S49 Fig — (TIF) [file pone.0193924.s052.tif]

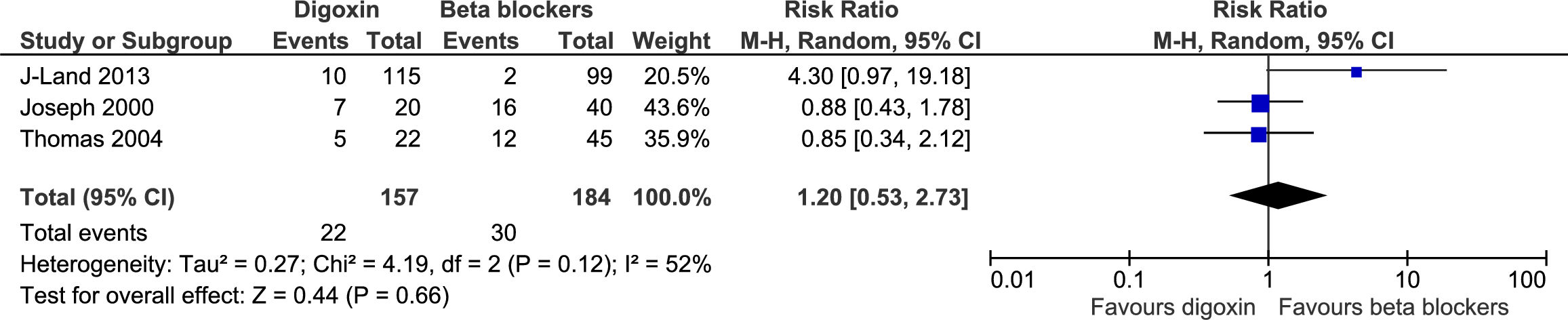

Supplement: S50 Fig — (TIF) [file pone.0193924.s053.tif]

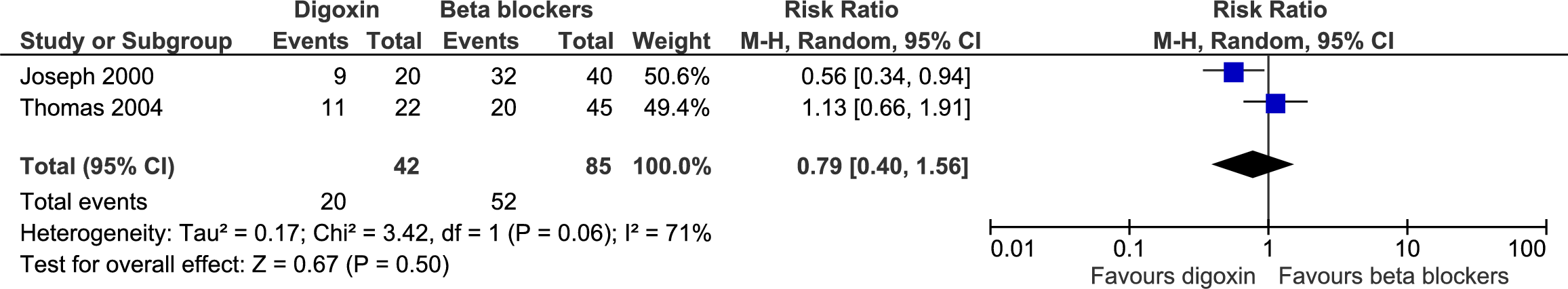

Supplement: S51 Fig — (TIF) [file pone.0193924.s054.tif]

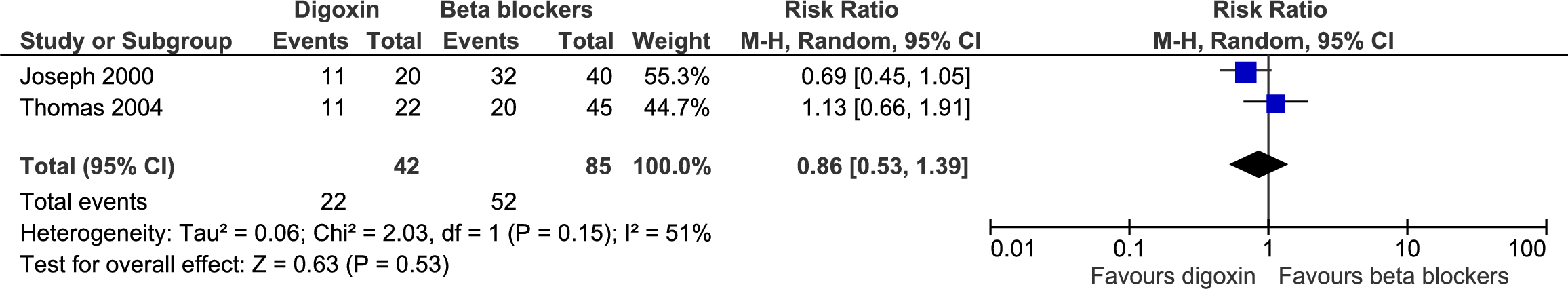

Supplement: S52 Fig — (TIF) [file pone.0193924.s055.tif]

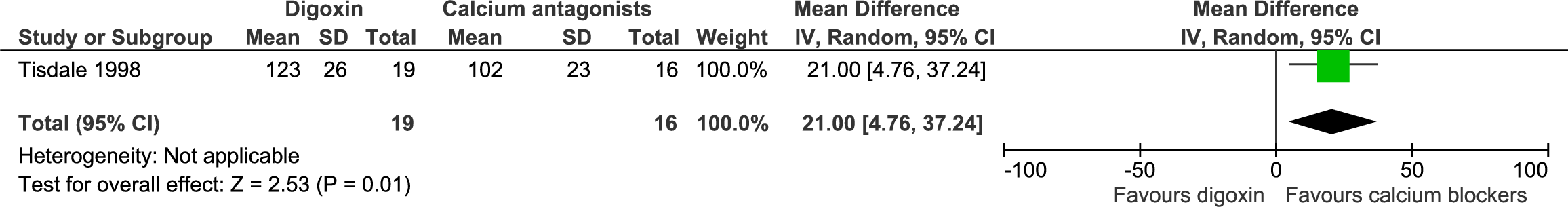

Supplement: S53 Fig — (TIF) [file pone.0193924.s056.tif]

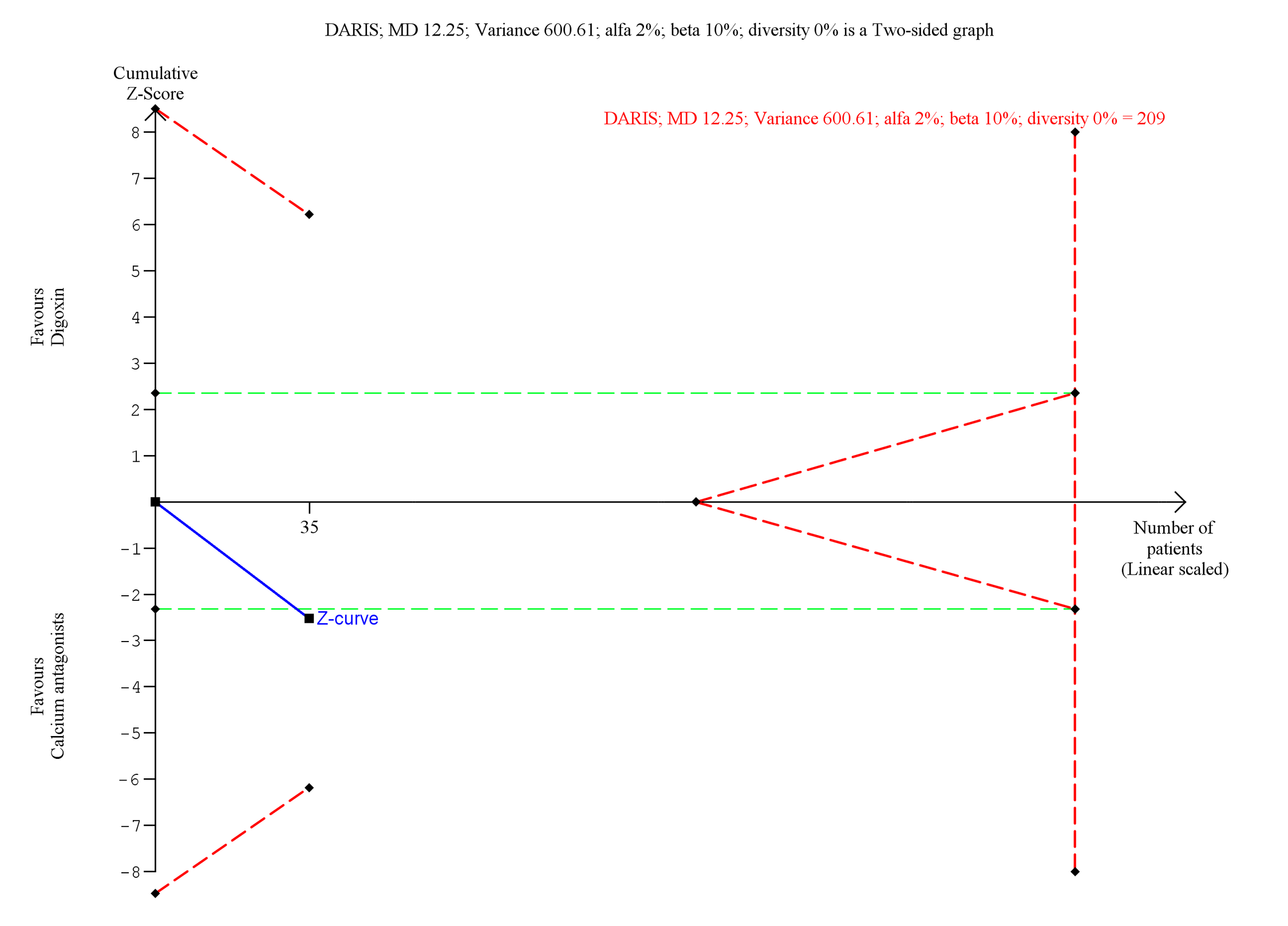

Supplement: S54 Fig — (TIF) [file pone.0193924.s057.tif]

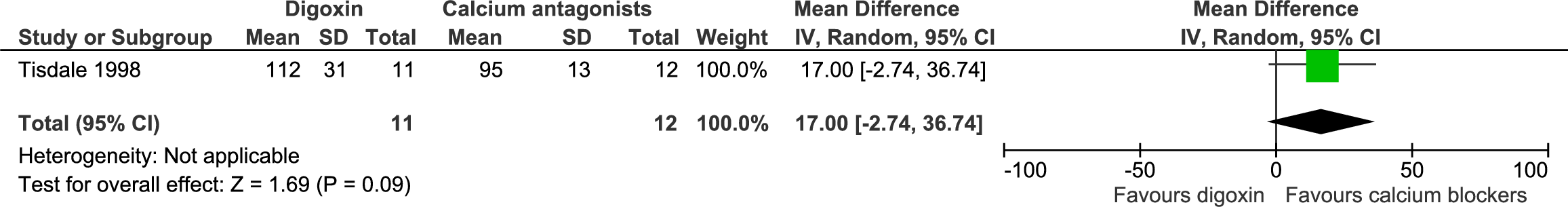

Supplement: S55 Fig — (TIF) [file pone.0193924.s058.tif]

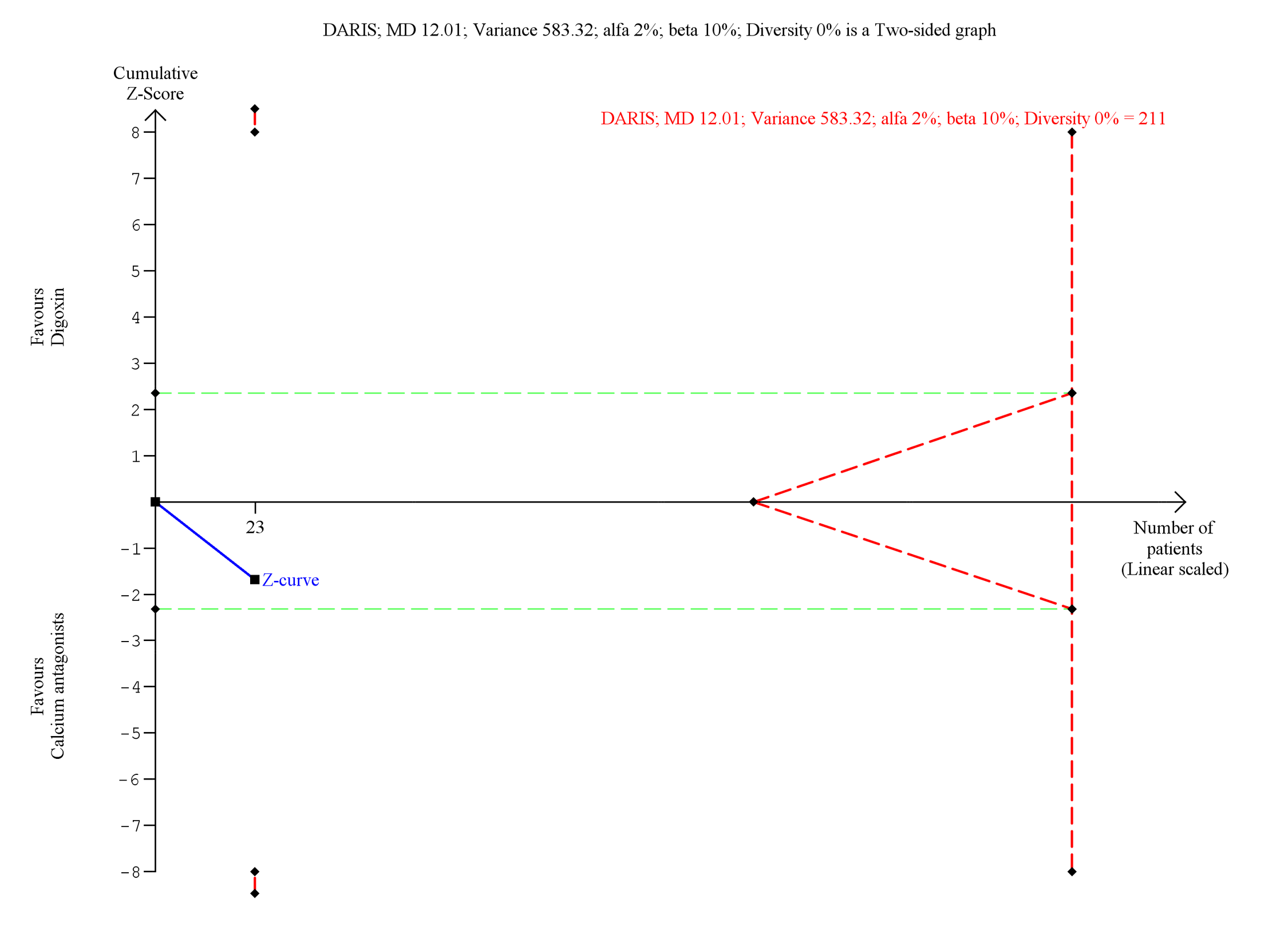

Supplement: S56 Fig — (TIF) [file pone.0193924.s059.tif]

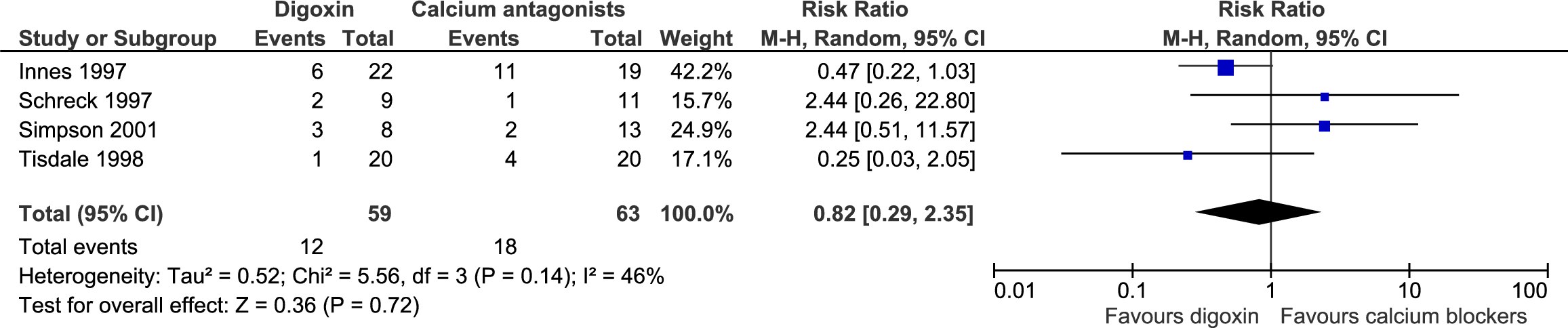

Supplement: S57 Fig — (TIF) [file pone.0193924.s060.tif]

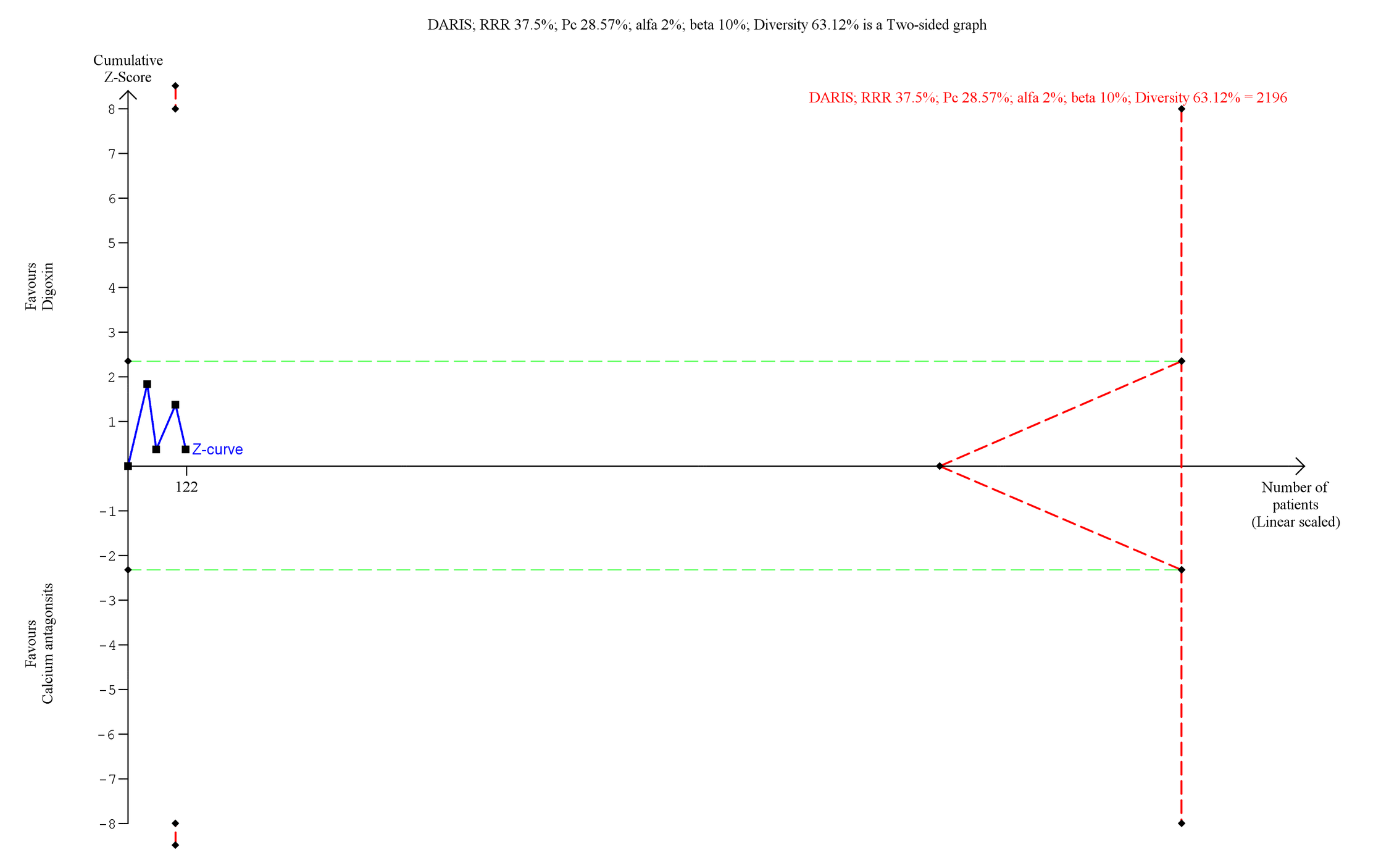

Supplement: S58 Fig — (TIF) [file pone.0193924.s061.tif]

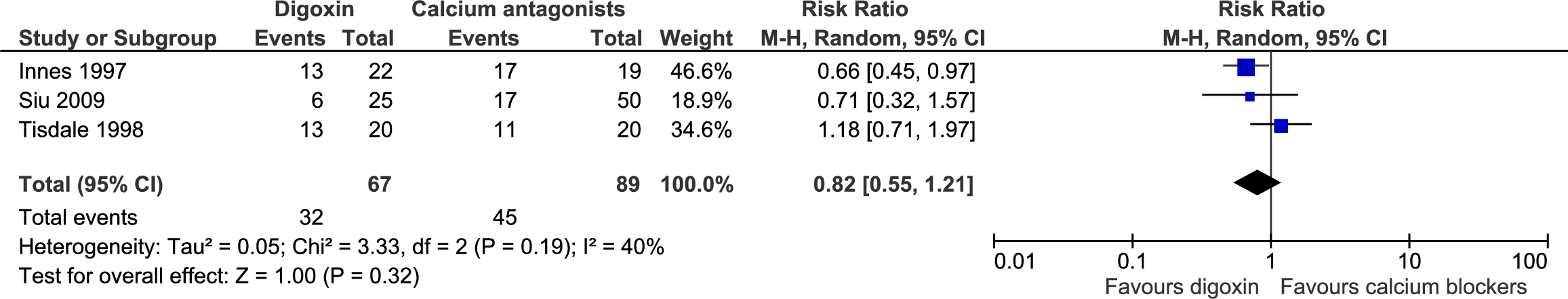

Supplement: S59 Fig — (TIF) [file pone.0193924.s062.tif]

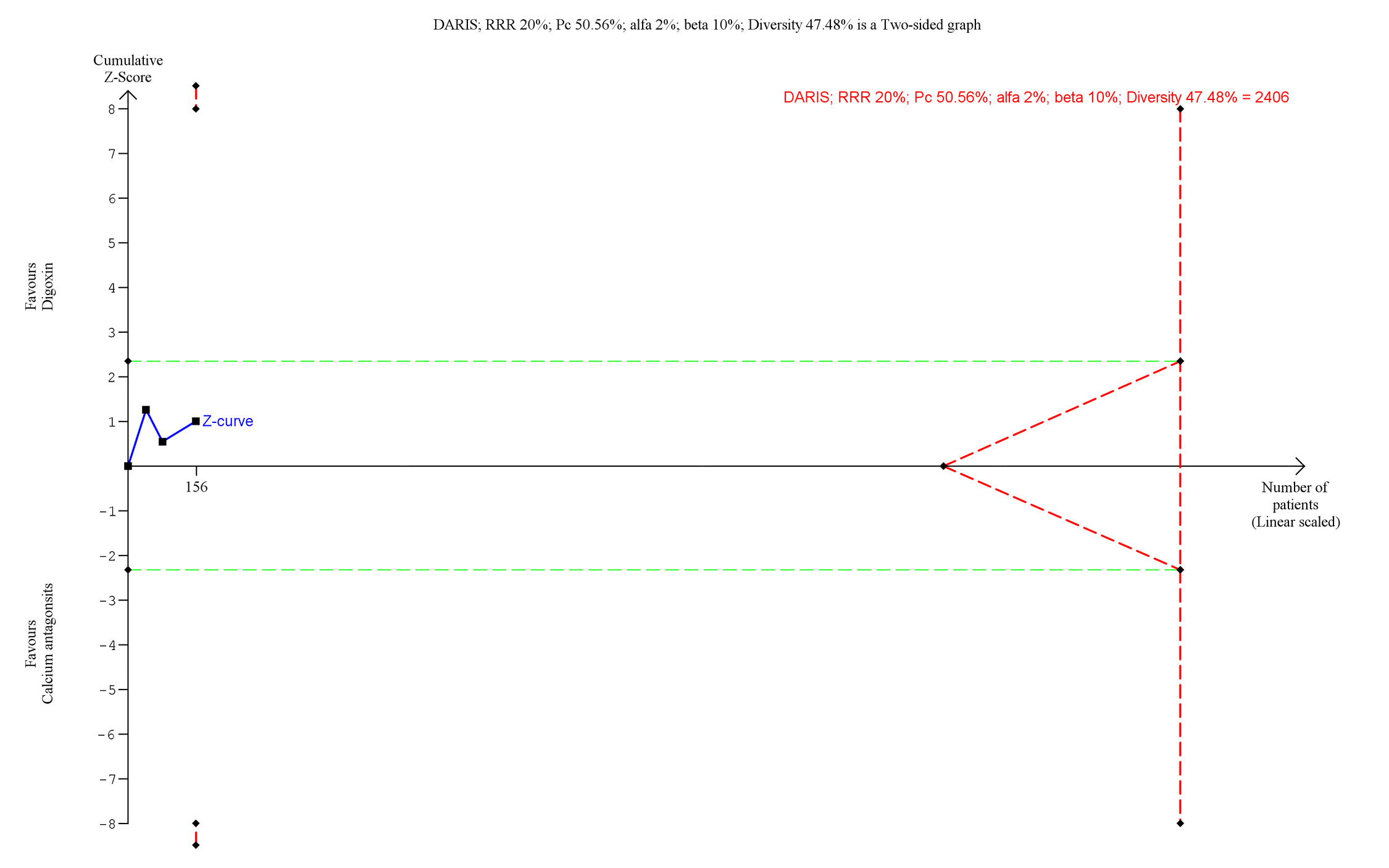

Supplement: S60 Fig — (TIF) [file pone.0193924.s063.tif]

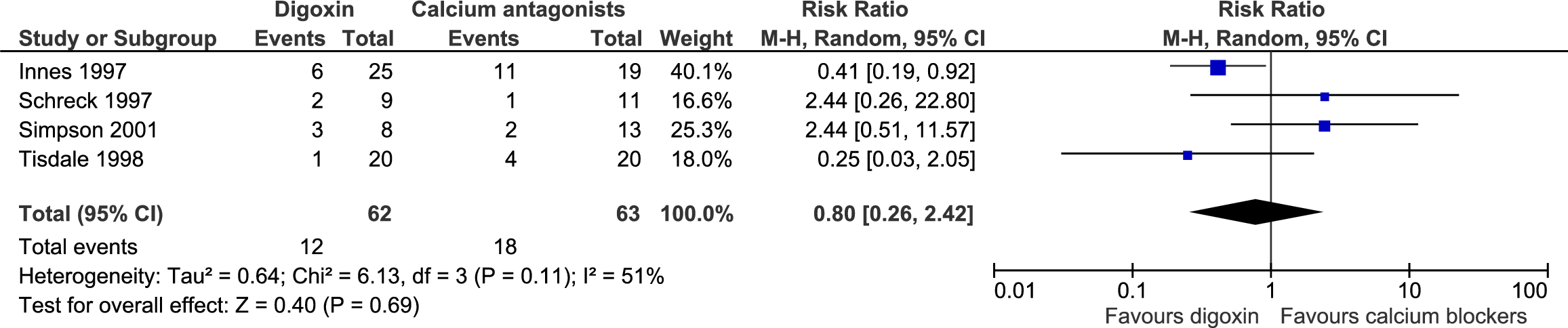

Supplement: S61 Fig — (TIF) [file pone.0193924.s064.tif]

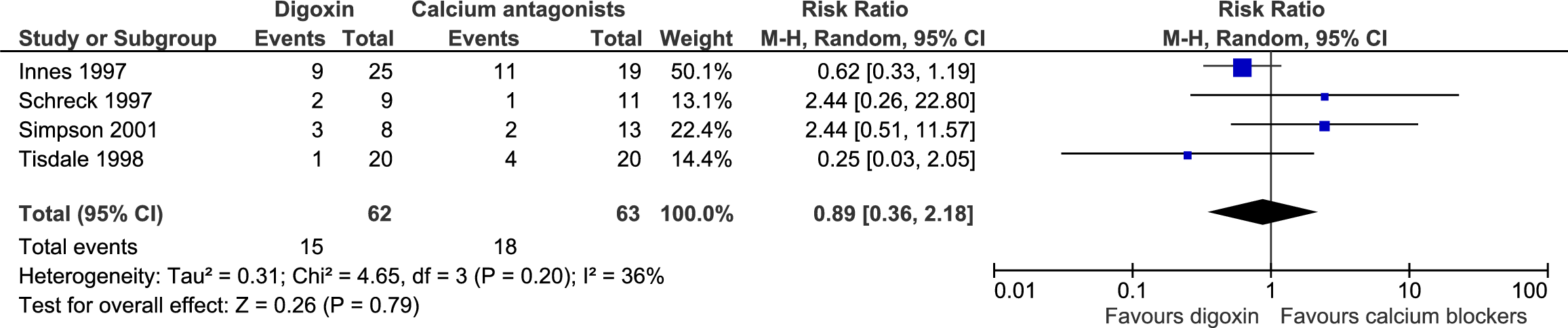

Supplement: S62 Fig — (TIF) [file pone.0193924.s065.tif]

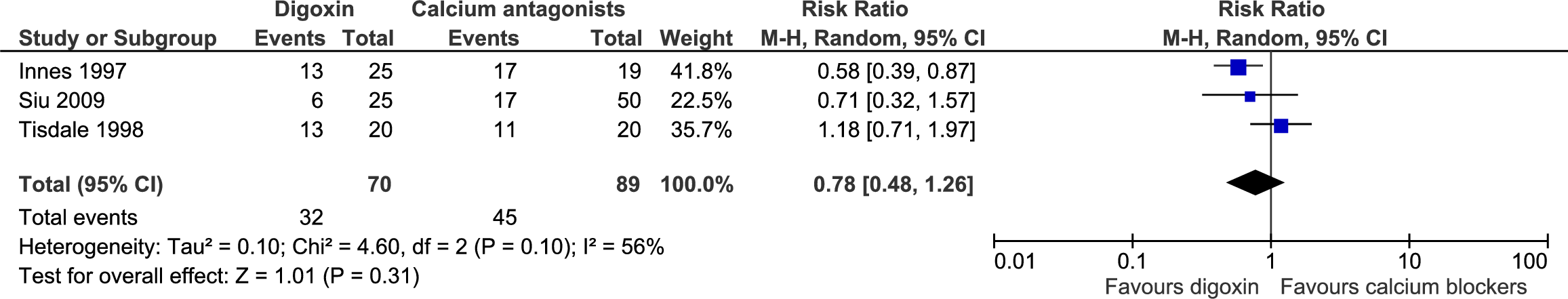

Supplement: S63 Fig — (TIF) [file pone.0193924.s066.tif]

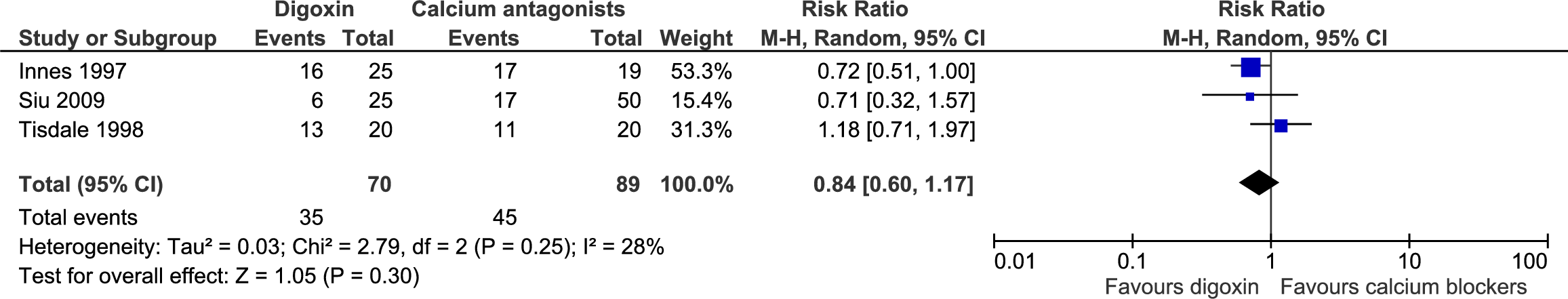

Supplement: S64 Fig — (TIF) [file pone.0193924.s067.tif]

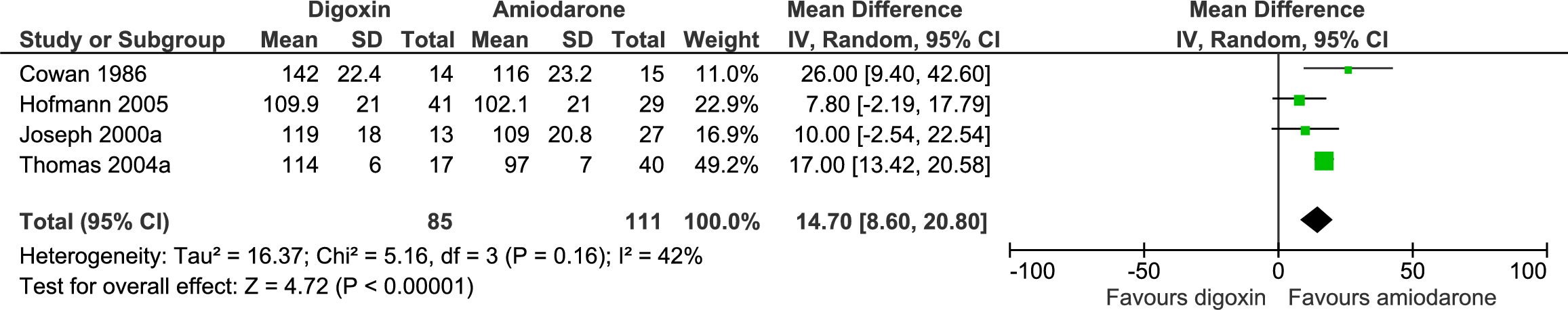

Supplement: S65 Fig — (TIF) [file pone.0193924.s068.tif]

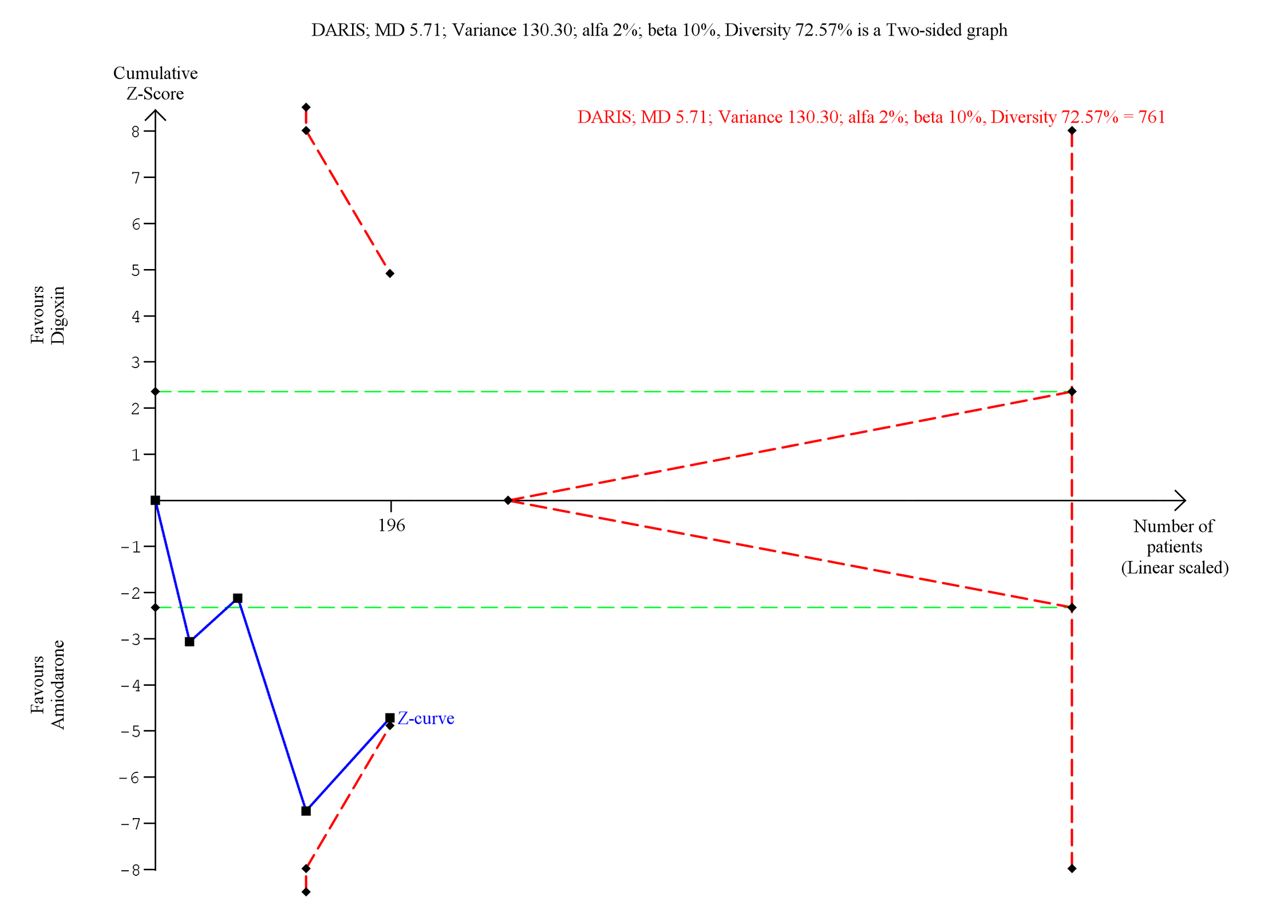

Supplement: S66 Fig — (TIF) [file pone.0193924.s069.tif]

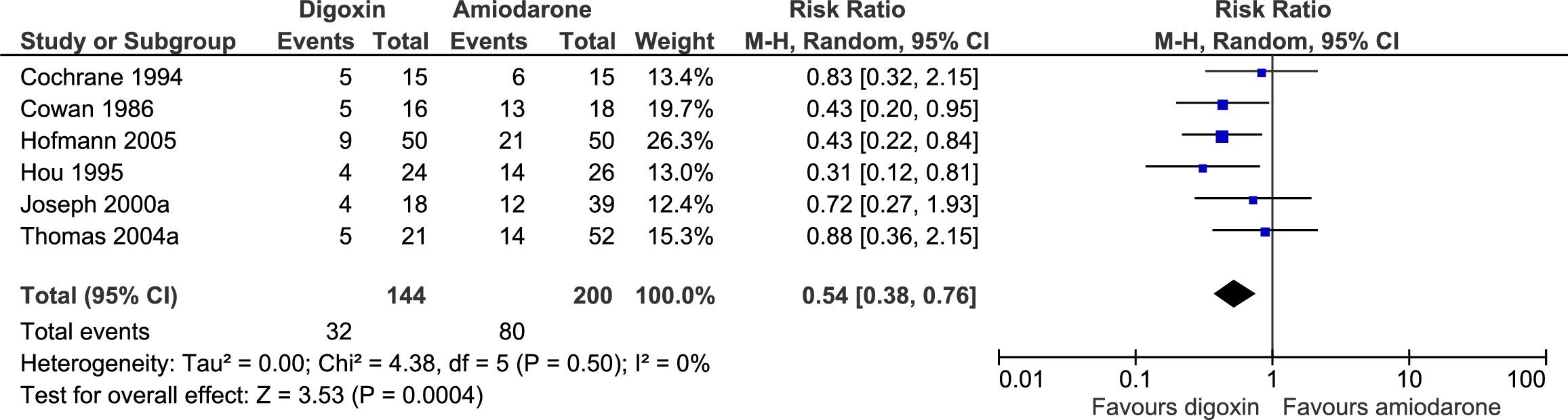

Supplement: S67 Fig — (TIF) [file pone.0193924.s070.tif]

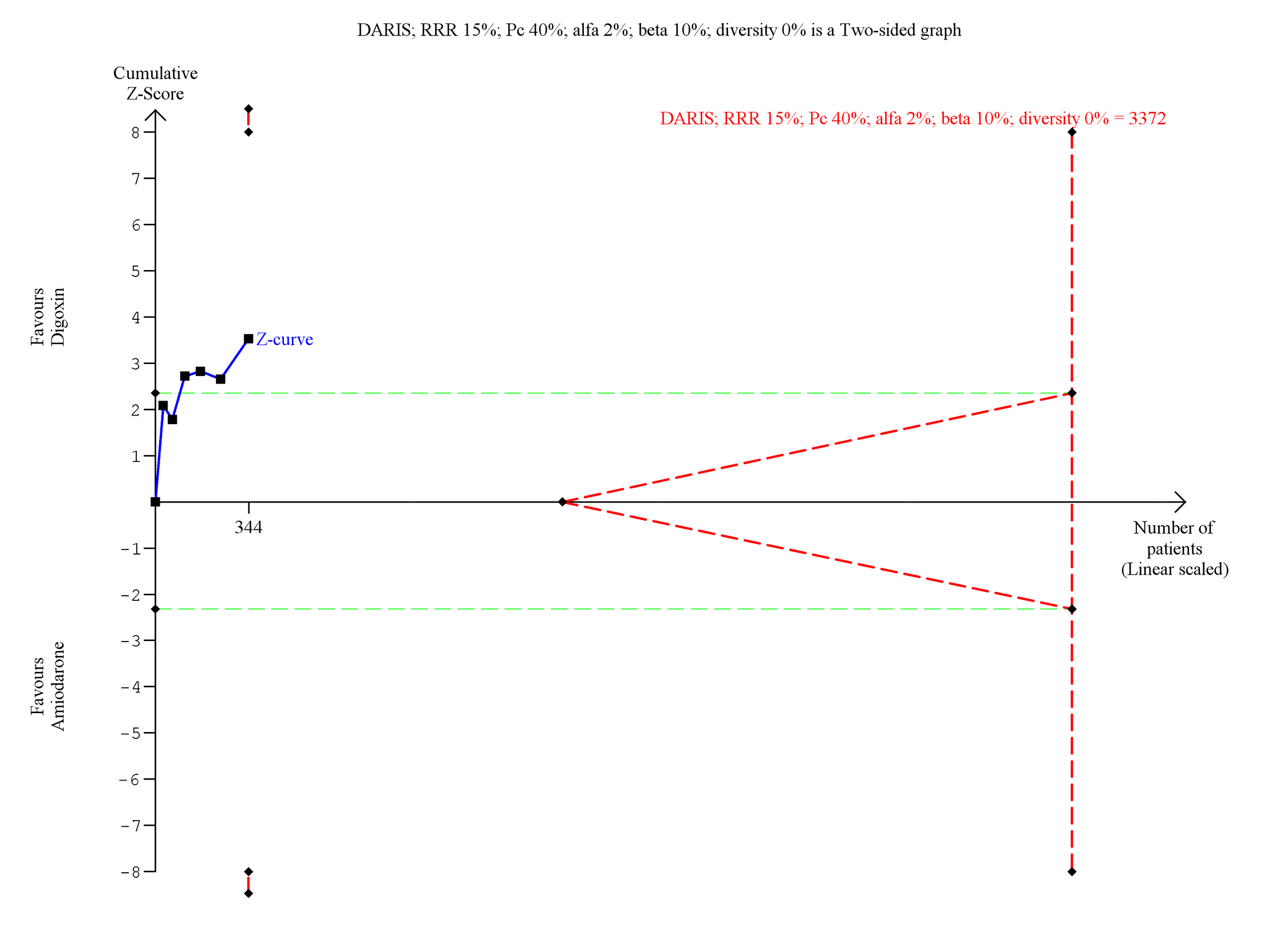

Supplement: S68 Fig — (TIF) [file pone.0193924.s071.tif]

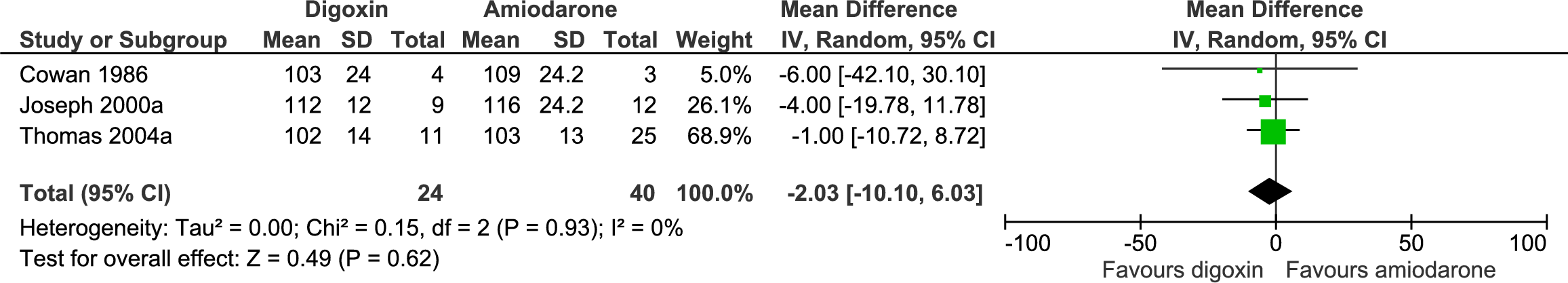

Supplement: S69 Fig — (TIF) [file pone.0193924.s072.tif]

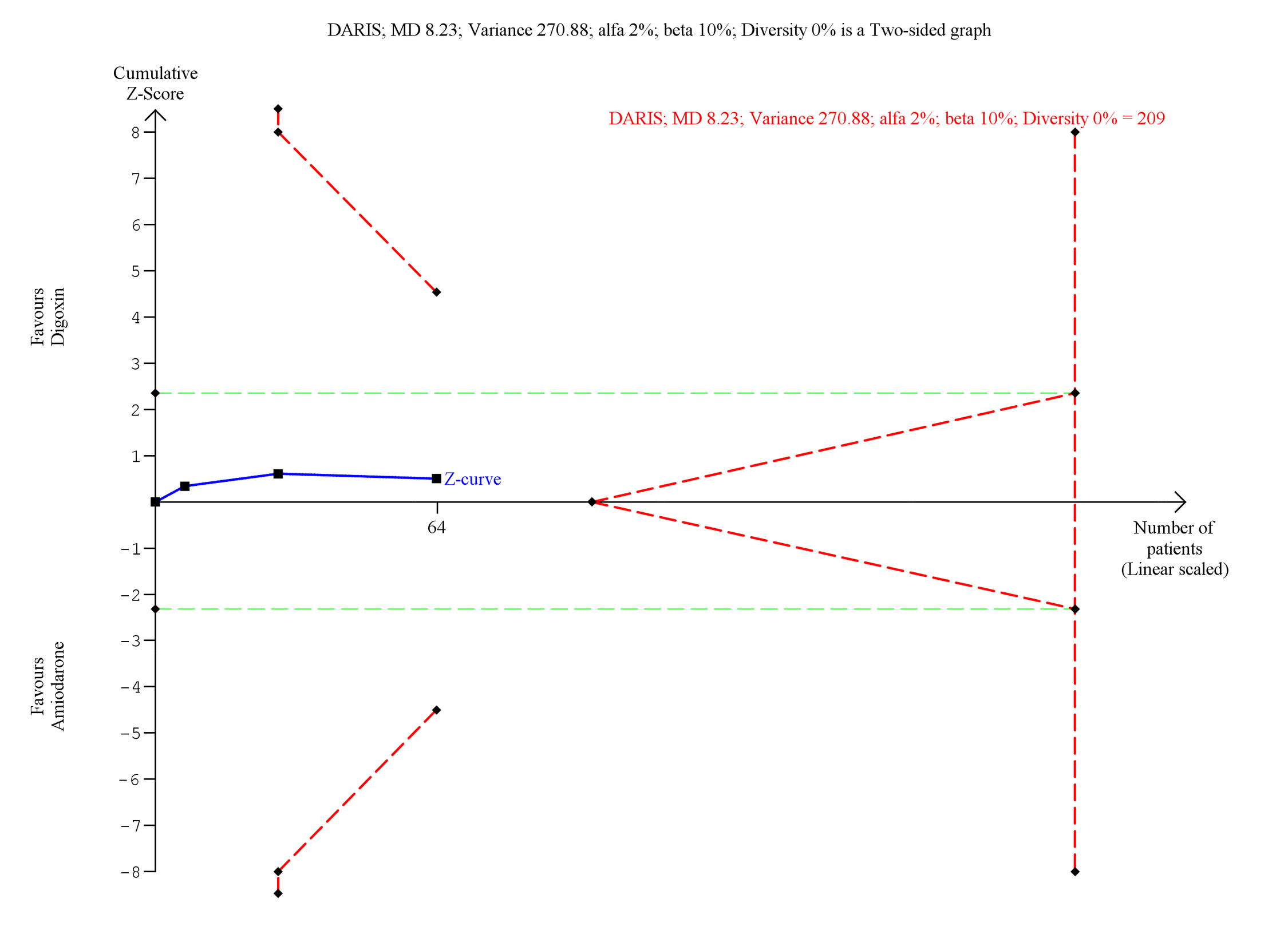

Supplement: S70 Fig — (TIF) [file pone.0193924.s073.tif]

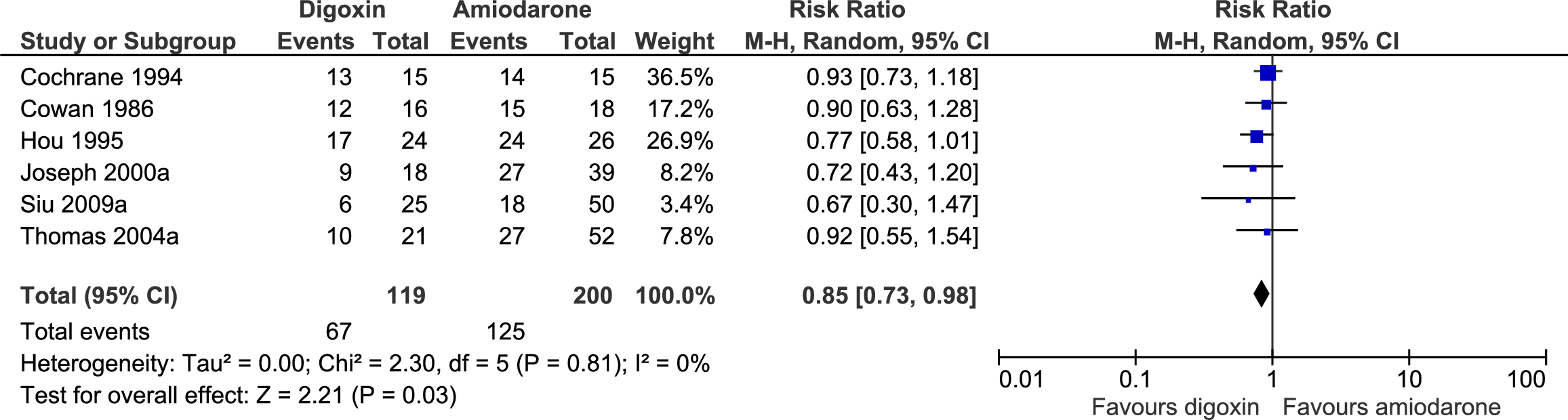

Supplement: S71 Fig — (TIF) [file pone.0193924.s074.tif]

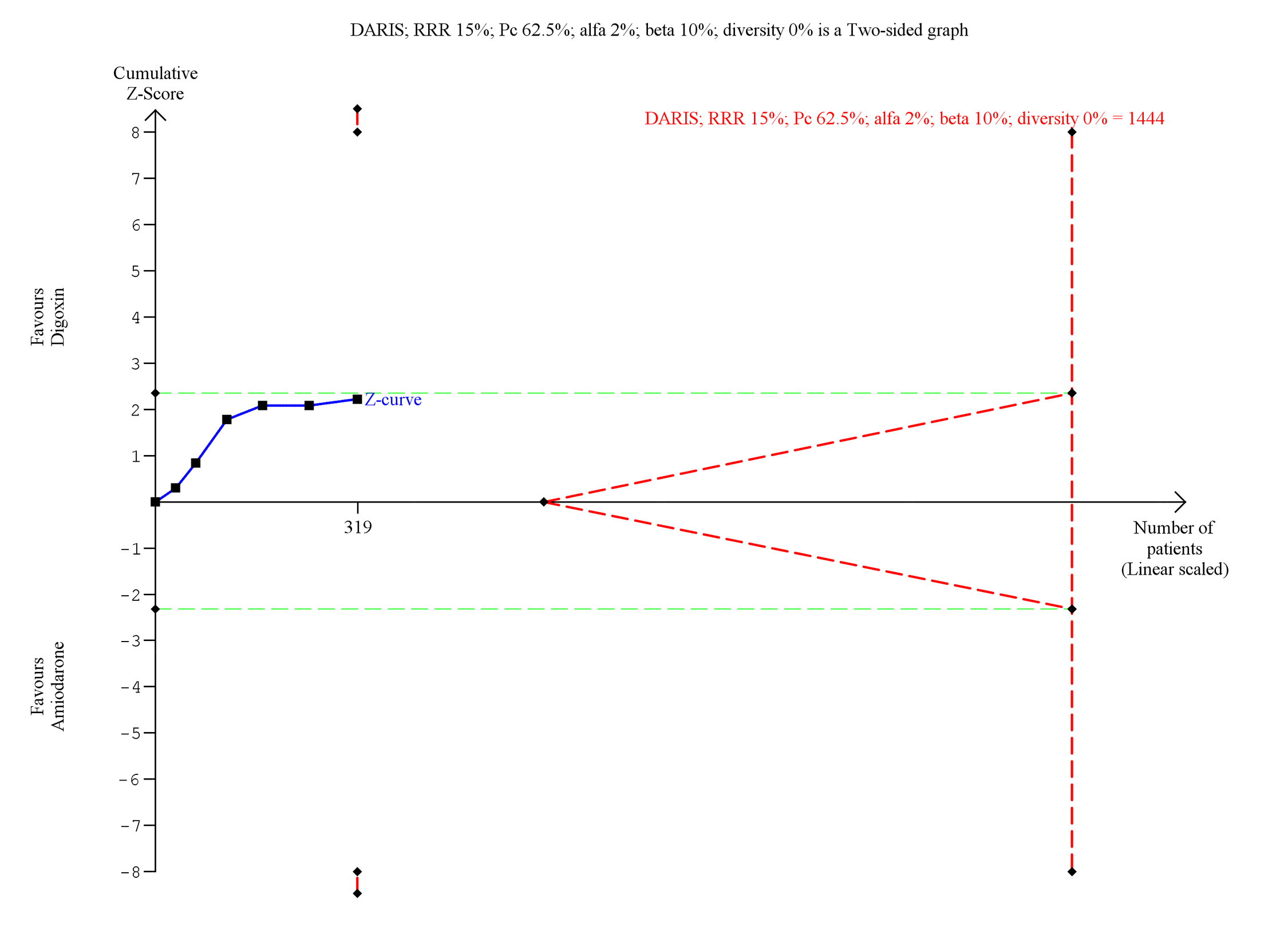

Supplement: S72 Fig — (TIF) [file pone.0193924.s075.tif]

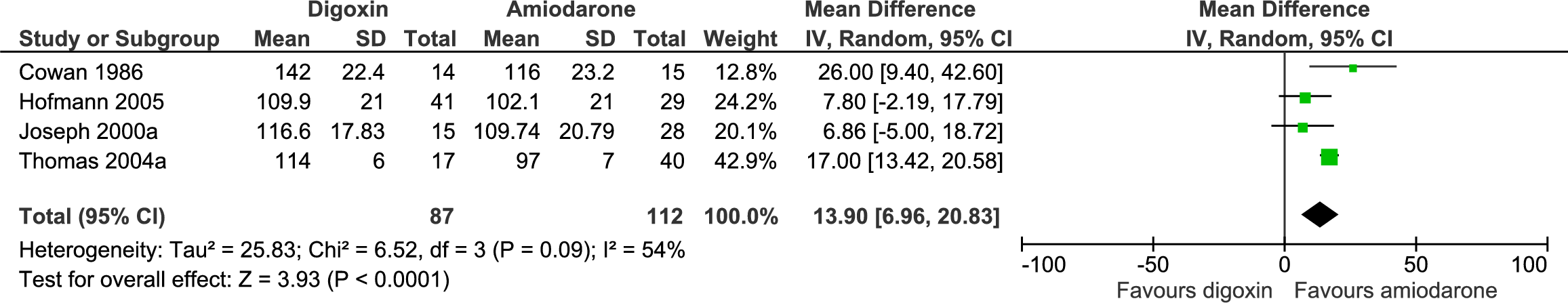

Supplement: S73 Fig — (TIF) [file pone.0193924.s076.tif]

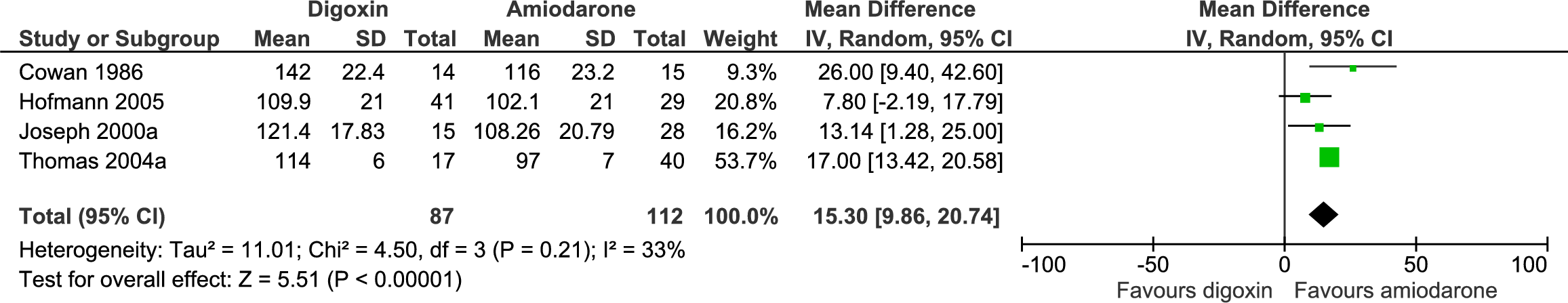

Supplement: S74 Fig — (TIF) [file pone.0193924.s077.tif]

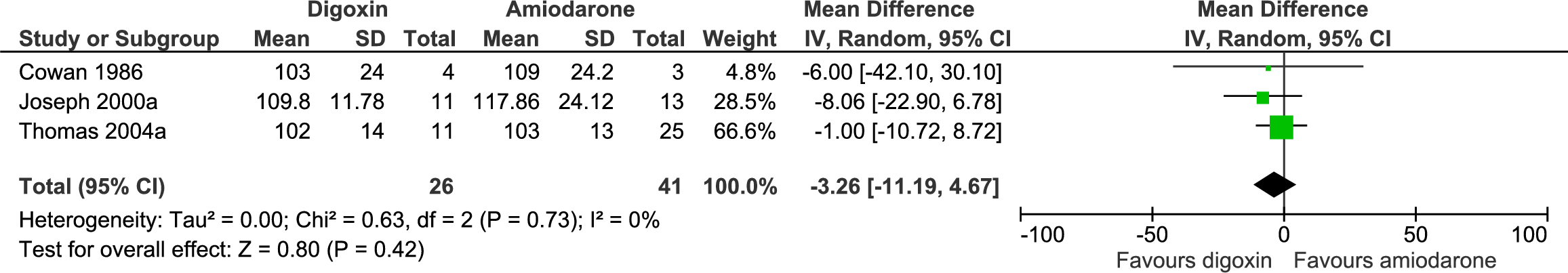

Supplement: S75 Fig — (TIF) [file pone.0193924.s078.tif]

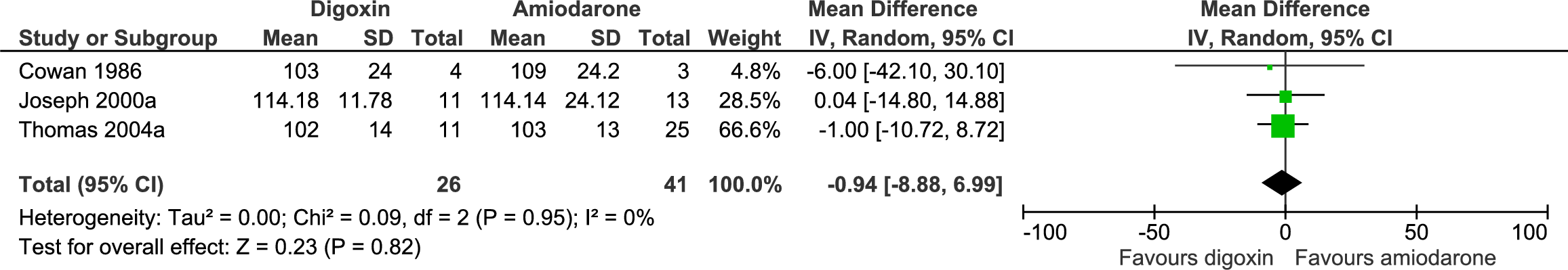

Supplement: S76 Fig — (TIF) [file pone.0193924.s079.tif]

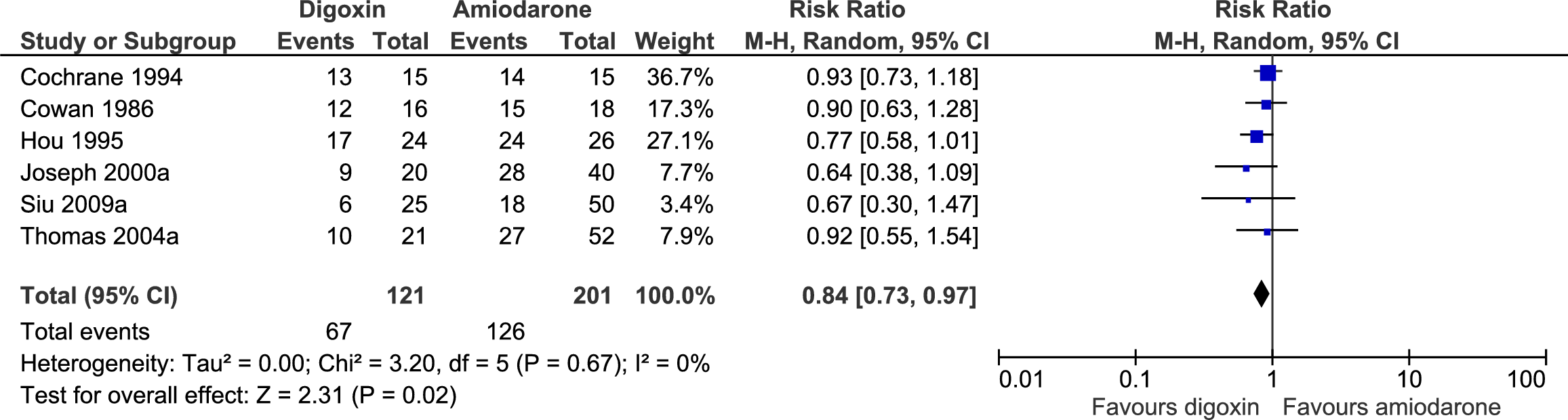

Supplement: S77 Fig — (TIF) [file pone.0193924.s080.tif]

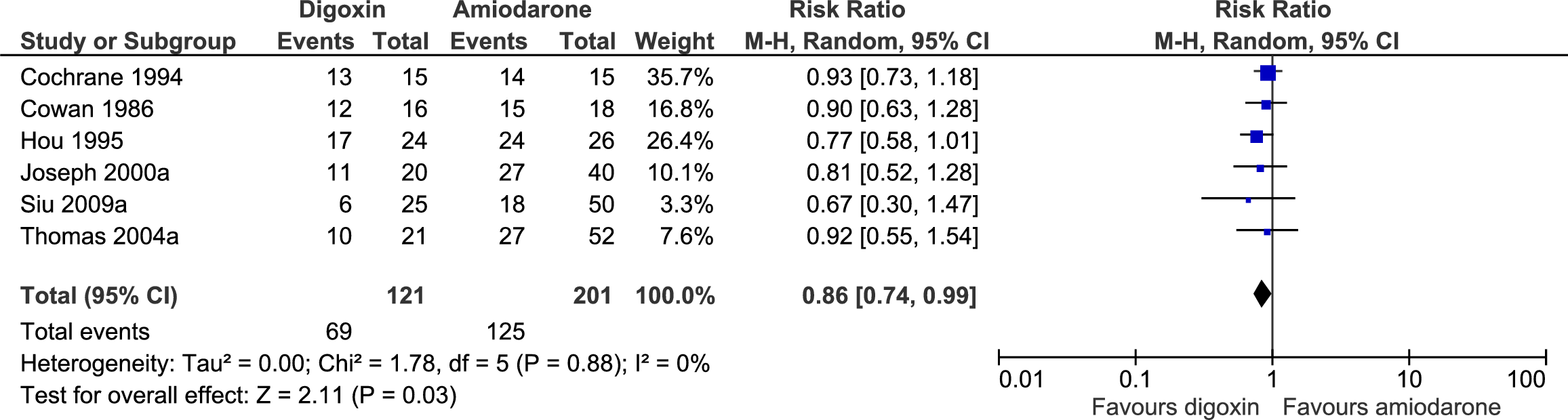

Supplement: S78 Fig — (TIF) [file pone.0193924.s081.tif]

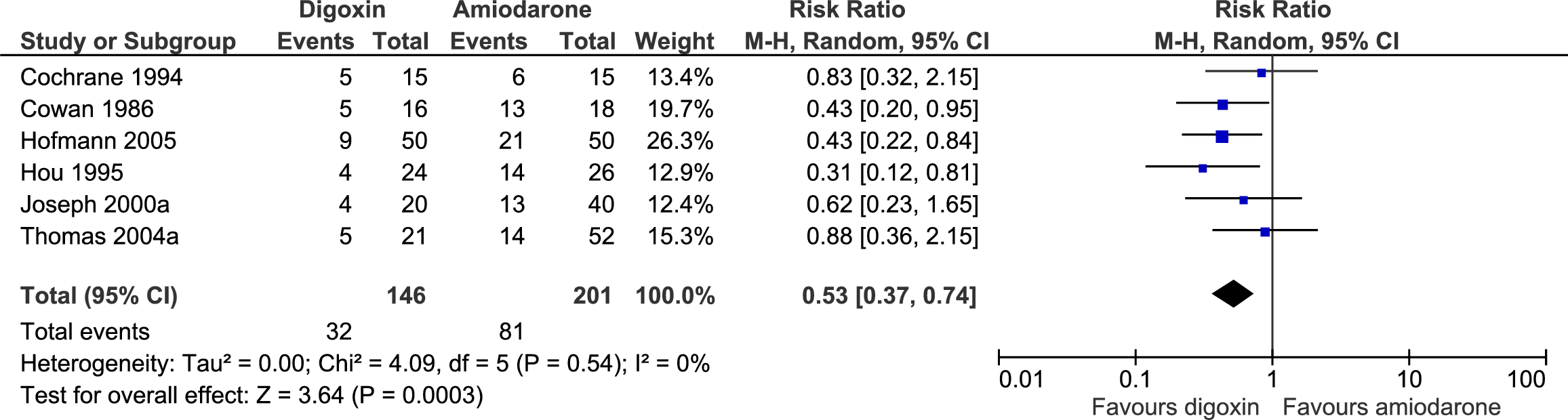

Supplement: S79 Fig — (TIF) [file pone.0193924.s082.tif]

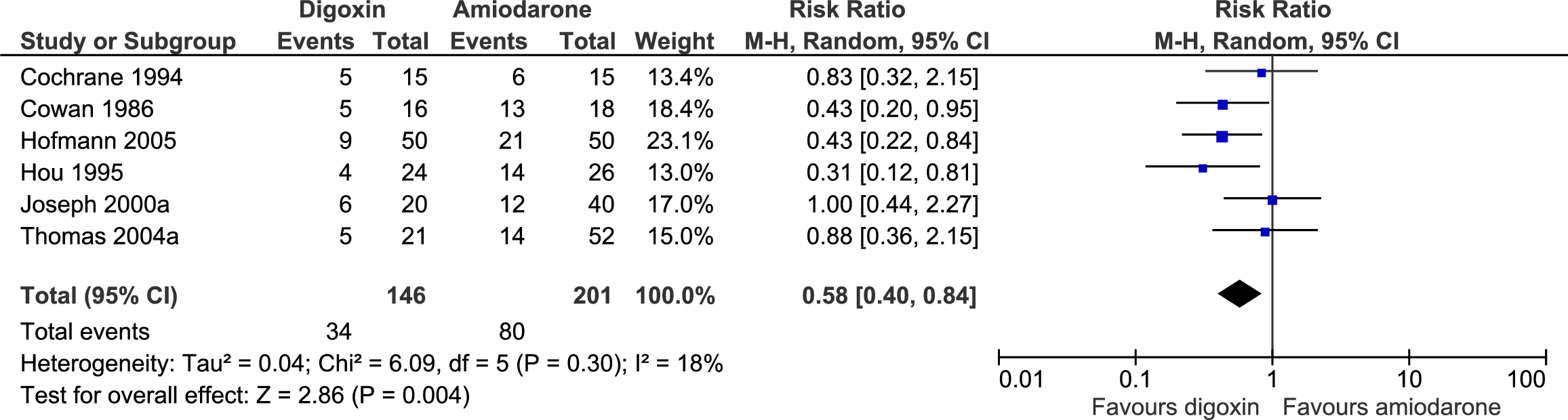

Supplement: S80 Fig — (TIF) [file pone.0193924.s083.tif]
