# Supplementary material for: Digoxin for atrial fibrillation and atrial flutter: A systematic review with meta-analysis and trial sequential analysis of randomised clinical trials
Source: PLoS One. 2018 Mar 8;13(3):e0193924. doi: 10.1371/journal.pone.0193924 (PMC5843263; doi:10.1371/journal.pone.0193924)
Supplement: S1 Table — (DOCX) [file pone.0193924.s084.docx]

**S1 Table. Inclusion- and exclusion criteria for each trial included.**

|  | **Inclusion criteria** | **Exclusion criteria** |
| --- | --- | --- |
| **Ang et al. (1990)** | - Chronic atrial fibrillation. - Normal resting heart rate. - Exercise tachycardia. - Episodes of bradycardia. | - Not mentioned. |
| **Baroffio et al. (1995)** | - Recent-onset atrial fibrillation. - Heart rate > 80 bpm. - No contraindications to either drug. - Without clinical heart failure. | - Ongoing antiarrhythmic treatment. - Therapy with digitalis. - Acute myocardial infarction in the previous month or unstable angina. - NYHA class III or IV heart failure. - Low cardiac output. - Hypotension (systolic blood pressure <100 mmHg). - Hyperthyroidism. - Known sick sinus syndrome. - Second- or third degree AV block. - Postoperative period following cardiac surgery. - Bifascicular block. - Chronic obstructive lung disease. - WPW-syndrome. - Obesity (>120 kg). - Assessed or assumed pregnancy. |
| **Bianconi et al. (1998)** | - Recent-onset atrial fibrillation. | - Digitalis therapy. - Class I or III antiarrhythmic therapy. - Myocardial infarction within the preceding month. - Postoperative period after heart surgery. - Unstable angina. - Clinical signs of heart failure. - Low cardiac output. - Clinical signs of hyperthyroidism. - Systolic blood pressure <100 mmHg. - Heart rate <80 bpm. - Bifascicular block. - Known sick sinus syndrome. - 2^nd^/3^rd^ AV-block in absence of a cardiac pacemaker. - WPW-syndrome. - Ascertained or presumed pregnancy. |
| **Botto et al. (1994)** | - Recent-onset atrial fibrillation. - Without heart failure. | - Concomitant use of digitalis, amiodarone, or other antiarrhythmic drugs. - Hypokalemia. - Recent myocardial infarction. - Complete bundle branch block. - Sick sinus syndrome. - AV block. - Ventricular preexcitation. |
| **Botto et al. (1995)** | - Recent-onset atrial fibrillation - Without clinical signs of heart failure. | - None mentioned. |
| **Cochrane et al. (1994)** | - Postoperative atrial fibrillation or flutter following myocardial revascularization, valve surgery, or combined procedures. | - Atrial fibrillation prior to surgery. - Poor ventricular contractility on pre-operative left ventriculogram. - Post-operative administration of beta-blocking agents. |
| **Cowan et al. (1986)** | - Atrial fibrillation complicating suspected or proven acute myocardial infarction. - Remaining in atrial fibrillation at the end of 1 hour, with a ventricular response rate in excess of 120 bpm. | - Receiving amiodarone or digoxin. - Chronic atrial fibrillation. - Rheumatic valvular disease. - Systolic blood pressure <90 mmHg. - Clinically significant pulmonary oedema. |
| **CRAFT-1 (1993)** | - History of documented atrial fibrillation. - Frequent (1 or more episodes/month), symptomatic, self-terminating episodes. | - History of atrial fibrillation requiring cardioversion on >1 occasion. - History of thromboembolism. - Symptoms such as syncope or angina related to atrial fibrillation. - Uncorrected electrolyte imbalance. - Serum potassium <3.8 mmol/L. - Abnormal thyroid function. - Renal, hepatic, pulmonary, or cardiac insufficiency. - Valvular heart disease. - Left atrial diameter >45 mm. - Myocardial infarction, unstable angina, or cardiac revascularisation in the preceding three months. - Known or suspected accessory atrioventricular pathway. - History of 2^nd^ or 3^rd^ degree AV-block. - Sinus node dysfunction. - Implanted cardiac pacemaker. - Hypertrophic cardiomyopathy. - Amiodarone or investigational drugs in the preceding three months. - Pregnancy, lactation, or of childbearing potential and not using contraception. |
| **DAAF (1997)** | - Persistent atrial fibrillation (7 days or more). | - Ongoing treatment with digitalis or antiarrhythmic drugs other than beta-blockers (including sotalol) or calcium channel blockers. - Sick sinus syndrome. - History of 2^nd^/3^rd^ AV-block without an artificial pacemaker. - WPW syndrome. - Heart rate <60 bpm. - Heart rate >170 bpm. - Ongoing myocardial infarction. - Myocardial infarction at four weeks or less prior to entry into study. - Haemodynamic instability. - Ongoing angina pectoris. - Serum potassium <3.3 mmol/L. - Serum creatinine > 300 µmol/L. |
| **DIGAF (1997)** | - Recent-onset atrial fibrillation. | - Prior antiarrhythmic drugs. - Suffering from acute coronary or pulmonary diseases. |
| **Falk et al. (1987)** | - Recent-onset atrial fibrillation. - Ventricular rate between 85 to 175 bpm. | - Use of digitalis or antiarrhythmic drugs. - Heart failure. - Acute myocardial infarction. - Unstable angina. - Preexcitation syndrome. - Thyrotoxicosis. - Hypokalaemia. - Renal impairment. - Severe metabolic disturbances. |
| **Hjelms et al. (1992)** | - Post-operative atrial fibrillation after open-heart surgery. | - Heart block. - Receiving digoxin treatment at the time when postoperative atrial fibrillation occurred. |
| **Hofmann et al. (2005)** | - Atrial fibrillation. - Mean ventricular rate above 135 bpm. | - Less than 18 years of age. - Baseline systolic blood pressure <100 mmHg. - Known thyroid function disorder. - Serum potassium <3.5 mmol/L. - Pretreatment with any antiarrhythmic drug with class I or class III properties. - History of torsades de pointes. - Chronic atrial fibrillation. - QTC interval of above 440 ms measured in the qualifying ECG. - Organised tachyarrhythmias such as atrial flutter. |
| **Holming et al. (2001)** | - Chronic atrial fibrillation. - Not treated with ant chronotropic drugs. | - None mentioned. |
| **Hou et al. (1995)** | - Persistent atrial fibrillation or flutter. - Ventricular rates above 130 bpm. | - Cardiogenic shock. - Hypotension (<80 mmHg) - WPW syndrome with atrial fibrillation or flutter. - RR-intervals <270 ms. - Sick sinus syndrome. - Episodes of paroxysmal atrial fibrillation or flutter that had lasted less than 24 hours in previous events. - Consumption of any antiarrhythmic drug. - Serum creatinine <3.5 mEq/L. - Severe hypoxia. - Severe metabolic acidosis. - Thyrotoxicosis. - Onset of atrial fibrillation or flutter within 24 hours after or during surgery. |
| **Innes et al. (1997)** | - Recent-onset atrial fibrillation. | - Ventricular rates less than 100 bpm or more than 200 bpm. - Allergy to digoxin, quinidine, or verapamil. - Hypotension (<90 mmHg) with evidence of end-organ hypoperfusion, including confusion, coma, severe angina, or acute myocardial infarction. - Conduction abnormalities, including 2^nd^ or 3^rd^ degree AV-block. - Sick sinus syndrome. - QRS interval greater than 12 ms. |
| **J-Land (2013)** | - Atrial fibrillation or flutter. - NYHA class III or IV. - Heart rate of 120 bpm or above. - LVEF 25 to 50%. | - Necessity for electrical cardioversion. - Serious valve stenosis. - Confirmed or suspected hyperthyroidism. - Implantable cardiac pacemaker and/or implantable defibrillator - Necessity for mechanical ventilation. - Cardiogenic shock (systolic blood pressure <90 mmHg). |
| **Jordaens et al. (1997)** | - Symptomatic recent-onset atrial fibrillation. - Heart rate > 100 bpm. | - Cardiac glycosides had been given within the last week. - Antiarrhythmic drug use in the last 72 hours. - Previous use of amiodarone. - Acute myocardial infarction. - Recent coronary artery bypass grafting. - Haemodynamic or respiratory instability. - Electrolyte disturbances. |
| **Joseph et al. (2000)** | - Recent-onset atrial fibrillation or flutter. - Ventricular rate > 100 bpm. - Serum kalium >3.5 mmol/L and <5.5 mmol/L. - Serum creatinine <0.2 mmol/L. | - Wide-complex tachycardia., such as WPW-syndrome. - Current beta blocker treatment. - Digoxin or sotalol treatment in the last week. - Amiodarone treatment within three months. - Hypotension (MAP <70 mmHg) - Previous adverse reaction to any of trial medications. - Known thyroid disease. - Asthma/bronchospasm with beta blocker. - Contraindication to anticoagulation. - Less than 18 years of age. - Left ventricular dysfunction. - Pregnancy. |
| **Schreck et al. (1997)** | - Recent-onset of atrial fibrillation or flutter. | - Systolic blood pressure <100 mmHg. - Allergy to calcium channel blockers or digoxin. - Current treatment with calcium channel blockers other than diltiazem. - Acute myocardial infarction with pulmonary congestion. - Digoxin toxicity. - Acute congestive heart failure (NYHA III or IV). - Cardiogenic shock. - Sick sinus syndrome. - Evidence of WPW-syndrome. - Rhythm other than atrial fibrillation. |
| **Shojaee et al. (2017)** | - Rapid atrial fibrillation. - Relative contraindication for calcium channel blockers and beta blockers. | - Unstable haemodynamics. - Chest pain. - Shortness of breath. - Heart failure. - Unconfirmed dysrhythmia. - Allergy to digoxin or amiodarone. - Underlying kidney or liver diseases. - Used antiarrhythmic drugs in the past 12 hours. |
| **Simpson et al. (2001)** | - Recent-onset atrial fibrillation. - Heart rate >100 bpm. | - Significant haemodynamic instability. - Unable to take oral medications. - Pre-excitation syndrome. - Currently on either digoxin, verapamil, or clonidine. |
| **Siu et al. (2009)** | - Acute symptomatic atrial fibrillation (<48 hours of onset) necessitating hospitalisation. - Ventricular rate >120 bpm. | - Ventricular rate >200 bpm. - Pre-excitation syndrome. - Hypotension (systolic blood pressure <90 mmHg). - Congestive heart failure. - Presence of implanted pacemaker and/or implantable cardioverter defibrillator. - Recent myocardial infarction. - Unstable angina. - Stroke or thromboembolism within the past six months. - Allergy or contraindication to the study medications. - Use of antiarrhythmic and/or atrioventricular nodal blocking drugs within last seven days (in case of amiodarone, within past three months). - Other major medical conditions including renal failure, respiratory failure, and bleeding disorders. |
| **Thomas et al. (2004)** | - Symptomatic recent-onset atrial fibrillation. | - Amiodarone or sotalol in the preceding month. - Had an adverse reaction on a trial drug. - Previously experienced atrial fibrillation while taking amiodarone or sotalol. - Asthma or chronic airway limitation. - Signs or symptoms of heart failure. - Known or suspected pulmonary fibrosis. - Pregnancy. - Uncorrectable hypotension (<90 mmHg). - Sick sinus syndrome. - Bradycardia (< 50 bpm). - QTC >450 ms. - Active hepatitis. - Postoperative patients (<1 month). |
| **Tisdale et al. (1998)** | - Post-operative atrial fibrillation. - Ventricular rate >100 bpm for at least 15 minutes within seven days after CABG. | - Systolic blood pressure <100 mmHg). - Severe congestive heart failure (NYHA III or IV). - Serum creatinine >2.0 mg/dL. - Serum potassium concentration <3.0 mg/dL. - Serum magnesium concentration <1.0 mg/dL. - Myocardial infarction within seven days. - History of sinus node dysfunction. - 2^nd^ or 3^rd^ AV-block in the absence of a functioning pacemaker. - WPW-syndrome. - Administration of digoxin, verapamil, diltiazem, or class Ia, Ic, og III antiarrhythmic drugs within five half-lives before administration of study drug. - Mechanically ventilated patients. - Receiving dopamine, dobutamine, or norepinephrine. |
| **Tse et al. (2001)** | - Chronic atrial fibrillation. | - Intolerance or contraindication to digoxin or amiodarone. - Amiodarone therapy in the past six months. - Clinically significant valvular heart disease. - Unstable angina. - Recent myocardial infarction in the past six months. - Class III or IV heart failure. - Sick sinus syndrome. - Implanted pacemaker. |
| **Van Noord et al. (2001)** | - Persistent atrial fibrillation. - Ventricular rate >90 bpm on resting ECG. - Planned electrical cardioversion within one month. | - History of 2^nd^ or 3^rd^ degree AV-block. - Sick sinus syndrome. - Heart failure (NYHA III or IV). - Unstable angina pectoris. - Current treatment with calcium channel blockers or digoxin. - Concomitant treatment with class I or III antiarrhythmic drugs (amiodarone should not have been used during the last three months). - Untreated hyperthyroidism or hypothyroidism. - Serious pulmonary, hepatic, hematologic, metabolic, renal, gastrointestinal, central nervous system, or psychiatric disease. - Pacemaker treatment. - Contraindications for oral anticoagulant drugs. - Less than 18 years or older than 85 years. |
| **Wattanasuwan et al. (2001)** | - Atrial fibrillation. - Uncontrolled ventricular rates. | - Patients with systolic blood pressure <90 mmHg. - Acute congestive heart failure. - Acute coronary syndromes. - Ventricular rates >200 bpm. - Coexisting unstable medical conditions (e.g., fever, sepsis, acute renal failure, acute hepatic failure, thyrotoxicosis, or ARDS). - Pre-excitation syndrome. - Allergy to diltiazem or digoxin. - Any antiarrhythmic drug use within one week before presentation. |
