# Supplementary material for: Digoxin for atrial fibrillation and atrial flutter: A systematic review with meta-analysis and trial sequential analysis of randomised clinical trials
Source: PLoS One. 2018 Mar 8;13(3):e0193924. doi: 10.1371/journal.pone.0193924 (PMC5843263; doi:10.1371/journal.pone.0193924)
Supplement: S2 Table — (DOCX) [file pone.0193924.s085.docx]

**S2 Table. Characteristics of each included, excluded, and ongoing study.**

**Characteristics of included studies.**

|  | **Study design** | **Type of atrial fibrillation** | **Type of control intervention(s)** | **Type of administration** | **Digoxin group (no. of participants)** | **Control group (no. of participants)** | **Maximum follow-up** |
| --- | --- | --- | --- | --- | --- | --- | --- |
| **Ang et al. (1990)** | Parallel, three-armed | Chronic | Xamoterol or placebo | Oral | Not stated | Not stated | 24 hours |
| **Baroffio et al. (1995)** | Parallel | Recent-onset | Propafenone | Intravenous | 25 | 25 | Discharge from hospital |
| **Bianconi et al. (1998)** | Parallel, three-armed | Recent-onset | Propafenone or placebo | Intravenous | 40 | 41 (propafenone);  42 (placebo) | One hour |
| **Botto et al. (1994)** | Parallel, four-armed | Recent-onset | Propafenone, flecainide, or placebo | Oral | 25 | 92 (propafenone); 34 (flecainide); 92 (placebo) | 24 hours |
| **Botto et al. (1995)** | Parallel, three-armed | Recent-onset | Propafenone or placebo | Oral | 34 | 36 (propafenone); 35(placebo) | 24 hours |
| **Cochrane et al. (1994)** | Parallel | Recent-onset | Amiodarone | Oral and intravenous | 15 | 15 | 24 hours |
| **Cowan et al. (1986)** | Parallel | Mixed | Amiodarone | Intravenous | 16 | 18 | Discharge from hospital |
| **CRAFT-1 (1993)** | Parallel | Paroxysmal | Placebo | Oral | 20 | 20 | Two months |
| **DAAF (1997)** | Parallel | Persistent | Placebo | Intravenous | 117 | 122 | 16 hours |
| **DIGAF (1997)** | Parallel | Recent-onset | Placebo | Not stated | 32 | 33 | 18 hours |
| **Falk et al. (1987)** | Parallel | Recent-onset | Placebo | Oral | 18 | 18 | 24 hours |
| **Hjelms et al. (1992)** | Parallel | Recent-onset | Procainamide | Intravenous | 15 | 15 | Not stated |
| **Hofmann et al. (2005)** | Parallel | Mixed | Amiodarone | Intravenous | 50 | 50 | Eight days |
| **Holming et al. (2001)** | Parallel | Chronic | Sotalol | Oral | 10 | 11 | One month |
| **Hou et al. (1995)** | Parallel | Persistent | Amiodarone | Intravenous | 24 | 26 | 24 hours |
| **Innes et al. (1997)** | Parallel | Recent-onset | Verapamil | Intravenous | 25 | 19 | Not stated |
| **J-Land (2013)** | Parallel | Mixed | Landiolol | Intravenous | 115 | 99 | Two hours |
| **Jordaens et al. (1997)** | Parallel | Recent-onset | Placebo | Intravenous | 20 | 20 | 12 hours |
| **Joseph et al. (2000)** | Parallel, three-armed | Recent-onset | Sotalol or amiodarone | Oral and intravenous | 40 | 40 (sotalol); 40 (amiodarone) | 48 hours |
| **Schreck et al. (1997)** | Parallel | Recent-onset | Diltiazem | Intravenous | 9 | 11 | Three hours |
| **Shojaee et al. (2017)** | Parallel | Not stated | Amiodarone | Intravenous | 42 | 42 | Not stated |
| **Simpson et al. (2001)** | Parallel, three-armed | Recent-onset | Verapamil or clonidine | Intravenous | 15 | 13 (verapamil); 12 (clonidine) | Six hours |
| **Siu et al. (2009)** | Parallel, three-armed | Recent-onset | Diltiazem or amiodarone | Intravenous | 50 | 50 (diltiazem); 50 (amiodarone) | 24 hours |
| **Thomas et al. (2004)** | Parallel, three-armed | Recent-onset | Sotalol or amiodarone | Oral and intravenous | 43 | 45 (sotalol); 52 (amiodarone) | 12 hours |
| **Tisdale et al. (1998)** | Parallel | Recent-onset | Diltiazem | Intravenous | 20 | 20 | 24 hours |
| **Tse et al. (2001)** | Parallel | Chronic | Amiodarone | Oral | 7 | 9 | 24 weeks |
| **Van Noord et al. (2001)** | Parallel | Persistent | Verapamil | Oral | 49 | 48 | One month |
| **Wattanasuwan et al (2001)** | Parallel | Mixed | No intervention beside co-intervention | Intravenous | 26 | 26 | 12 hours |

**Characteristics of excluded studies**

|  | **Reason for exclusion** |
| --- | --- |
| **Boman et al. (1983)** | Included both patients in sinus rhythm and atrial fibrillation. |
| **Channer et al. (1987)** | All the included participants were used both as an experimental group and as a control group. |
| **Coburn et al. (1979)** | All the included participants were used both as an experimental group and as a control group. |
| **Farshi et al. (1999)** | All the included participants were used both as an experimental group and as a control group. |
| **Fauchier et al. (2009)** | Not randomized. |
| **Halley et al. (1980)** | Not randomized. |
| **Khand et al. (2003)** | Only included patients that already received digoxin. |
| **Koh et al. (1995)** | Both the experimental group and the control group received digoxin. |
| **Lanas et al. (1995)** | All the included participants were used both as an experimental group and as a control group. |
| **Lang et al. (1983)** | All the included participants were used both as an experimental group and as a control group. |
| **Lewis et al. (1987)** | All the included participants were used both as an experimental group and as a control group. |
| **Lewis et al. (1988) I** | All the included participants were used both as an experimental group and as a control group. |
| **Lewis et al. (1988) II** | All the included participants were used both as an experimental group and as a control group. |
| **Lindholm et al. (2004)** | The experimental group and the control group did not receive the same amount of co-intervention. |
| **Maragno et al. (1988)** | The trial compared the wrong interventions. |
| **Moghadem et al. (2012)** | Not randomized. |
| **Pomfret et al. (1987)** | All the included participants were used both as an experimental group and as a control group. |
| **Roth et al. (1986)** | All the included participants were used both as an experimental group and as a control group. |
| **VERDICT (2006)** | The experimental group and the control group did not receive the same amount of co-intervention. |
| **Wong et al. (1990)** | All the included participants were used both as an experimental group and as a control group. |

**Characteristics of ongoing studies**

|  | **Expected finish date** | **Study design** | **Estimated number of participants** | **Type of atrial fibrillation** | **Type of control intervention** | **Maximum follow-up** |
| --- | --- | --- | --- | --- | --- | --- |
| **RATE-AF (2015)** | July 2019 | Parallel | 160 | Chronic | Bisoprolol | 12 months |
