# Supplementary material for: Digoxin for atrial fibrillation and atrial flutter: A systematic review with meta-analysis and trial sequential analysis of randomised clinical trials
Source: PLoS One. 2018 Mar 8;13(3):e0193924. doi: 10.1371/journal.pone.0193924 (PMC5843263; doi:10.1371/journal.pone.0193924)
Supplement: S3 Table — (DOCX) [file pone.0193924.s086.docx]

**S3 Table. Specific types of serious adverse events in each trial comparison.**

| **Trial** | **Year** | **DIGOXIN:**  **Serious adverse events** | **Proportion of participants with a serious adverse event (digoxin)** | **CONTROL:**  **Serious adverse events** | **Proportion of participants with a serious adverse event (control)** |
| --- | --- | --- | --- | --- | --- |
| **Ang et al. (I)** | 1990 | Not mentioned | - | Not mentioned | - |
| **Ang et al. (II)** | 1990 | Not mentioned | - | Not mentioned | - |
| **Baroffio et al.** | 1995 | - 12 hospitalisations | 12 out of 25 | - 6 hospitalisations | 6 out of 25 |
| **Bianconi et al. (I)** | 1998 | Not mentioned | - | Not mentioned | - |
| **Bianconi et al. (II)** | 1998 | Not mentioned | - | Not mentioned | - |
| **Botto et al. (I)** | 1994 | Not mentioned | - | Not mentioned | - |
| **Botto et al. (II)** | 1994 | Not mentioned | - | Not mentioned | - |
| **Botto et al. (III)** | 1994 | Not mentioned | - | Not mentioned | - |
| **Botto et al. (I)** | 1995 | Not mentioned | - | Not mentioned | - |
| **Botto et al. (II)** | 1995 | Not mentioned | - | Not mentioned | - |
| **Cochrane et al.** | 1994 | Not mentioned | - | Not mentioned | - |
| **Cowan et al.** | 1986 | - 4 deaths | 4 out of 16 | - 4 deaths | 4 out of 18 |
| **CRAFT-1** | 1993 | Not mentioned | - | Not mentioned | - |
| **DAAF** | 1997 | - 1 asystole - 1 left ventricular outflow obstruction | 2 out of 117 | None | 0 out of 122 |
| **DIGAF** | 1997 | Not mentioned | - | Not mentioned | - |
| **Falk et al.** | 1987 | Not mentioned | - | Not mentioned | - |
| **Hjelms et al.** | 1992 | - 1 ventricular fibrillation | 1 out of 15 | None | 0 out of 15 |
| **Hofmann et al.** | 2005 | - 2 deaths - 1 recurrent pulmonary embolism - 1 pulmonary oedema and coronary ischemia | 2 out of 50 | - 1 death | 1 out of 50 |
| **Holming et al.** | 2001 | Not mentioned | - | Not mentioned | - |
| **Hou et al.** | 1995 | None | 0 out of 24 | - 1 death - 1 heart failure | 2 out of 26 |
| **Innes et al.** | 1997 | - 1 rapid ventricular response associated with chest pain | 1 out of 22 | - 1 episode of hypotension and bradycardia | 1 out of 19 |
| **J-Land** | 2013 | - 1 sinus arrest - 1 diabetes insipidus - 1 pneumonia | 3 out of 107 | - 1 death - 1 heart failure - 1 embolic stroke | 2 out of 93 |
| **Jordaens et al.** | 1997 | - 1 sinus arrest | 1 out of 19 | None | 0 out of 20 |
| **Joseph et al. (I)** | 2000 | - 3 heart failures - 1 stroke | 4 out of 18 | None | 0 out of 40 |
| **Joseph et al. (II)** | 2000 | - 3 heart failures - 1 sinus arrest | 4 out of 18 | - 2 heart failures - 1 sinus arrest | 3 out of 39 |
| **Schreck et al.** | 1997 | Not mentioned | - | Not mentioned | - |
| **Shojaee et al.** | 2017 | Not mentioned | - | Not mentioned | - |
| **Simpson et al. (I)** | 2001 | Not mentioned | - | Not mentioned | - |
| **Simpson et al. (II)** | 2001 | Not mentioned | - | Not mentioned | - |
| **Siu et al. (I)** | 2009 | Not mentioned | - | Not mentioned | - |
| **Siu et al. (II)** | 2009 | None | 0 out of 25 | - Phlebitis | 1 out of 50 |
| **Thomas et al. (I)** | 2004 | - 1 stroke | 1 out of 22 | None | 0 out of 45 |
| **Thomas et al. (II)** | 2004 | None | 0 out of 21 | - 1 cardiogenic shock | 1 out of 52 |
| **Tisdale et al.** | 1998 | Not mentioned | - | Not mentioned | - |
| **Tse et al.** | 2001 | Not mentioned | - | Not mentioned | - |
| **Van Noord et al.** | 2001 | - 1 death | 1 out of 49 | - 3 deaths - 4 heart failures | 5 out of 48 |
| **Wattanasuwan et al.** | 2001 | Not mentioned | - | Not mentioned | - |
