# Supplementary material for: Digoxin for atrial fibrillation and atrial flutter: A systematic review with meta-analysis and trial sequential analysis of randomised clinical trials
Source: PLoS One. 2018 Mar 8;13(3):e0193924. doi: 10.1371/journal.pone.0193924 (PMC5843263; doi:10.1371/journal.pone.0193924)
Supplement: S4 Table — (DOCX) [file pone.0193924.s087.docx]

**S4 Table. Heart rate control in all patients regardless of type of rhythm.**

| **Outcome** | **Comparison** | **Trials** | **Participants** | **Mean difference (bpm)** | **TSA-adjusted CI** | **P value** | **I^2^ (%)** |
| --- | --- | --- | --- | --- | --- | --- | --- |
| **Heart rate control within six hours after treatment onset** | Digoxin vs. placebo | 1 | 33 | -25.00 | -67.19 to 17.19 | 0.0002 | - |
|  | Digoxin vs. beta blockers | 1 | 168 | 13.00 | 5.99 to 20.01 | <0.00001 | - |
|  | Digoxin vs. calcium antagonists | 2 | 60 | 29.30 | 0.36 to 58.24 | <0.00001 | 0 |
|  | Digoxin vs. amiodarone | 4 | 212 | 17.15 | 8.65 to 25.65 | <0.00001 | 32 |
| **Heart rate control six to 24 hours after treatment onset** | Digoxin vs. calcium antagonists | 1 | 40 | 14.00 | -20.10 to 48.10 | 0.03 | - |
|  | Digoxin vs. amiodarone | 3 | 112 | 5.96 | -49.33 to 61.24 | 0.39 | 76 |
